# A high-quality genome assembly highlights rye genomic characteristics and agronomically important genes

**DOI:** 10.1038/s41588-021-00808-z

**Published:** 2021-03-18

**Authors:** Guangwei Li, Lijian Wang, Jianping Yang, Hang He, Huaibing Jin, Xuming Li, Tianheng Ren, Zhenglong Ren, Feng Li, Xue Han, Xiaoge Zhao, Lingli Dong, Yiwen Li, Zhongping Song, Zehong Yan, Nannan Zheng, Cuilan Shi, Zhaohui Wang, Shuling Yang, Zijun Xiong, Menglan Zhang, Guanghua Sun, Xu Zheng, Mingyue Gou, Changmian Ji, Junkai Du, Hongkun Zheng, Jaroslav Doležel, Xing Wang Deng, Nils Stein, Qinghua Yang, Kunpu Zhang, Daowen Wang

**Affiliations:** 1 College of Agronomy, Longzi Lake Campus, Henan Agricultural University, Zhengzhou, China; 2 Peking University Institute of Advanced Agricultural Sciences, Weifang, China; 3 School of Advanced Agriculture Sciences and School of Life Sciences, State Key Laboratory of Protein and Plant Gene Research, Peking University, Beijing, China; 4 Biomarker Technologies Corporation, Beijing, China; 5 Agronomy College, Sichuan Agricultural University, Chengdu, China; 6 The State Key Laboratory of Plant Cell and Chromosome Engineering, Institute of Genetics and Developmental Biology, Chinese Academy of Sciences, Beijing, China; 7 Triticeae Research Institute, Sichuan Agricultural University, Chengdu, China; 8 Institute of Experimental Botany of the Czech Academy of Sciences, Centre of the Region Hana for Biotechnological and Agricultural Research, Olomouc, Czech Republic; 9 Leibniz Institute of Plant Genetics and Crop Plant Research (IPK), Seeland, Germany; 10 Center for Integrated Breeding Research (CiBreed), Department of Crop Sciences, Georg-August-University, Göttingen, Germany; 11 The State Key Laboratory of Wheat and Maize Crop Science, Center for Crop Genome Engineering, Henan Agricultural University, Zhengzhou, China

**Keywords:** Genomics, Plant genetics

## Abstract

Rye is a valuable food and forage crop, an important genetic resource for wheat and triticale improvement and an indispensable material for efficient comparative genomic studies in grasses. Here, we sequenced the genome of Weining rye, an elite Chinese rye variety. The assembled contigs (7.74 Gb) accounted for 98.47% of the estimated genome size (7.86 Gb), with 93.67% of the contigs (7.25 Gb) assigned to seven chromosomes. Repetitive elements constituted 90.31% of the assembled genome. Compared to previously sequenced Triticeae genomes, *Daniela*, *Sumaya* and *Sumana* retrotransposons showed strong expansion in rye. Further analyses of the Weining assembly shed new light on genome-wide gene duplications and their impact on starch biosynthesis genes, physical organization of complex prolamin loci, gene expression features underlying early heading trait and putative domestication-associated chromosomal regions and loci in rye. This genome sequence promises to accelerate genomic and breeding studies in rye and related cereal crops.

## Abstract (web summary)

A high-quality genome assembly of Weining rye sheds new light on gene duplications and their effects on starch biosynthesis genes, gene expression features underlying early heading trait and putative domestication-associated chromosomal regions.

## Main

Rye (*Secale cereale*, 2*n* = 2*x* = 14, RR) belongs to the genus *Secale* in the Triticeae tribe of the grass family *Poaceae*^1^. Although phylogenetically related to common wheat (*Triticum aestivum*, Ta) and barley (*Hordeum vulgare*, Hv), rye has unique characteristics in both agronomic performance and genome properties^1–4^.

Rye is well known for its strong tolerance to abiotic stresses and high adaptability to barren soils^2,5^. It is also characterized by potent resistance to many fungal diseases, which often elicit severe economic losses in global Triticeae crops^4,6^. The disease-resistance genes carried by the rye 1RS chromosome arm, transferred to the wheat genome through wide hybridization, have contributed greatly to the control of powdery mildew and stripe rust diseases in worldwide wheat production^4^. Moreover, rye is essential for developing triticale, a synthetic forage and promising food crop with higher biomass and yield level than rye^7^. Thus, rye is a valuable crop in many countries and a globally important genetic resource for wheat and triticale improvement.

The genome of rye is substantially larger than those of barley and diploid wheat species, and was estimated to be around 7.9 Gb, with transposon elements (TEs) constituting approximately 90% of the genome^8,9^. However, potential contributions of specific TEs to rye genome expansion remain to be resolved. To date, a high-quality reference genome sequence is still unavailable for rye. By contrast, genome assemblies were published for Ta and its diploid progenitor species *Triticum urartu* (Tu) and *Aegilops tauschii* (Aet)^10–13^. The genomes of wild emmer wheat (*Triticum turgidum* ssp. *dicoccoides*, WEW), Hv and durum wheat (*T. turgidum* ssp. *durum*) were also decoded in recent times^14–16^.

Weining rye, an early flowering variety cultivated in China, is outstanding because of its broad-spectrum resistance to both powdery mildew and stripe rust^17,18^. To understand the genetic and molecular basis of rye elite traits and to promote genomic and breeding studies in rye and related crops, we sequenced and analyzed the genome of Weining rye.

## Results

### Genome assembly and gene annotation

**Fig. 1:**
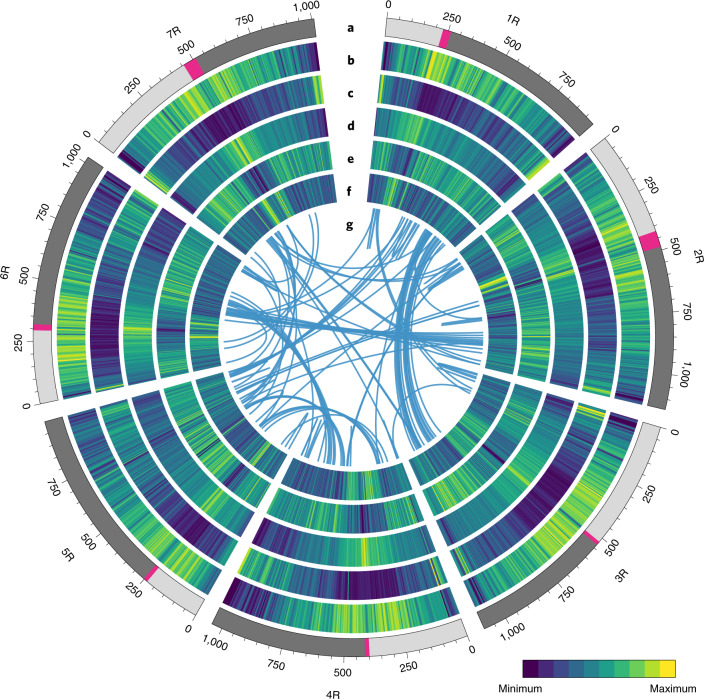
Genomic features of rye. Circos display of important features of the assembled Weining rye genome. The seven layers depict chromosome names and sizes, with centromere positions marked in red (**a**), TE density along each chromosome (**b**), density of HC genes (**c**), density of *Gypsy* retrotransposons (**d**), density of *Copia* retrotransposons (**e**), distribution of GC content in each chromosome (**f**) and links between syntenic genes (**g**). TE density was relatively low in the distal ends of chromosomes 1R–7R. *Gypsy* had the highest distribution density in the centromeric area and the lowest density toward the telomeric regions, whereas *Copia* exhibited the opposite distribution pattern.

**Extended Data Fig. 1:**
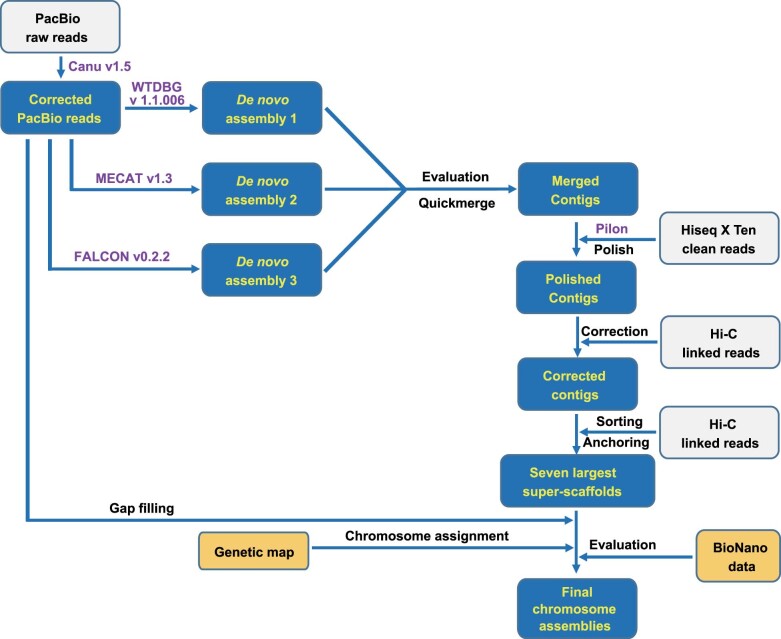
Pipeline for constructing the seven chromosome assemblies of Weining rye. Multiple sequencing technologies and assembly strategies were applied to construct the genome sequence of Weining rye (Supplementary Tables 1–6). First, 497.01 Gb of long reads (62× genome coverage) generated by single molecule real-time sequencing (Pacbio Sequel), together with 430 Gb Illumina paired-end clean reads (54× genome coverage), were jointly used to assemble the genome into 63,244 contigs with an N50 size of 480.35 kilobases (kb). Second, six high-throughput chromatin conformation capture (Hi-C) libraries were constructed and yielded 1.87 billion clean paired-end reads, over 90% of which could be mapped to the initial assembly. This three-dimensional proximity information allowed us to correct, order, orientate and anchor PacBio contigs into super-scaffolds, with the seven largest ones selected as candidates of the seven chromosomes of Weining rye (Extended Data Fig. 2 and Supplementary Table 5), which were then subjected to gap filling with corrected PacBio reads and manual adjustment. Third, a genetic map (Extended Data Fig. 3), developed using 295 F2 individuals derived from crossing Weining rye with another rye variety (Jingzhou) (Supplementary Data 1), was used to assign the seven super-scaffolds onto chromosomes, which yielded the final 1 R to 7 R chromosome-scale assemblies. Approximately 96.02% of the assembly was covered with BioNano molecules (97× genome coverage), and a high consistency was found between the genome assembly of Weining rye and the BioNano genomic reads (Supplementary Fig. 1).

**Extended Data Fig. 2:**
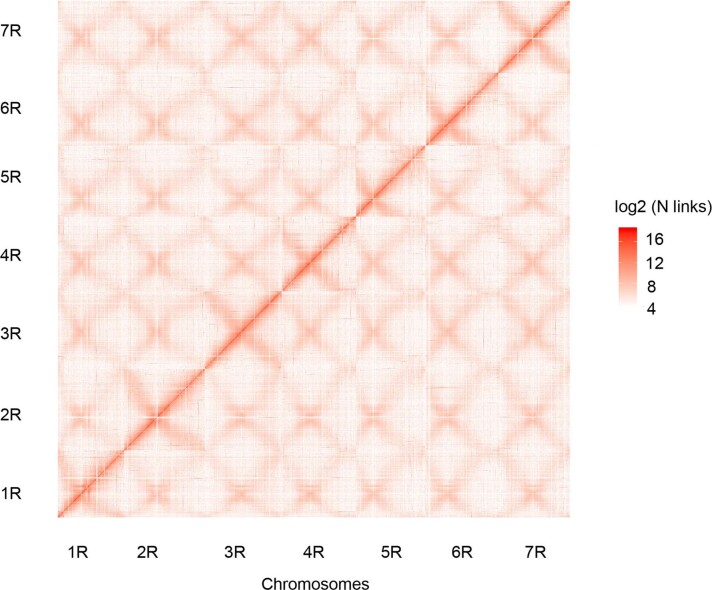
Hi-C contact map of the seven assembled Weining rye chromosomes (1 R - 7 R). Abundant intrachromosomal contacts were observed. Interchromosomal contacts were also found but with much decreased intensity.

**Extended Data Fig. 3:**
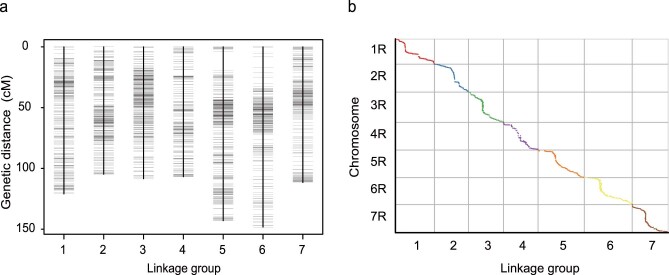
Genetic linkage map of Weining × Jingzhou rye varieties. Genetic linkage map (WJ) developed using 295 F_2_ plants derived from the crossing of two rye varieties (Weining × Jingzhou) (**a**) and alignment between the WJ linkage map and the physical map of the seven assembled chromosomes of Weining rye (**b**). In (a) the SNP markers anchored were 2,662, and the total genetic distance covered was 843.8 cM.

The genome size of Weining rye was estimated to be 7.86 Gb using flow cytometry^19^, which is consistent with the previous estimate of around 8 Gb for rye genome size^9,20^. To construct the genome sequence of Weining rye, we integrated the datasets generated by long-range PacBio RS II and short-read Illumina sequencing, as well as those from chromatin conformation capture (Hi-C), genetic mapping and BioNano analysis (Supplementary Tables 1–6, Supplementary Fig. 1, Supplementary Data 1 and Extended Data Figs. 1–3). In total, the assembled genome sequence was 7.74 Gb, with a scaffold N50 size of 1.04 Gb, representing 98.47% of the estimated genome size of Weining rye, of which 7.25 Gb was anchored on seven pseudo-chromosomes (1R–7R), accounting for 93.67% of the assembled genome sequence (Table 1 and Fig. 1). The assembled chromosome size was larger for 2R, 3R, 4R, 6R and 7R (above 1 Gb) and comparatively smaller for 1R (0.94097 Gb) and 5R (0.99891 Gb) (Fig. 1 and Supplementary Table 7). By comparison, none of the chromosomes assembled for Tu, Aet, WEW, Ta or Hv were larger than 0.9 Gb (Supplementary Table 7).

**Table 1:** Statistics of Weining rye genome assembly and annotation

Genomic feature	Value	Percentage (%)^a^
Estimated genome size	7.86 Gb	
Assembled genome size	7.74 Gb	
Assembled genome percentage	98.47%	
Sequence assigned to chromosomes	7.25 Gb	93.67
Number of scaffolds	18,028	
N50 scaffold length	1.04 Gb	
N90 scaffold length	0.95 Gb	
Number of contigs	63,244	
N50 contig length	480.35 kb	
N90 contig length	37.50 kb	
Longest contig	9.02 Mb	
GC content	45.89%	
Size of retrotransposons	5,996.0 Mb	77.46
Size of DNA transposons	912.7 Mb	11.79
Other repeats	81.9 Mb	1.06
Size of total repeat sequences	6,990.6 Mb	90.31
Number of HC genes	45,596	
Number of HC transcripts	84,179	
Mean length of HC genes	4,864.34 bp	
Total length of HC genes	223,740,142 bp	

^a^The percentages were each calculated based on the assembled genome size (7.74 Gb).

The accuracy of the Weining genome assembly is supported by the following findings. First, the physical maps of chromosomes 1R–7R were highly consistent with a chromosomal linkage map constructed with two winter rye cultivars (Lo7 and Lo225)^2^, with Spearman’s rank correlation coefficient reaching 0.99 (*P* < 2.2 × 10^−16^) (Supplementary Fig. 2). Second, 97.45% of the 169,717 pyrosequencing reads previously reported for Lo7 (ref. ^2^), could be mapped to the Weining assembly with an average sequence identity of 97.71% and a mean sequence coverage of 97.27%. Finally, 99.77% of the 2,769,537,530 Illumina paired-end reads generated in the present study could be mapped onto the assembly (Supplementary Table 8). During this mapping, we identified 242,455 homozygous SNPs and 218,570 homozygous indels, indicating that the nucleotide accuracy rate of the assembly was 99.99%; we also found 19,215,912 heterozygous SNPs and 1,530,913 heterozygous indels, suggesting that the heterozygosity rate of the Weining genome was 0.26% (Supplementary Table 9).

**Extended Data Fig. 4:**
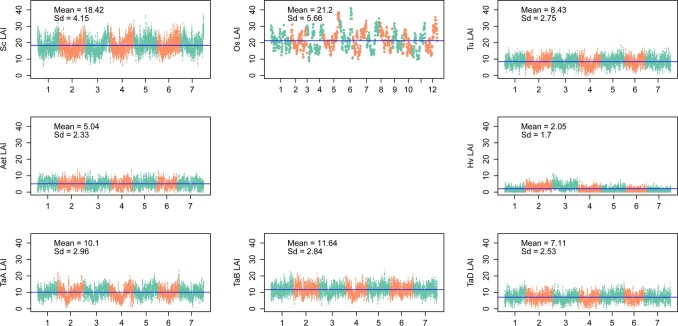
Evaluation of genome assemblies by LTR Assembly Index (LAI). The x-axes show the chromosomes of each genome. LAI scores, represented by the dots, were calculated using 3 Mb-sliding windows with 300-kb steps. The blue line indicates whole genome average of LAI score. Sc, *Secale cereale* (Weining rye); Os, *O. sativa* ssp. *japonica* (Nipponbare); Tu, *T. urartu* (G1812); Aet, *Ae. tauschii* (AL8/78); Hv, *Hordeum vulgare* (Morex); TaA, TaB and TaD, the A, B and D subgenomes of common wheat (Chinese Spring).

The long terminal repeat (LTR) Assembly Index (LAI), which evaluates the contiguity of intergenic and repetitive regions of genome assemblies based on the intactness of LTR retrotransposons (LTR-RTs)^21^, of the Weining genome assembly was 18.42, which was substantially higher than the LAI values obtained for the wheat and barley genomes under comparison (Extended Data Fig. 4). Furthermore, we identified 1,393 (96.74%, Supplementary Table 10) of the 1,440 conserved BUSCO genes^22^. Thus, the Weining rye genome sequence is of high quality in both intergenic and genic regions.

We annotated 86,991 protein-coding genes, including 45,596 high-confidence (HC) and 41,395 low-confidence genes (Table 1 and Supplementary Fig. 3), based on ab initio prediction and supporting evidence from transcriptome data and reference protein sequences from other plant genomes (Supplementary Fig. 3 and Supplementary Tables 11 and 12). The total number of HC transcripts (including splicing variants) identified for the HC genes was 84,179 (Table 1). Furthermore, we annotated 34,306 microRNA, 14,226 long non-coding RNA, 11,486 transfer RNA and 1,956 small nucleolar RNA species throughout the Weining genome assembly. The average intron length of Weining HC genes was the longest among 11 grass genomes, but the mean sizes of exons and coding sequences were similar between the compared genomes (Supplementary Table 13).

### Analysis of TEs

A total of 6.99 Gb, representing 90.31% of the Weining assembly, was annotated as TEs, which included 2,671,941 elements belonging to 537 families (Table 1 and Supplementary Table 14). This TE content was clearly higher than those previously reported for Ta (84.70%)^10^, Tu (81.42%)^11^, Aet (84.40%)^12^, WEW (82.20%)^14^ or Hv (80.80%)^15^. The LTR-RTs, including *Gypsy*, *Copia* and unclassified retrotransposon elements, were the dominant TEs and occupied 84.49% of the annotated TE content and 76.29% of the assembled Weining genome; CACTA DNA transposons were the second most abundant TE, constituting 11.68% of the annotated TE content and 10.55% of the assembled Weining genome.

**Fig. 2:**
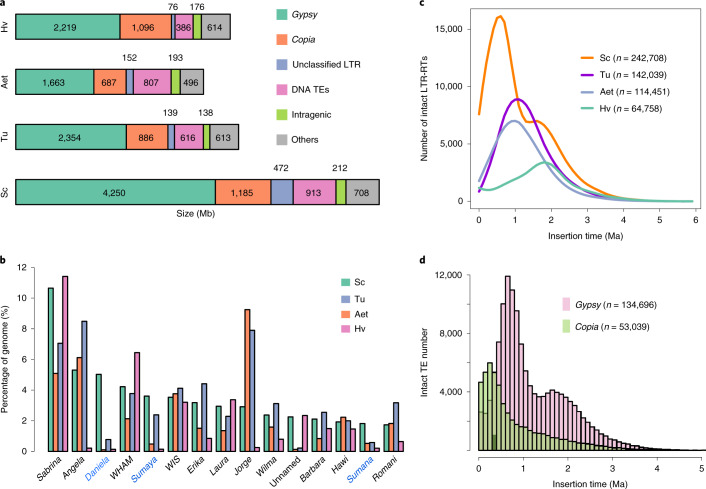
Analysis of TEs in rye. **a**, Genomic constituents in Weining rye (Sc) in comparison with those in Tu, Aet and Hv. Note that the six constituents, especially *Gypsy*, *Copia* and unclassified LTR TEs, were much more abundant in Weining rye than in Tu, Aet or Hv. **b**, The top 15 families of TEs in Weining rye and the percentages of these families in Weining rye, Tu, Aet and Hv. Three *Gypsy* LTR families, *Daniela*, *Sumaya* and *Sumana*, showed increased abundance in Weining rye relative to those in Tu, Aet and Hv. **c**, Temporal patterns of LTR-RT insertion bursts in Weining rye as compared to those in Tu, Aet and Hv. The number of intact LTR-RTs used for each species is given in parentheses. **d**, Insertion bursts of *Gypsy* and *Copia* elements in Weining rye. The numbers of intact elements used for this analysis are provided in parentheses.

Cross-genome comparisons with Tu, Aet and Hv showed that LTR-RTs, especially *Gypsy* elements, contributed the most to the genome expansion of Weining rye (Fig. 2a). Weining rye had ~2.52 Gb more of LTR-RTs than did barley, and this contributed 85.42% to the 2.95-Gb increase in the genome size of rye versus barley. The top 15 abundant TE families (11 *Gypsy*, three *Copia* and one CACTA) together represented about 56.5% of the assembled Weining genome, with the most abundant elements being from *Sabrina*, a family of non-autonomous *Gypsy* retrotransposons comprising 10.5% of the Weining assembly (Fig. 2b). Three LTR-RT families (*Daniela*, *Sumaya* and *Sumana*) exhibited substantially elevated abundance in Weining rye relative to those in Tu, Aet and Hv, with *Daniela* displaying the greatest elevation (Fig. 2b). *Daniela* accounted for 5.03% of the Weining assembly but less than 0.8% of those of Tu, Aet and Hv; *Sumaya* made up 3.61%, 2.38%, 0.48% and 0.14% of the genomes of Weining rye, Tu, Aet and Hv, respectively; and *Sumana* occupied 1.82% of the Weining genome but less than 0.6% of the genomes of Tu (0.58%), Aet (0.52%) and Hv (0.21%) (Fig. 2b).

A distinct bimodal distribution was found for the insertion times of intact LTR-RTs in Weining rye, whereas a unimodal distribution was observed for Tu, Aet and Hv (Fig. 2c). Weining rye had a comparatively high proportion of recent LTR-RT insertions, with the peak of amplification appearing around 0.5 million years ago (Ma), which was the most recent among the four species; the other peak, occurring approximately 1.7 Ma, was older and also seen in barley (Fig. 2c). At the superfamily level, we found very recent bursts of *Copia* elements in Weining rye at 0.3 Ma, while amplifications of *Gypsy* retrotransposons dominantly shaped the bimodal distribution pattern of LTR-RT burst dynamics (Fig. 2d).

Therefore, recent large-scale bursts of retrotransposons around 0.3–0.5 Ma, including the *Gypsy* retroelements *Daniela*, *Sumaya* and *Sumana*, contributed directly to rye genome expansion. Consistent with our analysis, past studies also showed that increases in the abundance of specific retrotransposon families can lead to plant genome expansion over short periods of time^23–25^. Identification of greatly enlarged TE families in rye may stimulate deeper studies of the dynamic changes of TEs and their consequences on genome expansion and function in Triticeae.

### Investigation of rye genome evolution and chromosome synteny

**Fig. 3:**
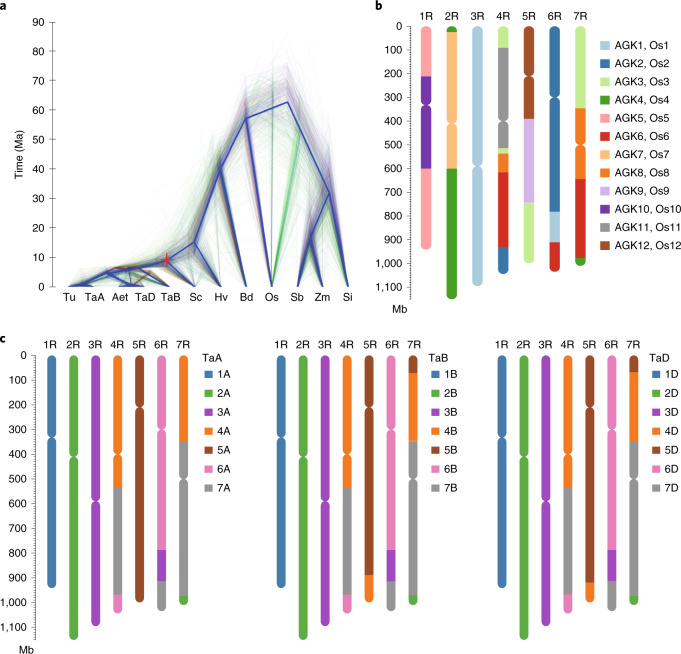
Evolutionary and chromosome synteny analyses of the rye genome. **a**, Phylogeny and divergence time estimate of rye, as investigated with a gene tree constructed using 2,517 single-copy orthologous genes conserved between Weining rye and 11 other grass genomes. Os, rice; Sb, *S. bicolor*; Si, *S. italica*; Zm, *Z. mays*. **b**, Probable evolutionary scenario of rye chromosomes according to the AGK model proposed by Murat et al.^31^. The rye chromosomes (1R–7R) are presented with a color code to show different segments from the ancestral grass chromosomes (AGK1–AGK12), which are referenced by the 12 chromosomes of rice (Os1–Os12). Chromosome 3R was derived from AGK1 or Os1, and a segment of this chromosome was translocated to 6RL. Chromosome 1R evolved from a nested insertion of AGK10 or Os10 into AGK5 or Os5, and 2R evolved by a nested insertion of AGK7 or Os7 into AGK4 or Os4. Chromosome 4R evolved by fusions of AGK11 or Os11 with the segments from AGK2 or Os2, AGK3 or Os3, AGK6 or Os6 and AGK8 or Os8, and 5R evolved by a fusion between AGK9 or Os9 and AGK12 or Os12 and acquisition of a segment from AGK3 or Os3. Chromosome 6R was mainly derived from AGK2 or Os2 and further fusions with the segments from AGK1 or Os1 and AGK6 or Os6. Lastly, 7R evolved mainly from AGK8 or Os8, with additional fusions of the segments from AGK3 or Os3, AGK4 or Os4 and AGK6 or Os6. **c**, Chromosome synteny between rye and the three subgenomes of common wheat (TaA, TaB and TaD). Syntenic chromosomes (or chromosomal segments) are labeled with the same color.

We computed 2,517 single-copy orthologous genes by comparing the genomes of Weining rye, Tu, Aet, Ta (subgenomes TaA, TaB and TaD), Hv, *Oryza sativa* ssp. *japonica* (Os), *Brachypodium distachyon* (Bd), *Zea mays*, *Sorghum bicolor* and *Setaria italica*. Phylogenetic and molecular dating investigations with these genes revealed that the divergence between rye and diploid wheats took place after the separation of barley from wheat, with the divergence times for the two events being approximately 9.6 and 15 Ma, respectively (Fig. 3a). These values were older than those based on chloroplast sequences^26^ but close to the higher end of the estimates based on *Acc* homoeoloci^27^.

All grass species experienced three ancient whole-genome duplications, which resulted in 12 ancestral grass karyotype (AGK) chromosomes that are largely preserved in modern-day rice^28–31^. We therefore used the rice genome as an ancestral reference to investigate the chromosomal evolution of Weining rye. A total of 23 large syntenic blocks enfolding 10,949 orthologous gene pairs between Weining rye and rice were identified (Supplementary Table 15), which enabled us to deduce the arrangements of ancestral chromosome segments in 1R–7R (Fig. 3b). In essence, 3R was derived from a single ancient chromosome, AGK1 or Os1, although a segment of this chromosome was translocated to 6RL; 1R and 2R were each formed by two ancestral chromosomes, with 1R involving a nested insertion of AGK10 or Os10 into AGK5 or Os5 and 2R forming by nested insertion of AGK7 or Os7 into AGK4 or Os4; 4R, 5R, 6R and 7R were each derived from at least three ancestral chromosomes via complex translocations (Fig. 3b and Supplementary Table 15). In the comparison of the Weining assembly with the three subgenomes of common wheat, 22,648, 22,212 and 22,830 orthologous gene pairs, representing 51.98%, 50.98% and 52.40% of the HC genes of TaA, TaB and TaD, respectively, were identified (Supplementary Table 16). These orthologous genes facilitated the identification of syntenic blocks between rye and common wheat chromosomes (Fig. 3c). Chromosomes 1R, 2R and 3R were entirely collinear with group 1, 2 and 3 chromosomes of wheat, respectively. In 4R, three regions showing collinearity with parts of 4(A, B, D), 7(A, B, D) or 6(A, B, D) were found. Chromosome 5R was entirely collinear with 5A and partly collinear with 5B and 5D due to the fusion of translocated 4B or 4D segments at the distal ends of 5BL or 5DL. In 6R, three regions displaying collinearity with parts of 6(A, B, D), 3(A, B, D) or 7(A, B, D), were observed. Chromosome 7R was partly collinear with 7A, 7B and 7D; the non-collinear regions in 7A, 7B and 7D were caused by two translocations (from 4A and 2A) to 7A and three translocations (from 5B or 5D, 4B or 4D and 2A or 2B) to 7B or 7D (Fig. 3c). Together, the above data will encourage the use of rye in comparative genomic research of grasses and in future wide hybridization studies between rye and common wheat.

### Analysis of gene duplications and their impact on starch biosynthesis genes

**Fig. 4:**
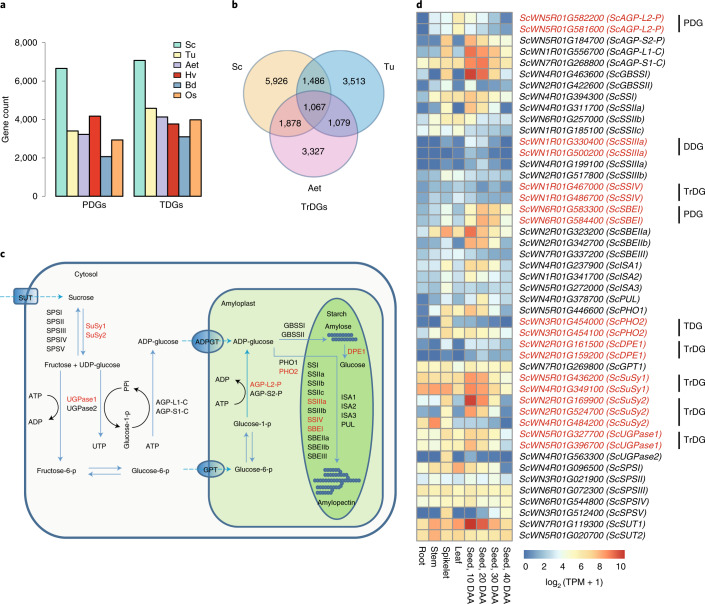
Analysis of rye gene duplications and their impact on the diversity of SBRGs. **a**, Higher numbers of proximally duplicated genes (PDGs) and tandemly duplicated genes (TDGs) in rye as compared with those in Tu, Aet, Hv, Bd and rice. **b**, Venn diagram showing the numbers of specific and shared transposed duplicated genes between Weining rye, Tu and Aet, computed using barley as a reference. **c**, A diagram illustrating the main enzymatic reactions catalyzed by the protein products of different SBRGs (definitions for abbreviations can be found in Supplementary Table 18). PPi, inorganic pyrophosphate; p, phosphate. **d**, Expression patterns of SBRGs in the root, stem, leaf, spike and developing grain tissues of Weining rye. Root, stem, leaf and spike samples were collected at the heading stage. The grain samples were collected at 10, 20, 30 and 40 d after anthesis (DAA), respectively. TPM, transcripts per million. In **c**,**d**, the nine genes shown in red exhibit different types of duplications relative to their counterparts in the A, B and D subgenomes of common wheat. Note that the SBRG duplicates often showed expression differences in different organs (**d**). DDG, dispersed duplicated gene.

The chromosomally located HC genes of Weining rye were analyzed with MCScanX software^32^, which yielded 4,217 singletons, 23,753 dispersed duplicated genes, 6,659 proximally duplicated genes, 7,077 tandemly duplicated genes and 1,866 segmentally duplicated genes (Fig. 4a and Supplementary Table 17). Notably, the numbers of tandemly duplicated genes and proximally duplicated genes in Weining rye were both higher than those found for Tu, Aet, Hv, Bd and Os (Supplementary Table 17).

The increased TE content of Weining rye (Fig. 2a) led us to investigate transposed duplicated genes (TrDGs), which are induced by TE activities and constitute a major part of the dispersed duplicated genes. We identified 10,357 TrDGs in Weining rye using Hv as a reference, which was substantially larger than the number of TrDGs in Tu (7,145) or Aet (7,351) (Fig. 4b). The TrDGs unique to Weining rye (5,926) were also more numerous than those specifically found for Tu (3,513) or Aet (3,327) (Fig. 4b).

We next investigated gene duplications in rye starch biosynthesis-related genes (SBRGs). Of the Weining rye SBRGs identified (Fig. 4c and Supplementary Table 18), nine had one or two duplicated copies on the same chromosome or a different chromosome. Transposed duplication occurred for five (*ScSSIV*, *ScDPEI*, *ScSuSy1*, S*cSuSy2* and *ScUGPaseI*) SBRGs, while tandem, proximal and dispersed duplications occurred for one (*ScPHO2*), two (*ScAGP-L2-p* and *ScSBE1*) and one (*SSIIIa*) SBRGs, respectively (Fig. 4c). The duplicates of the same SBRGs often showed differences in expression (Fig. 4d and Supplementary Data 2). For example, the parental copy of *ScSuSy2* (*ScWN2R01G169900*) was strongly expressed in developing grains at 10 and 20 d after anthesis but with fairly low expression in stem and root tissues; one of its transposed duplicates (*ScWN4R01G484200*), however, had little expression in developing grains but was rather highly expressed in stem and root tissues.

Thus, it appears that rye genome expansion is accompanied by larger numbers of gene duplications. The increased TE bursts in rye may have led to an elevated number of TrDGs. As illustrated by analyzing SBRGs, the various types of gene duplications can enrich the diversities of rye genes functioning in important biological processes. Elucidation of the whole set of rye SBRGs will facilitate their use in improving yield potential and nutritional quality traits. The new changes in rye SBRGs may provide new enzyme activities for manipulating plant starch biosynthesis and properties.

### Dissection of rye seed storage protein gene loci

Similar to wheat and barley, rye accumulates abundant prolamin-type seed storage proteins (SSPs) in endosperm tissues. Although four chromosomal loci (*Sec-1* to *Sec-4*) specifying rye SSPs were identified, their structures remain to be fully elucidated^33,34^. We therefore dissected rye SSP loci and genes using the Weining genome assembly.

**Fig. 5:**
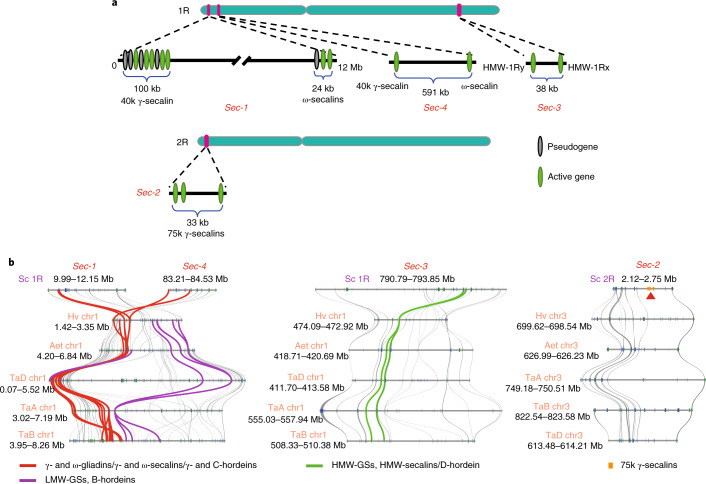
Analysis of rye secalin loci. **a**, Structure of *Sec-1*, *-2*, *-3* and *-4* loci and the secalin genes carried by them, with *Sec-1*, *-3* and *-4* located on chromosome 1R and *Sec-2* located on chromosome 2R. The size of each locus is shown, and the types of secalins specified by the four loci are also indicated. The physical position of each *Sec* locus on the chromosome (chr) is provided in **b**. **b**, Microsynteny analysis between four secalin loci and their respective collinear counterparts in barley (Hv), Aet and the three subgenomes of common wheat (TaA, TaB and TaD). The collinear SSP genes and the syntenic flanking genes are connected by colored and gray lines, respectively. The arrowhead indicates the three 75k γ-secalin genes resided in the Weining rye *Sec-2* locus. HMW-GSs, HMW glutenin subunits; LMW-GSs, low-molecular-weight glutenin subunits.

**Extended Data Fig. 5:**
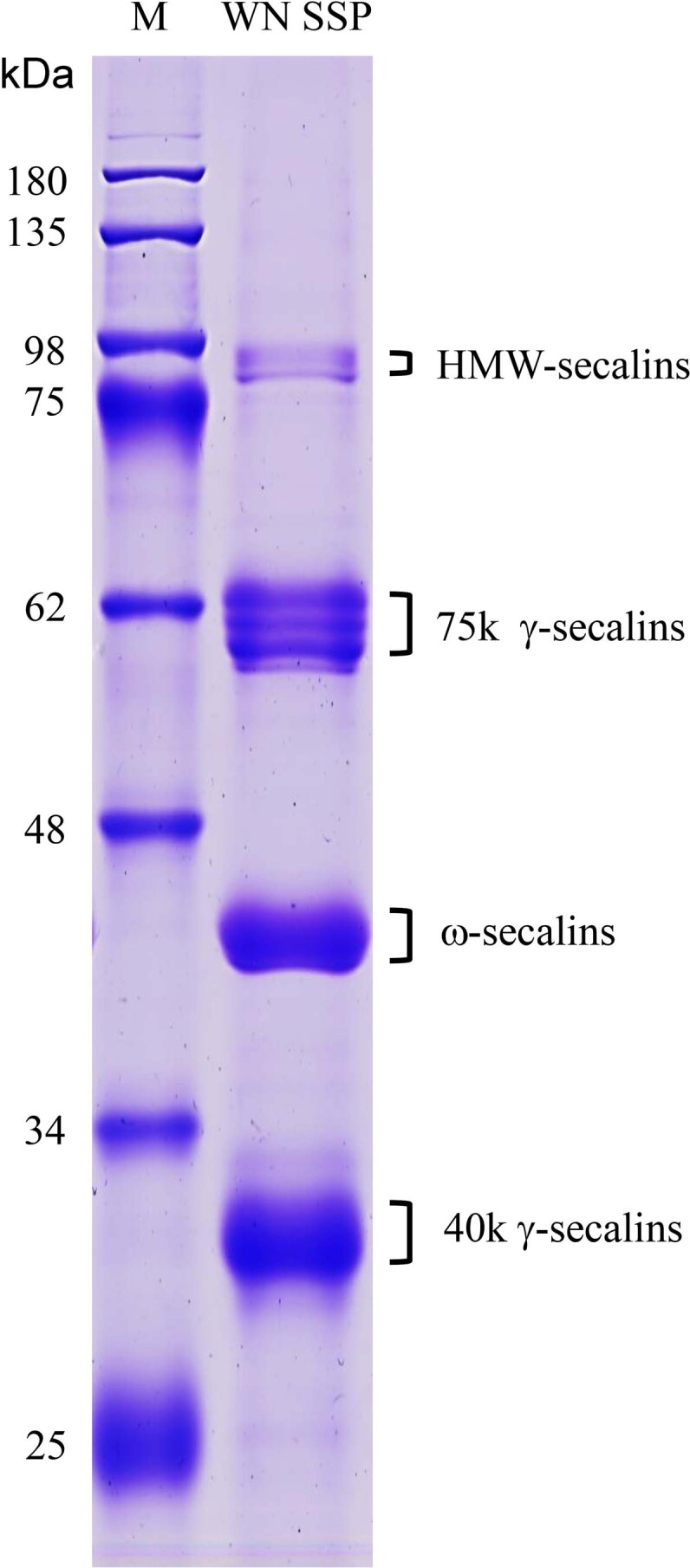
SDS-PAGE analysis of seed storage proteins extracted from the mature grains of Weining rye. Four types of secalins, that is, HMW-secalins, 75k γ-secalins, ω-secalins, and 40k γ-secalins, were detected. HMW-secalins and 75k γ-secalins are encoded by *Sec-3* and *Sec-2*, respectively, whereas ω-secalins and γ-secalins are specified by *Sec-1* and *Sec-4*. The secalins exhibited anomalous electrophoretic mobilities in SDS-PAGE because of carrying highly repetitive, proline- and glutamine-rich motifs in their proteins (Supplementary Table 19). M, protein size marker (kDa); WN SSP, Weining rye seed storage protein. The data shown were reproducible in three independent experiments. Source data

The secalin genes we identified are listed in Supplementary Table 19. As shown in Fig. 5a, the size of *Sec-1* was ~12 Mb, and it contained two separate clusters of genes encoding γ- or ω-secalins, with a total of seven active gene members. *Sec-4* was ~591 kb and carried two active genes coding for one γ- and one ω-secalin. *Sec-3* was ~38 kb and harbored two active genes specifying one y- and one x-type of high-molecular-weight (HMW)-secalin (HMW-1Rx and HMW-1Ry), respectively. *Sec-2* was about 33 kb and comprised three active genes encoding 75k γ-secalins. In agreement with these results, SDS–polyacrylamide gel electrophoresis (PAGE) analysis indicated that mature Weining rye grains accumulated two HMW-secalins, three 75k γ-secalins and high amounts of ω-secalins and 40k γ-secalins (Extended Data Fig. 5).

**Extended Data Fig. 6:**
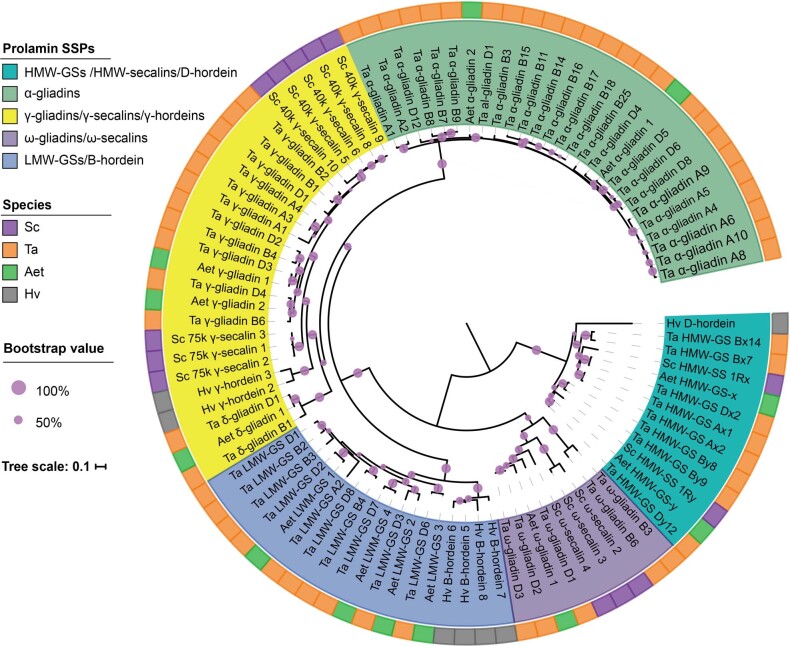
Phylogenetic analysis of rye secalins and homologous SSPs in barley (Hv), *Ae. tauschii* (Aet) and the three subgenomes of Chinese Spring wheat (TaA, TaB and TaD). A typical neighbor joining phylogenetic tree showing five distinct clusters of prolamin seed storage proteins (SSPs), that is, HMW glutenins/secalins/hordeins, α-gliadins, γ-gliadins/secalins/hordeins, ω-gliadins/secalins, and LMW-GSs/B-hordeins. The GenBank accession numbers for the compared SSPs are listed in Supplementary Table 20.

*Sec-1* and *Sec-4* were syntenic with the wheat chromosomal region carrying γ- and ω-gliadin genes and the barley chromosomal region harboring γ- and C-hordein genes (Fig. 5b). However, no orthologs of the wheat low-molecular-weight glutenin subunit or barley B-hordein genes were found in Weining rye (Fig. 5b and Supplementary Table 20), indicating the deletion of chromosome segments carrying such genes during rye evolution. The *Sec-3* region specifying rye HMW-secalins was collinear with the barley locus carrying the D-hordein gene and the wheat homoeologous loci harboring HMW glutenin subunit genes (Fig. 5b). The 75k γ-secalins specified by *Sec-2* were phylogenetically related to wheat γ-gliadins and barley γ-hordeins (Extended Data Fig. 6), but the wheat and barley chromosomal segments collinear to *Sec-2* contained no gliadin or other annotated SSP genes (Fig. 5b). Lastly, we did not find α-gliadin genes in the Weining genome assembly, which is compatible with the suggestion that α-gliadin genes evolved only recently in wheat and closely related species after the divergence of wheat from rye^35^. These SSP analysis results clarify the structure and composition of secalin loci, which will assist future efforts to refine the processing and nutritional qualities of rye, triticale and wheat.

### Examination of transcription factor and disease-resistance genes

**Extended Data Fig. 7:**
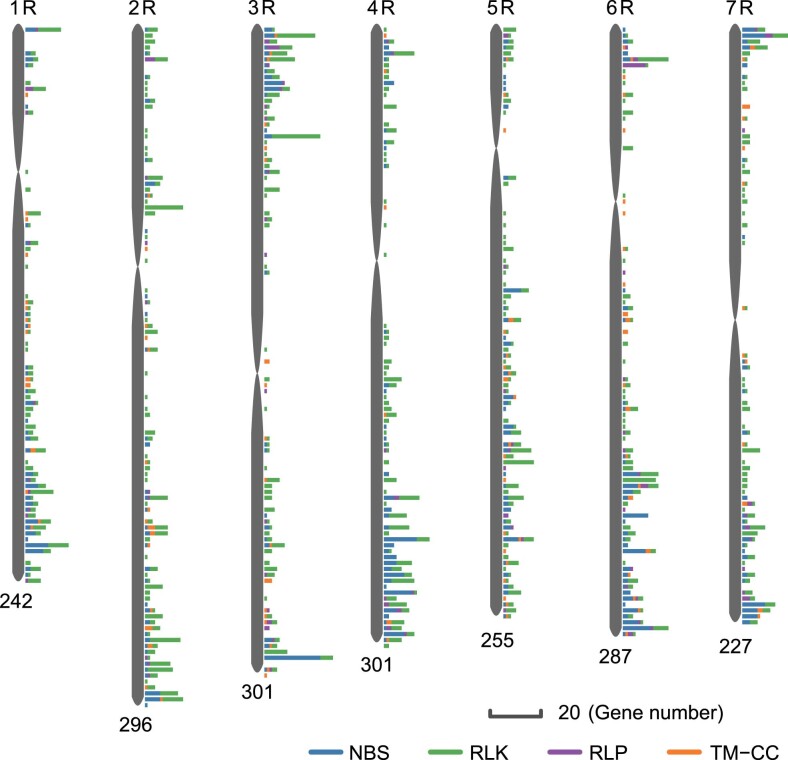
Distribution patterns of different types of predicted disease resistance associated (DRA) genes along the seven assembled chromosomes of Weining rye (1R-7R). The number of DRA genes on each chromosome is written in blue. In addition, there were 80 DRA genes found in the unassigned scaffolds, bringing the total number of DRA genes to 1,989 (see Supplementary Data 3 for a full list of the 1,989 DRA genes).

We predicted transcription factor (TF) genes in Weining rye and eight other grasses using the iTAK pipeline^36^. Of the 65 families of annotated TF genes, Weining rye had more members than other grasses in 28 families, with comparatively large increases for all three families of Apetala2–ethylene-responsive factor (AP2–ERF) TF genes (Supplementary Table 21). Weining rye had more disease-resistance-associated (DRA) genes (1,989, Supplementary Data 3) than did Tu (1,621), Aet (1,758), Hv (1,508), Bd (1,178), Os (1,575) or the A (1,836), B (1,728) or D (1,888) subgenomes of common wheat (Supplementary Table 22). The number of DRA genes was highest for chromosomes 2R–4R (296–301), intermediate for 1R and 5R (242–255) and comparatively low for 7R (227) (Extended Data Fig. 7). Considering the crucial importance of AP2–ERF TFs and DRA genes in plant responses to abiotic and biotic adversities^37–39^, the revelations presented above may facilitate efficient genetic studies and molecular improvement of stress tolerance and disease resistance in rye and related crops.

### Investigation of gene expression features associated with early heading trait

**Fig. 6:**
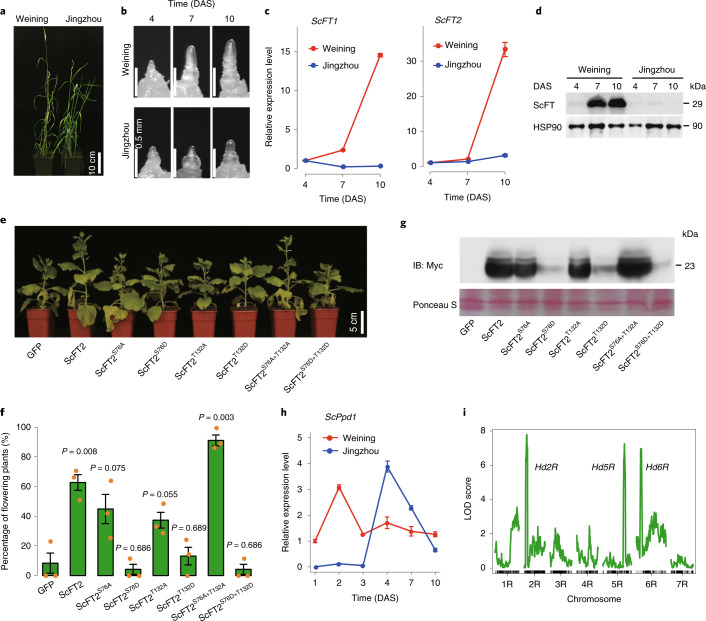
Gene expression features associated with the early heading trait of Weining rye. **a**, Different heading behavior between Weining and Jingzhou rye plants under long-day conditions. **b**, Differences in shoot apex development between Weining and Jingzhou plants at 4, 7 and 10 DAS. **c**, Higher expression of *ScFT* genes in Weining plants than that in Jingzhou plants at 7 and 10 DAS. **d**, Increased accumulation of the ScFT protein in Weining plants at 7 and 10 DAS, detected using an ScFT-specific antibody, with immunodetection of heat shock protein (HSP)90 as a loading control. **e**, Effects of potato virus X-mediated ectopic expression of Myc-tagged ScFT2 and six derivative dephosphomimic (S76A, T132A and S76A+T132A) or phosphomimic (S76D, T132D and S76D+T132D) mutant proteins on tobacco plant growth, with GFP as a control. **f**, Differences in tobacco flowering in plants expressing GFP (control), ScFT2 and six mutant proteins. The percentages of flowering plants are presented as mean ± s.e.m. of *n* = 3 independent experiments, with *P* values calculated using two-tailed *t*-tests. **g**, Immunodetection of Myc-tagged ScFT2 and six derivative mutant proteins in the panel of tobacco plants shown in **e**. ScFT2 levels accumulating in tobacco (~23 kDa) were lower than those in rye (~29 kDa) (**d**), probably due to different phosphomodifications of ScFT in tobacco and rye (Supplementary Note). **h**, Dissimilar expression profiles of *ScPpd1* in Weining and Jingzhou plants at 6 DAS. **i**, Detection of heading date QTL using 295 F_2_ individuals prepared from Weining × Jingzhou rye varieties. In **c**,**h**, each data point is the mean ± s.d. of five samples taken from *n* = 5 plants, and the results shown were reproducible in *n* = 3 independent quantitative PCR with reverse transcription (RT–qPCR) experiments. In **d**,**g**, the results shown were typical of *n* = 3 independent immunoblotting assays. Source data

In this work, we observed that Weining rye was heading 10–12 d earlier than Jingzhou rye under long-day conditions (Fig. 6a), which correlated with a more rapid development of the shoot apical meristem of Weining rye (Fig. 6b). Because of the key role of the flowering locus T (*FT*) gene in flowering-time control in higher plants^40^, we examined *FT* expression in the two lines.

**Extended Data Fig. 8:**
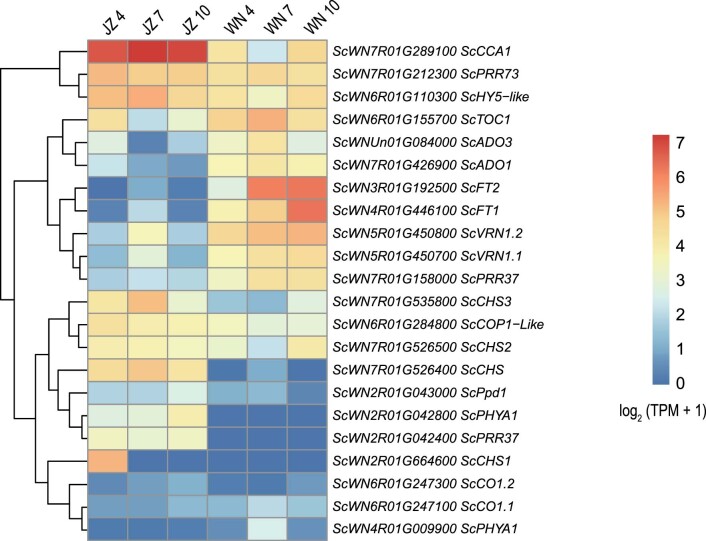
Different expression patterns of the genes related to the genetic control of heading date (flowering time) in Weining (WN, early heading) and Jingzhou (JZ, late heading) rye plants. These differentially expressed genes were identified using the transcriptome analysis data (Supplementary Data 4). 4, 7, and 10 indicate days after sowing.

**Extended Data Fig. 9:**
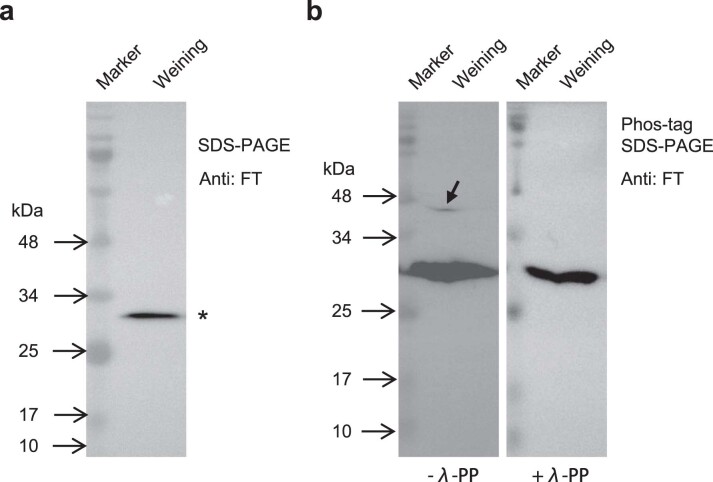
Investigation of the molecular mass of ScFT protein and evidence for its phosphorylation. (**a**) Immunoblotting detection of ScFT in Weining rye leaf tissues at 7 days after sowing. Total leaf proteins (10 μg) were separated using 12% SDS-PAGE, followed by immunoblotting with an antibody against the peptide QLGRQTVYAPGWRQ conserved in ScFT1 and ScFT2. Based on the protein size markers, the ScFT protein detected (asterisk) had a molecular mass of ~29 kDa. (**b**) Detection of ScFT phosphorylation using Phos-tag SDS-PAGE coupled with immunoblotting. Total leaf proteins (20 μg), with or without a treatment by λ protein phosphatase for 30 min at 30 °C, were separated using 12% Phos-tag SDS-PAGE, followed by immunoblotting with ScFT specific antibody. A ScFT phosphoprotein band (arrow) was specifically detected for the sample that was not treated by λ protein phosphatase. The data shown were reproducible in three independent experiments. Source data

Two *FT* genes with relatively high expression levels under long-day conditions, *ScFT1* (*ScWN4R01G446100*) and *ScFT2* (*ScWN3R01G192500*), were annotated in the Weining genome assembly. The expression levels of *ScFT1* and *ScFT2* were significantly higher in Weining plants than those in Jingzhou plants at 7 and 10 d after sowing (DAS) (Fig. 6c, Extended Data Fig. 8 and Supplementary Data 4). Consistently, rye FT (ScFT) proteins accumulated to relatively high levels in Weining plants but were barely detectable in Jingzhou rye at 7 and 10 DAS (Fig. 6d). Surprisingly, the size of ScFT proteins detected in rye (~29 kDa) was larger than the calculated molecular mass of ScFT1 or ScFT2 (both around 19 kDa) (Fig. 6d and Extended Data Fig. 9a), indicating potential post-translational modification of ScFT proteins. Analysis using Phos-tag SDS–PAGE, which is highly efficient at detecting phosphoproteins^41^, showed that ScFT was phosphorylated in Weining rye (Extended Data Fig. 9b).

Two amino acid residues in ScFT2 (S76 and T132), strictly conserved among the main FT proteins in grass species and *Arabidopsis thaliana*, were predicted to be phosphorylated (Supplementary Fig. 4). We therefore mutated the two residues and created a series of dephosphomimic (S76A, T132A and S76A+T132A) and phosphomimic sites (S76D, T132D and S76D+T132D) for ScFT2. When ectopically expressed in tobacco using a potato virus X-based viral vector, ScFT2 and the dephosphomimic double mutant ScFT2^S76A+T132A^ consistently enhanced tobacco growth relative to free GFP (control) and other ScFT2 mutants (Fig. 6e). Compared to GFP, ectopic expression of ScFT2 and the three dephosphomimic mutants (ScFT2^S76A^, ScFT2^T132A^ and ScFT2^S76A+T132A^) promoted the percentage of flowering plants, which was especially evident for ScFT2^S76A+T132A^, but such promotion was not observed when expressing the three phosphomimic mutants (ScFT2^S76D^, ScFT2^T132D^ or ScFT2^S76D+T132D^) (Fig. 6f). Immunoblotting assays showed that ScFT2, ScFT2^S76A^, ScFT2^T132A^ and ScFT2^S76A+T132A^ accumulated to similarly high levels in the tobacco plants, but the amounts of ScFT2^S76D^, ScFT2^T132D^ and ScFT2^S76D+T132D^ were very low (Fig. 6g). Hence, alteration of the conserved S76 and T132 residues affected the ability of ScFT2 to control plant flowering, which was associated with altered ScFT2 protein stability. To our knowledge, previous studies have not documented FT phosphorylation and its impact on flowering-time control. Our finding provides a new avenue to more comprehensively explore the molecular and biochemical mechanisms underlying the control of plant flowering by FT proteins.

We further investigated the expression of the photoperiod (*Ppd*) gene, which positively regulates *FT* expression under long-day conditions^40,42^. One gene expressing *Ppd*, *ScPpd1* (*ScWN2R01G043000*), was found in the transcriptome analysis of Weining and Jinzhou plants (Extended Data Fig. 8). This gene was expressed very early in Weining rye, with the peak of expression detected at 2 DAS; by contrast, *ScPpd1* expression occurred relatively late in Jingzhou plants and peaked at 4 DAS (Fig. 6h). In line with the involvement of the product of *ScPpd1* in regulating rye heading date, we detected a major quantitative trail locus (QTL) (*Hd2R*) with an logarithm of the odds (LOD) score of 8.19, explaining 12.16% of the heading date variation, in the chromosomal region harboring *ScPpd1* using the F_2_ population of Weining × Jingzhou plants (Fig. 6i and Supplementary Data 1). The same analysis also identified another two heading date QTL, *Hd5R* and *Hd6R*, located on chromosomes 5R and 6R, respectively (Fig. 6i). *Hd2R*, *Hd5R* and *Hd6R* together explained 33.63% of phenotypic variance, with the alleles from Weining rye exhibiting earliness additive effects (Supplementary Data 1). The identification of *Hd2R*, *Hd5R* and *Hd6R* is in line with the discovery of heading date QTL on chromosomes 2R, 5R and 6R in previous studies^43,44^.

### Mining of chromosomal regions and loci potentially involved in rye domestication

**Fig. 7:**
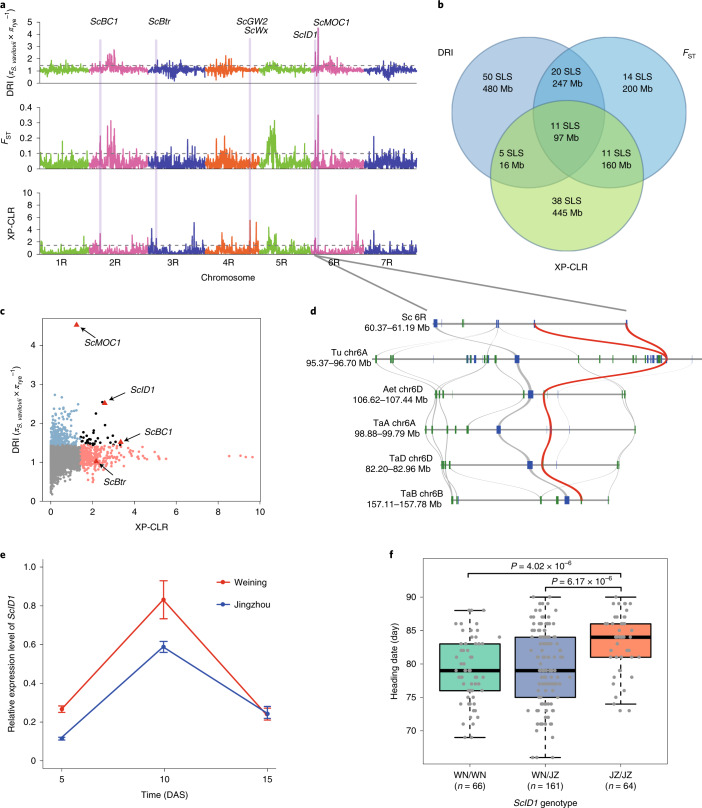
Analysis of chromosomal regions and loci potentially related to rye domestication. **a**, Putative selection sweeps revealed using three different methods, with dashed lines indicating thresholds for significant selection sweeps (top 5% outliers). *ScBC1*, *ScBtr*, *ScGW2*, *ScMOC1*, *ScID1* and *ScWx* were found in the putative sweeps. **b**, Specific and shared selection sweeps (SLS) uncovered by different methods. **c**, Selection sweeps associated with *ScBC1*, *ScBtr*, *ScMOC1* or *ScID1* uncovered by joint DRI and XP-CLR analysis. **d**, Microsynteny analysis of collinear *ID1* loci in rye and related Triticeae genomes. Red and gray lines connect orthologous *ID1* or flanking genes. **e**, Different expression profiles of *ScID1.1* and *ScID1.2* genes in Weining and Jingzhou rye plants at 3 DAS, revealed by RT–qPCR. Each value is the mean (±s.d.) of five samples taken from *n* = 5 plants, and the results shown were reproducible in *n* = 3 separate assays. **f**, Boxplots for days to heading, based on the genotype of the *ScID1* locus in the Weining × Jingzhou F_2_ population (*n* = 66 for WN/WN, *n* = 161 for WN/JZ and *n* = 64 for JZ/JZ). Box edges represent 0.25 and 0.75 quantiles, respectively, with the median values shown by bold lines. The lower and upper whiskers correspond to the minima values, at most the first quartile − 1.5 × interquartile range (IQR) or the maxima values no further than the third quartile + 1.5 × IQR. All data are shown by gray dots, with statistical analysis performed using two-tailed *t*-tests.

Analysis of domestication genes can accelerate the understanding and improvement of crop traits^45,46^. However, little progress has been made in the molecular analysis of such genes in rye. Here we tested the possibility of mining domestication-related chromosomal regions and loci in rye by genome-wide selection sweep analysis using 123,647 SNPs segregated between cultivated rye and *Secale vavilovii* (Methods). The number of significant selection sweep signals (top 5% threshold, with at least ten SNPs in each putative sweep region) was 86 by the diversity reduction index (DRI) method, 56 by genome-wide scan of fixation index (*F*_ST_) and 65 by the cross-population composite likelihood ratio (XP-CLR) method, with 11 signals identified by all three methods (Fig. 7a–c and Supplementary Data 5). Syntenic comparison with rice and barley, the genomes of which are better characterized than that of rye, uncovered a number of loci in the putative selection sweeps, including *ScBC1*, *ScBtr*, *ScGW2*, *ScMOC1*, *ScID1* and *ScWx*, for which the rice and barley orthologs were functionally analyzed (Fig. 7a–c and Supplementary Data 5). The detection of the *ScBtr-*containing chromosomal region by XP-CLR is consistent with the selection of orthologous *Btr* loci in the domestication of wheat and barley^14,47^.

**Extended Data Fig. 10:**
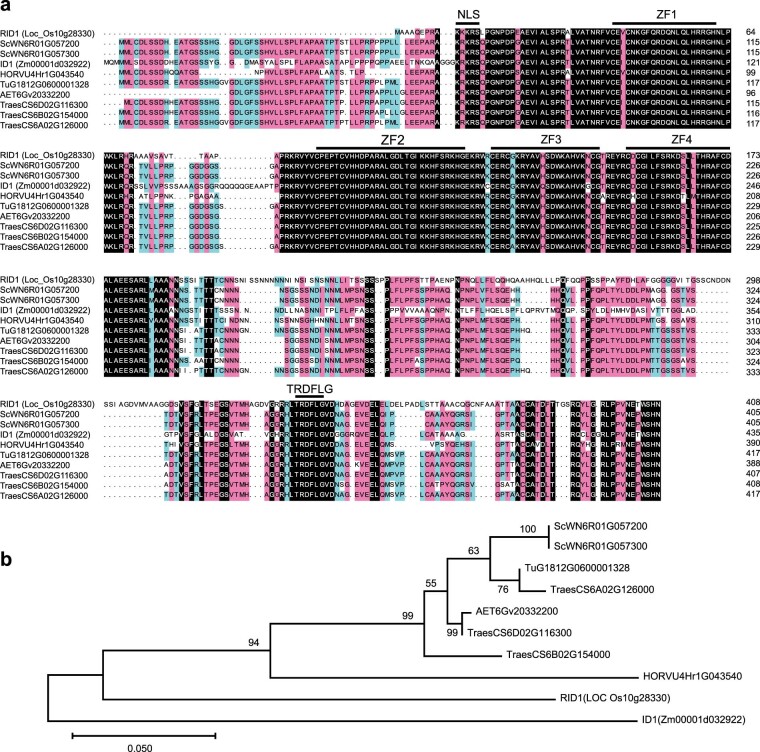
Evolutionary analysis of ScID1.1 and ScID1.2. **(a)** Alignment of the deduced amino acid sequences of ScID1.1 (ScWN6R01G057200), ScID1.2 (ScWN6R01G057300) and their orthologs in rice (RID1, LOC Os10g28330), maize (ID1, Zm00001d032922), barley (HORVU4Hr1G043540), *T. urartu* (TuG1812G0600001328), *Ae. tauschii* (AET6Gv20332200), and the three subgenomes of common wheat (TraesCS6A02G126000, TraesCS6B02G154000, and TraesCS6D02G116300). The nuclear localization signal (NLS), the four zinc-finger domains (ZF1 to ZF4) and the ‘TRDFLG’ motif are labeled. The residues are color coded according to their conservancy. (**b)** Phylogenetic tree of ID1 proteins from representative grasses as inferred using the Maximum Likelihood method and a JTT matrix-based model in MEGA X website. The tree with the highest log likelihood (−2146.66) is shown. The bootstrap values (1000 times) are shown next to the branches. The tree is drawn to scale, with branch lengths measured in the number of substitutions per site. All positions containing gaps and missing data were eliminated (complete deletion option). There were a total of 334 positions in the final dataset.

The *ScID1* locus, residing in the 6RS putative sweep region and detected by all three methods (DRI = 2.55, *F*_ST_ = 0.18, XP-CLR = 2.59) (Fig. 7a,c and Supplementary Data 5), contained a pair of tandemly duplicated *ScID1* paralogs (*ScWN6R01G057200* and *ScWN6R01G057300*, hereafter referred to as *ScID1.1* and *ScID1.2*) with an identical coding sequence (Fig. 7d). The deduced ScID1.1 and ScID1.2 proteins exhibited substantial identities to maize INDETERMINATE1 (ID1) (63.19%) and rice INDETERMINATE1 (RID1) (65.34%) (Extended Data Fig. 10), both of which were found to regulate the switch from vegetative to floral development^48–50^. Remarkably, the tandem duplication of *ID1* was observed only in Weining rye, whereas the *ID1* orthologs in wheat and closely related species were all present as single-copy genes (Fig. 7d). The expression levels of *ScID1.1* and *ScID1.2* were higher in young leaves of Weining rye than in those of Jingzhou rye at 5 and 10 DAS (Fig. 7e). Furthermore, in the F_2_ population of Weining (WN) × Jingzhou (JZ), the mean heading date of *ScID1*^JZ/JZ^ homozygous plants was significant later than that of *ScID1*^JZ/WN^ or *ScID1*^WN/WN^ individuals (Fig. 7f), which is consistent with the late flowering phenotype of Jingzhou rye relative to that of Weining rye (Fig. 6a).

The above data indicate possible involvement of the product of *ScID1* in regulating heading date and the probable selection of *ScID1* by domestication in rye. In line with our findings, a recent study discovered the selection of flowering-time genes during soybean domestication, and it was suggested that domestication selection of flowering-time genes may allow proper adjustment of crop maturity and thus better adaptation to the growth environment^51^. However, considering that the *ScID1*-containing 6R region identified by all three methods is quite large (~12 Mb, Supplementary Data 5), further work is needed to verify whether the *ScID1* locus might indeed function in heading date control and be selected during rye domestication.

## Discussion

Through the complementary sets of analyses described above, we generated new insights into the genomic characteristics of rye and its genes involved in agronomic trait control, identifying potentially useful chromosome regions and loci for further studies of the genetic basis of rye domestication. Therefore, the Weining genome assembly is of high value for deciphering rye genome biology, deepening comparative cereal genomic research and accelerating the genetic improvement of rye and related cereal crops.

## Methods

### Plant materials and fluorescence in situ hybridization assay

The Weining rye line used for genome analysis was selfed for 18 generations, and its genome was confirmed to carry seven pairs of chromosomes using fluorescence in situ hybridization (FISH) (Supplementary Fig. 5). Jingzhou rye is another population variety cultivated in Hubei, China^52^. Rye plants were grown under greenhouse conditions with day and night temperatures of 25 °C and 20 °C and a photoperiod consisting of light for 16 h and dark for 8 h. FISH assays of Weining rye root tip cells were accomplished as detailed previously using the fluorescently labeled probes pSC119.2 and (AAC)5 (ref. ^53^). The Weining × Jingzhou genetic population was prepared using Weining as the female parent. The F_2_ individuals were cultivated on the experimental farm from early March to late June of 2018.

### Genome sequencing

Thirteen paired-end libraries (150 bp) with insert sizes of ~270 bp were constructed according to the manufacturer’s instructions. A total of 430 Gb of short reads was obtained for genome survey and polishing. PacBio sequencing libraries were constructed as recommended by Pacific Biosciences. DNA fragments of about 10–50 kb were selected using BluePippin electrophoresis. Next, libraries were constructed and sequenced on the PacBio Sequel system with P6-C4 chemistry. A total of 120 SMRT cells were sequenced, producing 497 Gb of raw data. Hi-C libraries were created using a previously described method^15^. Six Hi-C fragment libraries, including five DpnII and one HindIII libraries, with fragment sizes ranging from 300 to 700 bp, were constructed and sequenced on the Illumina X Ten platform. A total of 1,869,066,895 paired reads (560 Gb of raw data) were generated.

### Genome assembly

For processing raw PacBio polymerase reads, sequencing adaptors were removed, and reads with low quality and short length were filtered using the PacBio SMRT Analysis package with stringent parameters (readScore, 0.75; minSubReadLength, 500). The obtained 497 Gb of high-quality PacBio subreads were corrected using the error correction module embedded in Canu (version 1.5) with the parameter ‘correctedErrorRate’ set to 0.045. The corrected reads were used for contig assembly by wtdbg (https://github.com/ruanjue/wtdbg) with the ‘wtdbg-cns -t 64 -k 15’ setting, FALCON (version 0.2.2) with ‘ovlp_HPCdaligner_option, -v -B128 -e.96 -l2400 -s100 -k18 -h1024 -M8 -T4’ parameters and MECAT (version 1.3) with the settings ‘corOutCoverage=60, corMhapSensitivity=high, correctedErrorRate=0.02’. The assembly results generated by the three assemblers were merged together using the Quickmerge (version 0.2) package with the ‘-hco 5.0 -c 1.5 -l 100000 -ml 5000’ setting, using the wtdbg contigs as reference input. For contig polishing, the Illumina paired-end reads (430 Gb) from Weining rye were mapped to the initial contigs using BWA (version 0.7.10-r789); polishing was performed by Pilon (version 1.22) using at least three iterations with the parameter ‘--mindepth 10 --changes --fix bases’. This step corrected 2,171,903 SNPs, 359,604 insertions and 1,145,074 deletions. Subsequently, a pre-assembly was executed for the error-corrected contigs using Hi-C data. Briefly, adaptor sequences in raw Hi-C reads were trimmed with Cutadapt (version 1.0), and low-quality (over 10% N base pairs or Q10 < 50%) paired-end reads were removed, which resulted in 560 Gb of high-quality Hi-C data. Hi-C data were mapped using BWA with the aln method. The uniquely mapped reads with map quality >20 were retained to perform assembly. Duplicate removal, sorting and quality assessment were performed with HiC-Pro (version 2.8.1) with the command ‘mapped_2hic_fragments.py -v -S -s 100 -l 1000 -a -f -r -o’. The Hi-C links were aggregated in 50-kb bins and normalized separately for intra- and intercontig contacts. Any two segments that showed inconsistent connection with the contigs were split into two fragments at the lowest coverage site. A total of 2,249 contact points with potential assembly error were detected and split for reassembling.

The corrected contigs were assembled into scaffolds by LACHESIS^54^. Adjacent contigs were linked together by filling the gap with ‘N’. A total of 47,477 contigs were anchored and oriented onto seven largest chromosome-scale super scaffolds (Supplementary Table 5). After gap filling with corrected PacBio reads and three rounds of manual adjustments, the seven pseudomolecules were evaluated using a genetic map derived from the Weining × Jingzhou cross and the BioNano reads (Supplementary Note).

The genetic map was developed using 295 F_2_ individuals from the Weining × Jingzhou cross, with the SNP markers generated by specific-locus amplified fragment sequencing^55^. A total of 35,905 SNPs, which were homozygous and polymorphic in the two parents, were identified. The SNPs with significant distortion (*χ*^2^ 1:2:1 test, *P* < 0.001), more than 40% missing data and less than 8× depth were discarded, which resulted in 3,691 high-quality SNPs used for linkage map construction. HighMap^56^ was employed to construct the linkage map with the setting MLOD > 3. In total, 2,662 SNPs were assigned to seven linkage groups with a total genetic distance of 843.8 cM. This genetic map was highly consistent with the seven chromosome-scale pseudomolecules, which were assigned to the 1R–7R chromosomes, respectively.

### Annotation and analysis of repeats

A combination of de novo and homolog search strategies was used to identify and annotate the repeat sequences in the Weining rye genome. RepeatScout, LTR-FINDER, MITE-Hunter and PILER-DF were used for ab initio prediction. The identified repeats were compared to those in the Repbase database (version 19.06), followed by classification into different repeat categories using the PASTEClassifier.py script included in REPET version 2.5. The CLARITE program was applied to perform TE annotation by homology^57^. Briefly, the Weining genome assembly was investigated for TEs using RepeatMasker with the TE database ClariTeRep. Next, the CLARITE module was used to correct raw similarity search results to solve the overlap and fragmentation problems of TE predictions and to reconstruct nested TEs. The families within each superfamily were classified using the 80–80–80 rule^57^. For LTR-RTs, the families were clustered based on their LTR sequences. The final set of repetitive sequences in the Weining rye genome was obtained by integrating the ab initio-predicted TEs and those identified by homology through RepeatMasker. Intact LTR-RTs were identified and analyzed using the ‘LTR_retriever’ pipeline (Supplementary Note).

### Annotation of protein-coding genes

De novo prediction, homology-based and transcriptome-based strategies were combined to identify and annotate protein-coding genes (Supplementary Fig. 3). To facilitate gene annotation, 25 transcriptomic datasets were generated for Weining rye by performing Illumina RNA-seq on leaf, stem, root and spike samples as well as on developing grain samples harvested at 10, 20, 30, 40 d after anthesis (Supplementary Table 11). The transcripts were assembled, followed by merging and removal of redundancy using HISAT (version 2.0.4) and StringTie (version 1.2.3). Concomitantly, two PacBio RNA-seq experiments were conducted for Weining rye with the Sequel platform using total RNA extracted from mixed organs or mixed grains (Supplementary Table 12). Two libraries with insert sizes ranging from 0.5 to 8 kb were constructed and sequenced, yielding 29 and 31 Gb of sequencing data, respectively. These data were processed using IsoSeq3. Circular consensus sequences were generated with the parameters ‘min_length 300, no_polish TRUE, min_passes 1, min_predicted_accuracy 0.8, max_length 15000’. The transcripts constructed from Illumina and PacBio transcriptome data were merged and aligned to the Weining genome assembly using BLAT (identity ≥95%, coverage ≥90%), and unigenes (chromosome loci) were identified using PASA (version 2.0.4). Afterward, the mapped reads were assembled into longer transcripts using Cufflinks software. TransDecoder was then applied to analyze gene structures.

All predicted gene structures were integrated into consensus gene models using EVidenceModeler (version 1.1.1). These gene models were filtered sequentially to identify reliable protein-coding genes by (1) removing the CDS with length less than 300 bp and (2) discarding the CDS that could not be translated because they lacked an open reading frame or had premature stop codons. A total of 86,991 protein-coding genes were thus generated, which were further classified into HC and low-confidence genes (Supplementary Fig. 3). The former category was supported by homology (identity ≥80% and coverage ≥50%, with the HC gene sets of Tu, Aet, Hv and Chinese Spring) or transcriptome data (TPM > 1) and lacked TE sequences, while the latter class was not supported by homology or transcriptome data, with 3.62% of the members showing similarities to TEs. The HC gene models were functionally annotated according to the best matches with proteins deposited in GO, KEGG, Swiss-Prot, TrEMBL and a non-redundant protein database using BLASTP (*E* value = 1 × 10^−5^).

### Phylogeny and divergence time analysis

OrthoMCL (version 1.1.4) was used to identify single-copy orthologous genes conserved in Weining rye and nine other grasses (Os, Bd, Hv, Aet, Tu, Ta, *Z. mays*, *S. bicolor* and *S. italica*). For Ta, the three subgenomes were analyzed separately. All-versus-all BLASTP (*E* value < 1 × 10^−5^) was performed, which led to the identification of 2,517 single-copy orthologous genes. MUSCLE was used to perform multiple alignment of deduced protein sequences, followed by construction of gene trees with BEAST version 2.5.1. The gene phylogenies were calibrated using a Bayesian relaxed clock, implemented in BEAST as previously reported^58^ with two priors: (1) the Bd stem node with a normal-distributed prior (44.4 ± 3.53 Ma) obtained from 17 fossil-calibrated analyses and (2) the Aet stem node with normally distributed calibration in the root of the tree (6.55 ± 0.22 Ma). Subsequently, DensiTree was used to generate a superimposed plot of ultrametric gene trees of the 2,517 orthologous genes. Genome divergence for each pair of diploid species or genomes was estimated based on the distribution of coalescence times of the 2,517 orthologous genes under the multispecies coalescent model^58^.

### Synteny analysis between Weining rye and rice or common wheat

To identify syntenic gene blocks between rye and rice, barley or common wheat subgenomes, all-against-all BLASTP (*E* value < 1 × 10^−5^, top five matches) was performed for the HC gene sets of each genome pair. Syntenic blocks were defined based on the presence of at least five synteny gene pairs using the MCScanX package with default settings. The adjacent blocks were merged, and large syntenic blocks, each with a size over 10 Mb, were selected. These large syntenic blocks were then used to deduce the chromosome evolutionary scenario of Weining rye as compared with that of rice and to investigate the syntenic relationships between Weining rye and common wheat.

### Analysis of gene duplication

The ‘duplicate_gene_classifier’ program implemented in the MCScanX package was employed to classify the HC genes located on chromosomes into four categories, whole-genome or segmental, tandem, proximal or dispersed duplications, based on all-versus-all local BLASTP (*E* value < 1 × 10^−5^, top five matches) within each species. Then the TrDGs were classified from dispersed duplications using the ‘DupGen_finder’ pipeline (https://github.com/qiao-xin/DupGen_finder). Barley was used as an outgroup to identify the intra-species collinear genes and interspecies collinear genes for Weining rye, Tu and Aet. The TrDG members located in the collinear syntenic regions were deduced to be the parental copies, whereas the TrDGs residing at alternative loci were considered to be transposed copies.

### Search for starch biosynthesis-related genes

The nucleotide sequences for common wheat SBRGs were retrieved from the Chinese Spring genome sequence (version 1.1). They were used to search the Weining genome assembly using BLASTN (*E* value < 1 × 10^−10^) to identify rye SBRG orthologs (identity ≥70% and coverage ≥60%). The normalized counts of SBRG expression were calculated using Illumina RNA-seq data from root, stem, leaf, spike and developing grain samples with TopHat and Cufflinks. The R package pheatmap was used to display the expression patterns of Weining rye SBRGs in different samples.

### Investigation of seed storage proteins

The SSP gene sequences of common wheat, Aet and Hv, including those encoding high- and low-molecular weight glutenin subunits, α-, γ-, ω- and δ-gliadins or γ-, B-, C- and D-hordeins, were employed to search the Weining genome assembly using BLASTN and BLASTP (*E* value < 10^−10^). Matched secalin gene sequences were manually annotated to separate intact genes from pseudogenes. To verify secalin gene sequences, the PacBio RNA-seq data from Weining rye developing grains (Supplementary Table 12) were searched to find the full-length transcripts of secalins using IsoCon. All matched circular consensus sequences were clustered and error corrected to obtain the final transcripts. These transcripts, together with the secalin genes identified above, were used to define each secalin locus. SDS–PAGE analysis of Weining rye SSPs was accomplished as described previously^33^.

To disentangle the evolutionary relationships of Triticeae SSPs, a phylogenetic tree was constructed using SSP sequences from Weining rye, Aet, Hv and common wheat. MUSCLE was used to align 93 SSP sequences (Supplementary Table 20); the phylogenetic tree was inferred with MEGA X using the maximum likelihood method and the JTT matrix-based model with 1,000 bootstraps. The phylogenetic tree was displayed and annotated with iTOL (https://itol.embl.de/). Microsynteny analysis of secalin loci was conducted using the module ‘jcvi.compara.synteny’ of MCscan (Python version) with the ‘--iter=1’ setting.

### Expression of heading date-related genes

Illumina RNA-seq was conducted for the leaf samples collected from Weining and Jingzhou plants at 4, 7 and 10 DAS, with three biological replicates used per genotype per time point (Supplementary Table 11). The resultant transcriptomic data allowed identification and quantification of the genes related to heading date. The expression patterns of these genes (Extended Data Fig. 8) were displayed using the R package pheatmap.

For studying the expression patterns of *ScFT1*, *ScFT2* and *ScPpd1* in Weining and Jingzhou plants, RT–qPCR assays were performed with the cDNA that was reverse-transcribed from the total RNA extracted from leaf samples collected at different DAS time points with gene-specific primer sets (Supplementary Table 23). For each gene, three biological replicates were analyzed per genotype per DAS time point using RT–qPCR^42^. A rye actin gene (*ScWN1R01G374800*) was amplified as an internal control.

### Investigation of ScFT protein expression and phosphorylation

A polyclonal rabbit antibody specific for ScFT was raised using the peptide QLGRQTVYAPGWRQ, conserved in ScFT1 and ScFT2 (Supplementary Table 24), as described previously^59^. This antibody was employed to compare ScFT protein accumulation levels in Weining and Jingzhou plants at 4, 7 and 10 DAS by immunoblotting. In brief, total leaf proteins (20 μg per sample) were separated using 12% SDS–PAGE, followed by transfer to a PVDF membrane. Subsequently, the membrane was treated with the anti-ScFT antibody (1:2,000 dilution) and then the secondary antibody goat anti-rabbit IgG H&L (IRDye 800CW, 1:5,000 dilution, Abcam), and reaction signals were recorded using the LI-COR 2800. Detection of the HSP90 protein served as a loading control as described previously^60^.

Phos-tag SDS–PAGE^41^ was employed to analyze the phosphorylation of the ScFT protein, which was conducted according to the Phos-tag Acrylamide protocol handbook (Wako Laboratory Chemicals, Phos-tag Acrylamide, AAL-107). Briefly, total proteins were extracted from the leaves of 7-d-old plants using lysis buffer (10 mM Tris-Cl pH 7.5, 150 mM NaCl, 0.5% NP-40, 1 mM phenylmethyl sulfonyl fluoride) containing 1× protease inhibitor cocktail (Roche Diagnostics, 11836170001) and 1× phosphatase inhibitor cocktail (Roche Diagnostics, 4906837001), followed by centrifugation for 15 min (12,000 r.p.m.) at 4 °C. The supernatant (containing ~20 μg protein) was treated with or without Lambda Protein Phosphatase (New England Biolabs, P0753L) for 30 min at 30 °C and then mixed with an equal volume of 2× SDS sample buffer. After boiling for 5 min, the proteins were separated using 12% Phos-tag SDS–PAGE and detected by immunoblotting with the anti-ScFT antibody as described above.

### Selection sweep analysis

A genome-wide genotyping-by-sequencing dataset, previously published for 101 accessions of domesticated rye and wild *Secale* forms^5^, was employed for SNP calling. This germplasm panel included 81 rye accessions, five accessions of *S. vavilovii*, 11 accessions of *S. strictum* and four accessions of *S. sylvestre*. The variants were identified using BWA with default parameters and SAMtools with the parameter ‘-R -d 1000000 -t DP,AD -Q 20 -q 30 -Bug’. Only the biallelic SNPs with quality scores greater than 50, a minimum allele frequency >0.05, missing data <40% and read depth >4 were retained. This resulted in a total of 127,826 high-quality SNPs, of which 124,472 (97.38%) were assigned to the seven chromosomes of the Weining assembly. These SNPs were distributed mainly in the distal chromosomal regions (Supplementary Fig. 6a). The 127,826 SNPs were also annotated using SnpEff (version 4.2) with Weining gene models, which allowed SNPs to be assigned to intergenic or different genic regions (Supplementary Fig. 6b).

The selective sweeps potentially related to rye domestication were investigated using DRI (*π*_*S. vavilovii*_ × *π*_rye_^−1^), *F*_ST_ and XP-CLR, following methods in previous studies^61–63^. The *π* and *F*_*ST*_ values were calculated in 5-Mb windows with 1-Mb steps using VCFtools. The mean and median numbers of SNPs in the 5-Mb sliding windows were 77.8 and 54, respectively (Supplementary Fig. 6c). XP-CLR scores between two populations were obtained using a window size of 0.1 cM, a grid size of 100 kb and a maximum of 200 SNPs within a window; for the SNPs with a linkage disequilibrium *r*^2^ over 0.95, only one of the SNPs was used. Genetic positions of the SNPs were determined using the linkage map generated with the F_2_ population of the Weining × Jingzhou cross (Supplementary Data 1) by assuming uniform recombination between markers. Next, the mean XP-CLR likelihood score was calculated in 5-Mb sliding windows with 1-Mb steps across the genome. During scanning for selection sweeps, only the windows with five or more SNPs were used, with the top 5% outliers of the whole-genome rank chosen as initial selection sweep signals. The signals that were ≤1 Mb apart were merged together as a single selection sweep. Only the signals containing at least ten SNPs in the corresponding genomic regions were kept as final sets of putative selection sweeps (Supplementary Data 5). To investigate the genes located in the putative selection sweeps, their orthologs in rice or barley were identified using MCScanX as described above. The functional information for syntenic rice genes was obtained from funRiceGenes (https://funricegenes.github.io/). Analysis of *ScID1* is described in the Supplementary Note.

### Statistical analysis

Spearman’s rank correlation coefficient test statistic was performed using the ‘cor.test’ function in R (parameters, method = ‘spearman’; exact = TRUE). Two-tailed Student’s *t*-tests were executed using ‘t.test’ in R (parameters, alternative = ‘two.sided’; paired = FALSE).

### Reporting Summary

Further information on research design is available in the Nature Research Reporting Summary linked to this article.

## Online content

Any methods, additional references, Nature Research reporting summaries, source data, extended data, supplementary information, acknowledgements, peer review information; details of author contributions and competing interests; and statements of data and code availability are available at 10.1038/s41588-021-00808-z.

## Supplementary information

Supplementary Information Supplementary Note, Figs. 1–6 and Tables 1–24

Reporting Summary

Supplementary Data 1 Construction of the genetic map and detection of QTL for heading date.

Supplementary Data 2 Analysis of expression levels of the genes related to starch biosynthesis.

Supplementary Data 3 A list of the 1,989 predicted DRA genes of Weining rye.

Supplementary Data 4 Expression levels of the genes related to heading date in leaf tissues of Weining and Jingzhou rye plants.

Supplementary Data 5 Summary of selection sweeps related to rye domestication as computed using three methods.

## Data Availability

The Weining rye genome assembly was deposited in NCBI GenBank under the accession number JADQCU000000000. The raw sequencing data were deposited in the NCBI Sequence Read Archive under the BioProject accession numbers PRJNA680931, PRJNA680499 and PRJNA679094. The assembly and annotation data were also submitted to the Chinese National Genomics Data Center (https://bigd.big.ac.cn/) under the accession number GWHASIY00000000. The Weining rye genome assembly and annotation are additionally available from the Triticeae Multi-omics Center (http://wheatomics.sdau.edu.cn/). Source data are provided with this paper.

## Source data

Source Data Fig. 6 Unprocessed western blots for Fig. 6.
Source Data Extended Data Fig. 5 Unprocessed gels for Extended Data Fig. 5.
Source Data Extended Data Fig. 9 Unprocessed western blots for Extended Data Fig. 9.
