# Supplementary material for: A high-quality genome assembly highlights rye genomic characteristics and agronomically important genes
Source: Nat Genet. 2021 Mar 18;53(4):574–84. doi: 10.1038/s41588-021-00808-z (PMC8035075; doi:10.1038/s41588-021-00808-z)
Supplement: Supplementary file 1 — Supplementary Note, Figs. 1–6 and Tables 1–24 [file 41588_2021_808_MOESM1_ESM.pdf]

nature portfolio
https://doi.org/10.1038/s41588-021-00808-z

Supplementary information

A high-quality genome assembly highlights rye genomic characteristics and agronomically important genes

In the format provided by the authors and unedited

Supplementary Note

Generation of BioNano optical molecules. For generating BioNano optical molecules, Weining rye high-molecular-weight DNA was treated with the nicking endonuclease *BspQ*I and labeled by fluorescent-dUTP using the IrysPrep Reagent Kit according to manufacturer's instructions. The labeled molecules were imaged and analyzed using the BioNano Irys system. A total of fifteen flow cells were run, the molecules, with a minimum length of 100 kb and a SNR (signal-to-noise ratio) value higher than 3.5, were retained. The final set of cleaned data were 779.55 Gb including 3,571,570 molecules, with the N50 being 239.8 kb.

Evaluation of genome assembly using BioNano reads. In the evaluation using BioNano reads, the optical molecules were mapped to the seven pseudomolecules using the RefAligner software in the IrysView package with the parameter '-nosplit 2 -BestRef 1 -biaswt 0 -Mfast 0 -FP 1.5 -sf 0.2 -sd 0.0 -A 5 -outlier 1e-4 -endoutlier 1e-3 -S -1000 -sr 0.04 -resbias 5 64 -maxmem 36 -M 3 -minlen 150 -minsites 12 -sort-sizedec -subset 1 2000000 -T 1e-8 -maxthreads 12 -hashgen 5 3 2.4 1.5 0.05 5.0 1 1 2 -hash -hashdelta 10 -hashmaxmem 36 -insertThreads 8 -stdout -stderr'. Approximately 96.02% of the seven pseudomolecules were covered by BioNano molecules.

Assessment of genome assembly. A high-density genetic map, developed using a cross between two European winter rye cultivars (Lo7 and Lo225)[1], was used to assess the accuracy of the Weining rye genome assembly. The 87,820 marker sequences in the genetic map were searched against Weining genome assembly by BLASTN using an identity cutoff of 85%, with 79,073 markers anchored onto the seven Weining rye chromosomes and 6,417 markers mapped to the unanchored scaffolds. A total of 25,286 markers, which exhibited unique hit in Weining genome assembly with identity values over 99%, were used to plot the genetic map distance versus the physical position of Weining rye chromosome assemblies (Supplementary Fig. 5). The Spearman's rank correlation coefficient between the two maps, calculated using R, was 0.99 ($P < 2.2 \times 10^{-16}$). Moreover, 194,804 Roche/454 reads of Lo7 with an average length 400 bp were downloaded from a previous study[1]. Among them, 169,717 had no Ns in their sequences, and used to BLASTN against the Weining genome assembly. A total of 165,392 (97.45%) reads could be aligned to Weining

assembly with an average sequence identity of 97.71% and a mean sequence coverage of 97.27%.

To check the nucleotide accuracy of Weining genome assembly, the 13 Illumina pair-end library reads (Supplementary Table 3) were aligned to the Weining assembly using BWA. Alignments were sorted using SAMtools, and the variants were called by GATK HaplotypeCaller module with the parameter "--minimum-mapping-quality 40 --min-base-quality-score 20 --native-pair-hmm-threads 50". The identified SNPs and indels were filtered using VCFtools with the command "--remove-filtered-all --min-alleles 2 --max-alleles 2 --min-meanDP 5 --max-meanDP 300 --minQ 40". The homozygous SNPs and indels identified were used to calculate nucleotide base accuracy rate of the assembly.

LTR_retriever[2] was used to calculate the LAI score of Weining rye genome assembly with 3 Mb window size and 300 kb sliding step. For comparison, the same protocol was applied to calculate the LAI values of the genomes of rice, *T. urartu*, *Ae. tauschii*, *H. vulgare* and the three subgenomes of common wheat (Supplementary Fig. 6). To evaluate the completeness of gene annotations, the 1,440 conserved protein models in the BUSCO embryophyta_odb9 dataset[3] were searched against Weining genome assembly.

Detection and analysis of intact LTR-RTs. Intact LTR-RTs were identified using LTR_retriver. LTRharvest and LTR_FINDER_parallel (https://github.com/oushujun/LTR_FINDER_parallel) were used to identify full length LTR-RT candidates. LTRharvest was run with the following setting: "-seed 20 -minlenltr 100 -maxlenltr 7000 -similar 85 -mintsd 4 -maxtsd 6 -motif TGCA -motifmis 1 -vic 10 -seqids yes"; LTR_FINDER_parallel was executed with default parameters. The results were merged together in LTRharvest standard output format, followed by analysis in the LTR_retriver pipline with default parameters. A nucleotide substitution rate of 1.3×10^{-8} mutations per site per year was used to estimate the insertion time of intact LTR-RTs with the formula of $T = K/2\mu$ as described previously[4,5], where K is the divergence rate of 5′-LTR and 3′-LTR estimated by the Jukes-Cantor model.

Identification of centromeric regions. Previous genome analysis of common wheat showed that the *Cereba* and *Quinta* families of Gypsy LTRs are concentrated in the centromeric regions, and can be used to identify centromeres[6]. Thus to identify the

centromeric regions of Weining rye chromosomes, the proportion of *Cereba* LTR-TRs along each chromosome was calculated using a sliding window of 1 Mb size with a step of 100 kb (i.e., total length of *Cereba* / the window size), which resulted in a total of 23,903 *Cereba* containing windows for the seven chromosomes. The 99th-percentile value 0.215 of the null distribution of these windows was used as a cutoff for estimating the centromeric region of each Weining rye chromosome.

Identification of non-coding RNA genes. The tRNA genes were identified using tRNAscan-SE v2.0 (http://lowelab.ucsc.edu/tRNAscan-SE/). The miRNAs were found by homology searching (one mismatch allowed) against miRBase (Release 22). The secondary structures of putative miRNA sequences were predicted by miRDeep2, with the miRNAs having typical hairpin structures considered to be reliable candidates. Other non-coding RNAs were predicted with the software Infernal v1.1.2 (http://eddylab.org/infernal/) using default parameters.

Identification of transcription factor genes. The iTAK program[7] was applied to identify the TF genes of Weining rye based on homology search against the known plant transcription factor database integrated in the program, with the search results classified into different TF families. To compare the TF families among different grass species, iTAK was also applied to compute the TF genes in TaA, TaB, TaD, DUWA, DUWB, WEWA, WEWB, Tu, Aet, Bd, Hv and Os.

Annotation of disease resistance associated genes. RGAugury pipeline was used to screen the HC genes of Weining rye and other grasses in order to identify the DRA genes[4]. The pipeline first identified the conserved domains or motifs commonly present in the DRA proteins, including nucleotide binding site (NB-ARC), leucine rich repeat (LRR), trans-membrane (TM), serine/threonine and tyrosine kinase (STTK), lysine motif (LysM), coiled-coil (CC), and Toll/ Interleukin-1 receptor (TIR). Then the candidates were identified and classified into one of the four known major families: NBS-encoding, trans-membrane and coiled-coil (TM-CC), receptor like kinase (RLK), and receptor like protein (RLP). The distribution of DRA genes along Weining rye chromosomes was calculated using a 10 Mb non-overlap sliding window.

QTL mapping. The heading date was scored for the 295 F2 plants derived from the Weining × Jingzhou cross. The resultant phenotypic data, together with the genetic map (Extended Data Fig. 3), were used for QTL mapping with the composite interval

mapping method implemented in R/qtl[8], with a 10 cM scan window and the covariates of 3 markers. LOD threshold was set by the top 5% quantile based on 1000 permutation tests, and a 1.5 LOD-drop support interval was used for each QTL. The addictive effect and the variation explained by each QTL were determined using the R function lm. The most significant SNPs were used to estimate QTL effect size.

Analysis of *ScID1*. Comparison of syntenic genomic regions carrying *ID1* genes in Sc, Tu, Aet, and Ta was conducted using the module jcvi.compara.synteny of MCscan (Python version) with "--iter=1"setting. The expression of *ScID1* at 3 DAS time points was assayed by qRT-PCR, with a primer set recognizing both *ScID1.1* and *ScID1.2* (Supplementary Table 23). *ScID1* genotyping of individual F2 plants of Weining × Jingzhou cross was accomplished using a SLAF sequencing generated SNP marker that was located nearest to the *ScID1* locus. With this marker, three *ScID1* genotypes, homozygous Weining or Jingzhou *ScID1* (designated as WN/WN and JZ/JZ, respectively) and heterozygous *ScID1* (WN/JZ), were distinguished, and the number of F2 plants for the three genotypes was 66, 64, and 161, respectively. Statistical analysis of heading date data of the three genotypes was executed in R using two-tailed *t*-test. Multiple amino acid sequence comparison of the ID1 proteins from Os, Zm, Sc, Hv, Tu, Aet, and Ta was carried out with Clustal Omega (https://www.ebi.ac.uk/Tools/msa/clustalo/). Phylogenetic analysis of ID1 proteins was performed using MEGA X (www.megasoftware.net/), with the phylogenetic tree constructed using the Maximum Likelihood method and a JTT matrix-based model.

Analysis of ScFT2 phosphorylation in tobacco. Potential serine, threonine or tyrosine phosphorylation sites in ScFT2 were predicted using NetPhos 3.1 Server (http://www.cbs.dtu.dk/services/NetPhos/). The residues with a phosphorylation potential score of above 0.5 (S38, S76, S110, and T132, Supplementary Fig. 4) were considered for further investigations. For identifying potential phosphorylation sites conserved among monocot and dicot plants, the FT1 and FT2 proteins from Sc, Bd, Os, TaA, TaB, TaD, Aet, and Hv, together with the FT1 protein of *Arabidopsis thaliana*, were subjected to multiple amino acid sequence comparison using the Clustal Omega (https://www.ebi.ac.uk/Tools/msa/clustalo/), which showed that S76 and T132 were strictly conserved among the 17 compared FT proteins

(Supplementary Fig. 4). These two residues were thus mutated to create a series of de-phosphomimic (S76A, T132A, and S76A/T132A) and phosphomimic (S76D, T132D, and S76D/T132D) mutants, followed by expression in tobacco (*N. benthamiana*) using a PVX vector as described previously[9]. Briefly, the nucleotide sequences, designed to express MYC-tagged wild type (WT) ScFT2 and six derivative mutants, were synthesized commercially (Beijing SYKM Gene Biotechnology Co., Ltd., Beijing, China), and then cloned into the PVX vector through infusion cloning (http://bioinfo.clontech.com/infusion/) with the primers containing *Cla* I and *Sal* I restriction enzyme digestion sites (Supplementary Table 23). The resultant recombinant PVXs were each introduced into the leaves of *N. benthamiana* seedlings via agroinfiltration. PVX:GFP, expressing free GFP, was used as a control. The infiltrated plants were cultured in a growth room set at 23 °C and with a 16 h light/ 8 h dark photoperiod. They were checked for successful viral infection at 10 days post infiltration (dpi) by detecting PVX genomic RNAs using RT-PCR[9]. The number of flowering plants was counted at 25 dpi, with representative images taken at the same time. The accumulation of WT and mutant ScFT2 proteins in the tobacco plants was also analyzed at this time by immunoblotting with an anti-MYC primary antibody (Sigma-Aldrich, C3956). We noted that the MYC-tagged ScFT2 accumulated in tobacco had a molecular mass of ~23 kDa (Fig. 6g), which was smaller than the ScFT protein (~29 kDa, Fig. 6d) detected in rye. This difference indicated that the phosphomodifications of ScFT protein in tobacco and rye differed to some extent, which requires further study to be understood. Three independent experiments were performed, with 3 to 8 plants infiltrated per PVX construct per experiment. The percentages of flowering plants determined for the different PVX constructs were presented as means ± SE, which were statistically compared to the control PVX:GFP by a two-tailed Student's *t*-test using *t.test* in R.

References

1. Bauer, E. et al. Towards a whole-genome sequence for rye (*Secale cereale* L.). *Plant J.* **89**, 853-869 (2017).
2. Ou, S., Chen, J. & Jiang, N. Assessing genome assembly quality using the LTR Assembly Index (LAI). *Nucleic Acids Res.* **46**, e126 (2018).

3. Simao, F.A., Waterhouse, R.M., Ioannidis, P., Kriventseva, E.V. & Zdobnov, E.M. BUSCO: assessing genome assembly and annotation completeness with single-copy orthologs. *Bioinformatics* **31**, 3210-3212 (2015).

4. Luo, M.C. et al. Genome sequence of the progenitor of the wheat D genome *Aegilops tauschii*. *Nature* **551**, 498-502 (2017).

5. Avni, R. et al. Wild emmer wheat genome architecture and diversity elucidate wheat evolution and domestication. *Science* **357,** 93-97 (2017).

6. International Wheat Genome Sequencing Consortium (IWGSC). et al. Shifting the limits in wheat research and breeding using a fully annotated reference genome. *Science* **361,** pii:eaar7191 (2018).

7. Zheng, Y. et al. iTAK: A program for genome-wide prediction and classification of plant transcription factors, transcriptional regulators, and protein kinases. *Mol. Plant* **9**, 1667-1670 (2016).

8. Arends, D., Prins, P., Jansen, R.C. & Broman, K.W. R/qtl: high-throughput multiple QTL mapping. *Bioinformatics* **26,** 2990-2 (2010).

9. Qin, C. et al. A virus-induced assay for functional dissection and analysis of monocot and dicot flowering time genes. *Plant Physiol.* **174,** 875-885 (2017).

Supplementary Figures

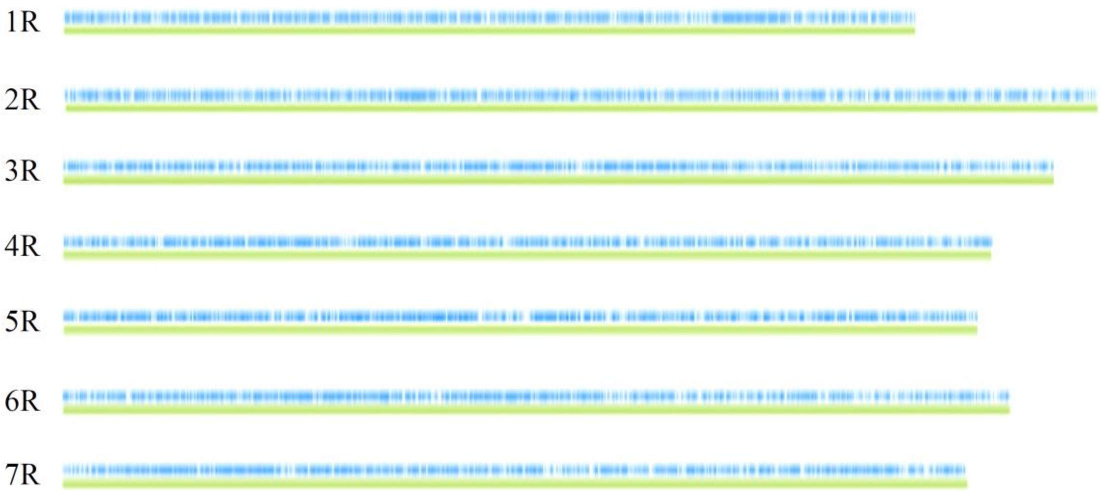

Supplementary Fig. 1. Alignment between BioNano reads (top) and the seven assembled chromosomes (1R-7R) of Weining rye (below). Approximately 96.02% of the physical map was covered with BioNano molecules.

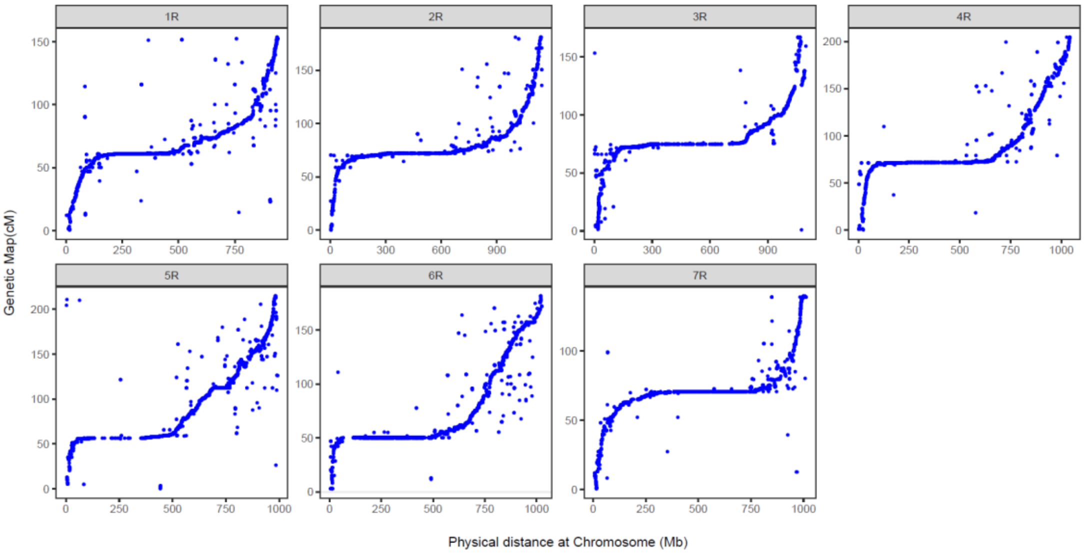

Supplementary Fig. 2. Alignment between the seven assembled chromosomes of Weining rye and the seven rye linkage groups developed using Lo7 x Lo255 RIL population. The DNA markers used for constructing the plot were 25,286, which exhibited unique hit in Weining genome assembly with identity values over 99%.

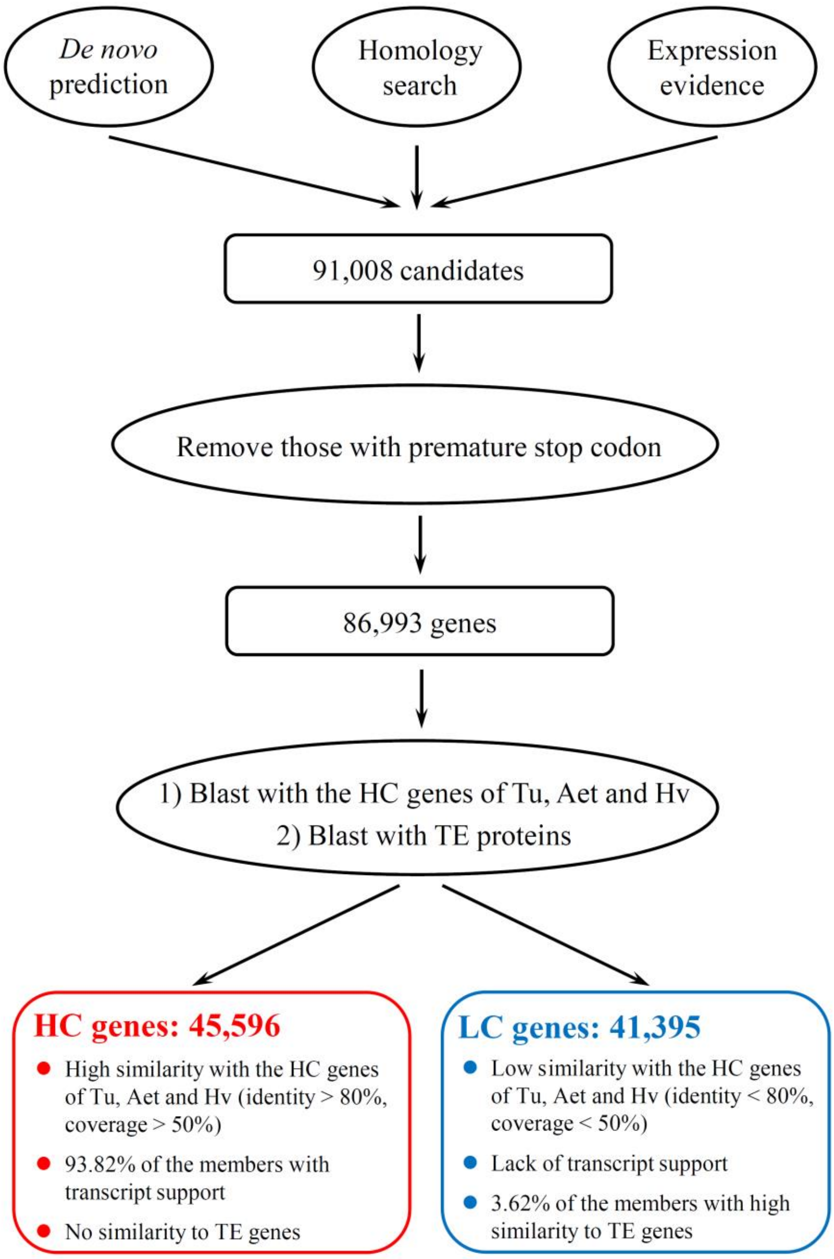

Supplementary Fig. 3. Gene annotation pipeline for Weining rye genome assembly. Three different approaches, including Genscan, Augustus (version 2.4) and GlimmerHMM (version 3.0.4), were employed for *de novo* prediction with default parameters. In the homology-based approach, the HC gene models of previously sequenced Triticeae species, including Tu (*T. urartu*), Aet (*Ae. tauschii*), Hv (*H. vulgare*), and Ta (*T. aestivum*), were used to annotate Weining rye genes with the programs GeneWise and GeMoMa. Expression evidence was obtained through analysis of transcriptome sequencing data (Methods).

a

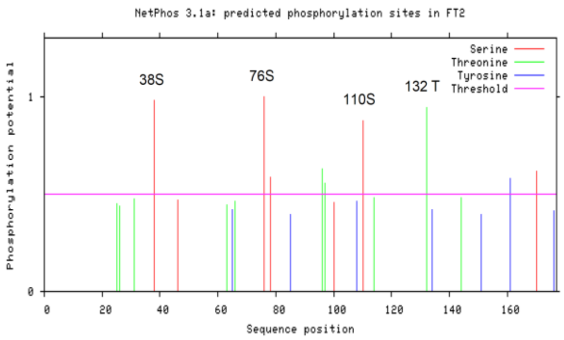

b

FT2 (177 amino acids; Score > 0.5) Netphos-3.1b prediction results					
**Sequence**	**Residue**	**Context**	**Score**	**Kinase**	**Answer**
ScFT2	76 S	PDAPSPSDP	0.996	unsp	YES
	76 S	PDAPSPSDP	0.542	cdk5	YES
	76 S	PDAPSPSDP	0.522	GSK3	YES
	132 T	LGRQTVYAP	0.940	unsp	YES
	132 T	LGRQTVYAP	0.662	PKC	YES

c

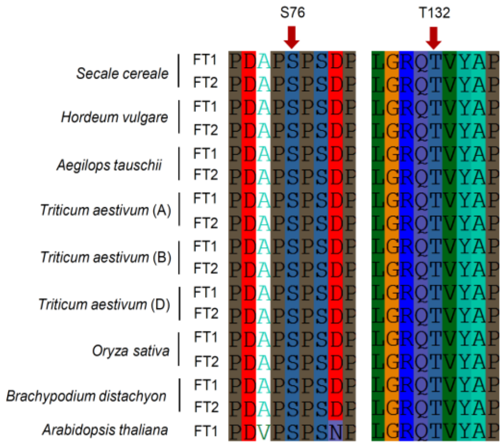

Supplementary Fig. 4. Prediction of potential phosphorylation sites in ScFT2. (**a, b**) The deduced protein sequence of ScFT2 was subjected to prediction using the software NetPhos 3.1 Server (http://www.cbs.dtu.dk/services/NetPhos/) (**a**). The residues with a phosphorylation potential score of above 0.5 (S38, S76, S110, and T132) are listed (**b**). (**c**) Multiple alignment of the amino acid sequences of the FT1 and FT2 proteins from rye, barley (*Hordeum vulgare*), *Aegilops tauschii*, the A, B and D subgenomes of common wheat (*Triticum aestivum*), rice (*Oryza sativa*), *Brachypodium distachyon*, and *Arabidopsis thaliana*. The alignment was generated using the Clustal Omega software (https://www.ebi.ac.uk/Tools/msa/clustalo/), which showed that S76 and T132 were strictly conserved among the 17 compared FT proteins. Only the relevant portion of the alignment is shown. The amino acid sequences of the 17 FT proteins are provided in Supplementary Table 24.

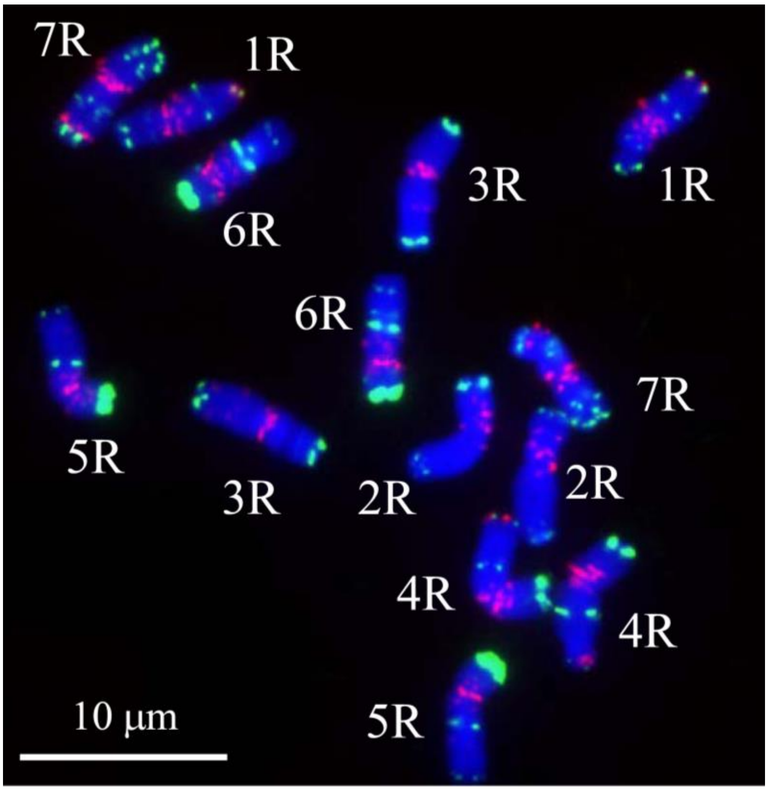

Supplementary Fig. 5. The seven pairs of chromosomes (1R to 7R) of Weining rye. Metaphase chromosomes in the mitotic root tip cells were hybridized with two fluorescently labeled probes, pSC119.2 (green) and $(AAC)_5$ (red), which allowed identification of the seven pairs of rye chromosomes. The data shown were reproducible in three independent experiments.

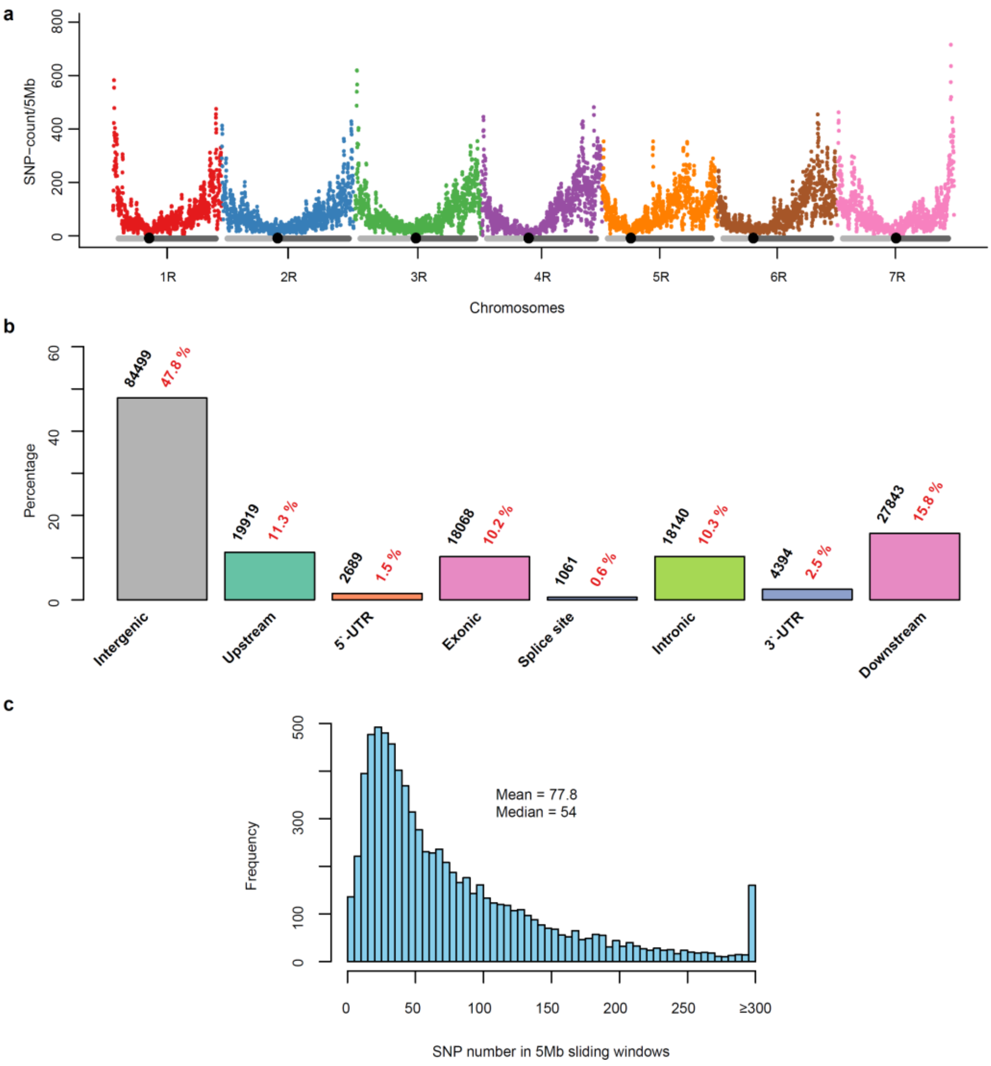

Supplementary Fig. 6. Distribution patterns of the SNPs called using the genotyping-by-sequencing data of domesticated rye (81 accessions) and three wild *Secale* species including *S. vavilovii* (5), *S. strictum* (11), and *S. sylvestre* (4) with Weining genome assembly as reference. (**a**) Distribution patterns of 124,472 chromosomally assigned SNPs on the 1R to 7R chromosomes of Weining rye. (**b**) Distribution of 127,826 SNPs in the intergenic or different genic regions of Weining rye as annotated using the SnpEff software (http://snpeff.sourceforge.net/ features.html). (**c**) Distribution pattern of the 5 Mb sliding windows containing different numbers of SNPs.

Supplementary Tables

Supplementary Table 1. Summary of sequencing platforms used and the data generated in constructing Weining rye genome sequence

**Platform**	**Libraries sequenced**	**Data size**	**Depth**
Illumina	13	430 Gb	54 ×
PacBio	120	497 Gb	62 ×
Hi-C	6	560 Gb	70 ×
BioNano	15	779 Gb	97 ×

Supplementary Table 2. Summary of PacBio reads generated in this study

Length (bp)	Number	Total length (bp)	Average length (bp)
0 - 2000	9,186,702	11,237,416,294	1,223
2000 - 4000	10,913,437	32,294,858,639	2,959
4000 - 6000	8,762,119	43,565,621,971	4,972
6000 - 8000	8,393,934	58,673,408,931	6,990
8000 - 10000	6,640,491	59,442,959,031	8,952
10000 - 12000	5,325,504	58,431,134,186	10,972
12000 - 14000	4,201,538	54,392,496,752	12,946
14000 - 16000	2,946,672	44,015,688,923	14,937
16000 - 18000	2,018,402	34,181,945,704	16,935
> 18000	4,353,731	100,781,681,018	23,148
**Total**	**62,742,530**	**497,017,211,449**	**7,922**
Reads N50 (bp)	11,484	-	-
Max length (bp)	180575	-	-

Supplementary Table 3. Summary of Illumina Hiseq X Ten data

Library	Data (Gb)	Depth (×)	Q20 (%)	Q30 (%)
270bp_1	32.67	4.08	95.03	88.56
270bp_2	34.52	4.32	95.14	88.75
270bp_3	35.79	4.47	95.13	88.73
270bp_4	33.24	4.16	95.02	88.52
270bp_5	31.38	3.92	95.18	88.53
270bp_6	36.36	4.55	95.36	88.93
270bp_7	37.64	4.71	94.93	88.12
270bp_8	32.60	4.08	95.06	88.34
270bp_9	35.16	4.40	96.50	91.43
270bp_10	37.76	4.72	96.52	91.43
270bp_11	35.39	4.42	96.51	91.45
270bp_12	31.00	3.88	96.54	91.50
270bp_13	16.44	2.06	96.57	91.61
Total	429.95	53.74	-	-

Supplementary Table 4. Summary of Hi-C data and mapping

**Library**[1]		**Total read pairs**	**Mapped reads**	**Unique mapped read pairs**	**Valid interaction pairs**	**Dangling end pairs**	**Re-ligation pairs**	**Self-cycle pairs**	**Dumped pairs**
H01	Size (bp)	268,366,265	485,799,666	68,204,581	44,632,071	17,367,681	950,051	543,665	4,711,113
	Ratio (%)	100	90.51	25.41	65.44	25.46	1.39	0.8	6.91
H02	Size (bp)	314,244,148	572,100,640	82,794,586	54,169,599	20,781,274	1,141,282	718,454	5,983,977
	Ratio (%)	100	91.03	26.35	65.43	25.1	1.38	0.87	7.23
H03	Size (bp)	341,464,950	622,771,703	90,309,721	60,171,383	21,614,830	1,234,076	746,632	6,542,800
	Ratio (%)	100	91.19	26.45	66.63	23.93	1.37	0.83	7.24
H04	Size (bp)	318,374,313	578,562,280	81,816,807	51,481,494	23,067,405	1,160,328	756,034	5,351,546
	Ratio (%)	100	90.86	25.7	62.92	28.19	1.42	0.92	6.54
H05	Size (bp)	277,499,548	499,896,565	69,843,529	43,739,691	19,814,454	1,008,909	592,303	4,688,172
	Ratio (%)	100	90.07	25.17	62.63	28.37	1.44	0.85	6.71
H06	Size (bp)	349,117,671	608,189,002	84,050,346	74,262,643	7,900,708	695,877	118,145	1,072,973
	Ratio (%)	100	87.1	24.08	88.35	9.4	0.83	0.14	1.28

[1]Five parallel Hi-C libraries (H01-H05) were constructed with the restriction enzyme *Dpn*I (5'-GA^{m6}↓TC3'), with the H06 library developed using the restriction enzyme *Hind*III (5'-A↓AGCTT-3').

Supplementary Table 5. Seven largest chromosome scale super scaffolds ordered and orientated using Hi-C data

Super scaffold	Number of contigs ordered and orientated	
	**Contig number**	**Total length (bp)**
Lachesis Group0 (Chr4)	9,328	1,075,009,153
Lachesis Group1 (Chr7)	8,181	1,036,064,969
Lachesis Group2 (Chr2)	11,345	1,172,237,068
Lachesis Group3 (Chr3)	8,673	1,121,865,493
Lachesis Group4 (Chr6)	10,013	1,055,758,236
Lachesis Group5 (Chr1)	9,035	964,780,726
Lachesis Group6 (Chr5)	9,344	1,027,035,830
Total clustered	65,919	745,2751,475
Ratio (%)	68.40	94.38
Total ordered and oriented	47,477	725,0197,380
Ratio (%)	72.02	97.28

Supplementary Table 6. Summary of BioNano data

BioNano high-quality data	
Filtered	100 kb
Enzyme	*BspQ*I
N mols	3,571,570
Total length (> 100 kb, ~ 97 ×)	779 Gb
Average length (> 100 kb)	218 kb
Total length (> 150 kb, ~75.8 ×)	607 Gb
Average length (> 150 kb)	280 kb
Mol N50 (kb)	239.8
Lab (/100kb)	11.0

The data were generated using the enzyme *BspQ*I on the BioNano Irys system. The filtered high quality data represent an average coverage of 97 × of the estimated Weining genome size (7.86 Gb).

Supplementary Table 7. Comparison of the sizes of assembled chromosomes of Weining rye and five other Triticeae species[1]

	**Sc**	**Tu**	**Aet**	**Hv**	**WEWA**	**WEWB**	**TaA**	**TaB**	**TaD**
Chromosome 1	0.94097 (1R)[2]	0.58	0.50	0.56	0.59	0.69	0.59	0.69	0.50
Chromosome 2	1.15054 (2R)	0.75	0.65	0.77	0.78	0.80	0.78	0.80	0.65
Chromosome 3	1.09484 (3R)	0.75	0.63	0.70	0.75	0.84	0.75	0.83	0.62
Chromosome 4	1.04378 (4R)	0.62	0.53	0.65	0.73	0.67	0.74	0.67	0.51
Chromosome 5	0.99891 (5R)	0.66	0.58	0.67	0.70	0.71	0.71	0.71	0.57
Chromosome 6	1.03447 (6R)	0.58	0.50	0.58	0.62	0.70	0.62	0.72	0.47
Chromosome 7	1.00967 (7R)	0.72	0.64	0.66	0.73	0.76	0.74	0.75	0.64

[1]Sc, *Secale cereale* (Weining); Tu, *T. urartu* (G1812); Aet, *Ae. tauschii* (AL8/78); Hv, *Hordeum vulgare* (Morex); WEWA and WEWB, the A and B subgenomes of wild emmer wheat (*T. turgidum* ssp. *dicoccoides*, Zavitan); TaA, TaB and TaD, the A, B and D subgenomes of common wheat (*T. aestivum*, Chinese Spring).

[2]For simplicity, only the chromosome sizes of Sc (1R to 7R) are shown with five digits after decimal.

Supplementary Table 8. Assessing genome assembly using WGS Hiseq X Ten reads

**Item**	**Statistics**
Total reads	2,775,853,179
Mapped reads	2,769,537,530 (99.77%)
Paired in sequencing	2,759,875,804
Properly paired	2,671,730,270 (96.81%)

Supplementary Table 9. Estimation of nucleotide accuracy in Weining genome assembly and the heterozygosity rate of Weining rye

**Nucleotide accuracy of Weining genome assembly**	
Number of homozygous SNPs	242,455
Number of homozygous InDels	218,570
Estimated accuracy (versus the assembled genome size 7,737,590,180 bp)	99.99404%

**Heterozygosity of Weining rye**	
Number of heterozygous SNPs	19,215,912
Number of heterozygous InDels	1,530,913
Estimated heterozygosity (versus the assembled genome size 7,737,590,180 bp)	0.26%

Supplementary Table 10. Assessment of completeness of the HC gene models of Weining genome assembly using BUSCO dataset (1,440 genes)

**Item**	**Number**
Number of full length BUSCO genes found	1,370 (1,024 single copy plus 346 duplicated copy)
Number of fragmented BUSCO gene found	23
Total number of BUSCO genes found	1,393
Percentage of BUSCO genes identified	96.74%

Supplementary Table 11. Characteristics of the Hiseq transcriptomes sequenced in this study

Target gene	Genotype	Tissue	Treatment/stage	Biological repeat	Sample ID	Clean read[1]	Mapping ratio (%)[1]
Genes acting in vegetative and reproductive organs	Weining	Root	Normal condition	4	R3-root	22241342	72.35
		Stem	Normal condition	4	R3-stem	23786069	82.93
		Leaf	Normal condition	4	R3-leaf	23171266	77.40
		Spike	Normal condition	4	R3-spiko	23124603	84.30
		Seed	10 days after anthesis	4	10d	29351768	86.35
		Seed	20 days after anthesis	4	20d	29335815	81.61
		Seed	30 days after anthesis	4	30d	27728075	81.28
		Seed	40 days after anthesis	4	40d	22052264	84.00
Genes acting in cold response	Weining	Root	No freezing (CK)	1	R3-0h-G	26102733	84.72
		Root	1 h after freezing (at -10 ℃)	1	R3-1h-G	24864187	84.49
		Root	4 h after freezing (at -10 ℃)	1	R3-4h-G	21813051	81.95
		Root	8 h after freezing (at -10 ℃)	1	R3-8h-G	22765797	82.15
		Leaf	No freezing (CK)	1	R3-0h-Y	26518627	84.67
		Leaf	1 hours after freezing (at -10 ℃)	1	R3-1h-Y	23684601	83.72
		Leaf	4 hours after freezing (at -10 ℃)	1	R3-4h-Y	26221665	83.95
		Leaf	8 hours after freezing (at -10 ℃)	1	R3-8h-Y	26490091	83.69
Genes acting in drought response	Weining	Root	3 hours after drought	1	R3-3h-G	23386435	81.92
		Root	6 hours after drought	1	R3-6h-G	25290369	80.84
		Root	12 hours after drought	1	R3-12h-G	31347004	80.78
		Leaf	3 hours after drought	1	R3-3h-Y	23737660	83.33
		Leaf	6 hours after drought	1	R3-6h-Y	21501245	81.76
		Leaf	12 hours after drought	1	R3-12h-Y	27472050	84.09
Genes related to heading date determination	Weining	Leaf	4 days after sowing	3	WN4	28925265	87.45
		Leaf	7 days after sowing	3	WN7	27322030	87.63
		Leaf	10 days after sowing	3	WN10	28834831	87.47
	Jingzhou	Leaf	4 days after sowing	3	JZ4	27895940	83.69
		Leaf	7 days after sowing	3	JZ7	28516674	83.53
		Leaf	10 days after sowing	3	JZ10	30556639	83.61

[1]Each of the values shaded in green or brown is the mean of four or three biological repeats.

Supplementary Table 12. Features of the PacBio transcriptomes sequenced in this study

**Target gene**	**Genotype**	**Tissue**	**Treatment/stage**	**Biological repeat**	**Sample ID**	**Bam file size (Gb)**	**Number of circular consensus reads**
Genes acting in vegetative and reproductive organs	Weining	Root	Normal condition	4	Mixed organs	29.0	573,461
		Stem	Normal condition	4			
		Leaf	Normal condition	4			
		Spike	Normal condition	4			
		Grain	10 days after anthesis	4	Mixed grains	31.0	296,749
		Grain	20 days after anthesis	4			
		Grain	30 days after anthesis	4			
		Grain	40 days after anthesis	4			

Supplementary Table 13. Statistics of gene features of 10 grass genomes/subgenomes[1]

	**Sc**	**Tu**	**Aet**	**Hv**	**TaA**	**TaB**	**TaD**	**Bd**	**Os**	**Sb**	**Zm**
HC gene Number	45,596	41,493	39,635	39,731	35,345	35,643	34,212	34,310	39,054	34,118	39,591
Gene length Mean	4,908.01	3,331.61	4,975.37	6,011.01	3,473.55	3,604.24	3,482.85	3,373.19	2,854.37	3,713.95	4,162.53
Gene length Median	2,774	2,346	2,734	2,259	2,374	2,436	2,428	2,617	2,188	2,824	2,556
Transcript Number	84,179	61,776	258,917	219,060	43,698	44,260	42,828	52,972	48,894	47,110	131,585
Transcript length Mean	1,670.71	1,554.37	1,812.13	1,845.88	1,218.55	1,259.28	1,259.99	2,185.49	1,552.74	2,211.95	2,614.84
Transcript length Median	1,466	1,348	1,522	1,552	1,041	1,083	1,083	1,793	1,413	1,766	2,151
CDS length Mean	1,221.99	1,012.26	1,020.68	1,058.34	1,218.55	1,259.28	1,259.99	1,260.06	1,079.99	1,228.18	1,446.86
CDS length Median	1,053	774	798	840	1,041	1,083	1,083	1,035	888	1,014	1,041
Exon number Mean	4.89	5.64	6.28	6.58	4.47	4.45	4.50	5.42	4.89	5.12	9.20
Exon number Median	3	4	5	5	3	3	3	3	3	3	7
Exon length Mean	341.78	275.78	288.57	280.46	272.43	283.09	280.12	403.58	317.23	431.66	284.09
Exon length Median	167	152	140	137	142	145	144	156	159	161	141
Intron length Mean	812.35	459.39	573.54	647.69	503.10	527.89	484.38	401.11	396.08	468.87	581.85
Intron length Median	134	136	127	132	135	135	134	141	160	143	131

[1]Sc, *Secale cereale* (Weining); Tu, *T. urartu* (G1812); Aet, *Ae. tauschii* (AL8/78); Hv, *Hordeum vulgare* (Morex); TaA, TaB and TaD, the A, B and D subgenomes of common wheat (*T. aestivum*, Chinese Spring); Bd, *B. distachyon* (Bd21); Os, *O. sativa* ssp. *japonica* (Nipponbare); Sb, *Sorghum bicolor* (BTx623); Zm, *Zea mays* (B73).

Supplementary Table 14. Statistics of TE annotation in Weining genome assembly

Class	Superfamily	Abbr.	Percentage (%)		Number		Family number	Total length (Mb)
			TEs	Assembled genome[a]	Intact	Truncated		
**Class I**	**LTR**							
	Gypsy	RLG	60.79	54.90	177,152	1,027,641	63	4,249.9
	Copia	RLC	16.95	15.30	72,941	237,069	40	1,184.6
	Unclassified	RLX	6.75	6.09	23,401	139,723	27	471.6
	**Non-LTR**							
	LINE	RIX	1.28	1.16	11,502	84,044	57	89.6
	SINE	RSX	0.005	0.0039	945	1,307	3	0.3
**Class II**	**DNA TE**							
	CACTA	DTC	11.68	10.55	19,072	405,085	50	816.5
	Mutator	DTM	0.43	0.39	15,320	46,189	43	29.9
	Unclassified TIRs	DTX	0.27	0.25	34,031	29,209	67	19.1
	PIF/Harbinger	DTH	0.24	0.22	14,012	22,391	17	17.1
	Tc1/Mariner	DTT	0.20	0.18	70,803	24,854	40	13.9
	Unclassified	DXX	0.03	0.03	1,218	3,940	6	2.2
	hAT	DTA	0.19	0.17	113	4,757	3	13.4
	**Helitron**	DHH	0.01	0.0078	25	875	1	0.6
**Others**		XXX	1.17	1.06	69,806	134,516	120	81.9
**Total**			100.00	90.31	510,341	2,161,600	537	6,990.6

[a]Calculated based on the assembled genome size (7.74 Gb).

Supplementary Table 15. Syntenic chromosomal segments between Weining rye and rice

**Rye (Weining)**			**Rice (Nipponbare)**			**Syntenic gene pairs**
Chr.	Start (Mb)	End (Mb)	Chr.	Start (Mb)	End (Mb)	
1R	69.60	210.19	Chr5	1.46	5.30	216
1R	211.19	599.90	Chr10	15.61	23.20	498
1R	600.23	940.97	Chr5	17.63	29.79	595
2R	11.40	25.03	Chr4	8.31	8.95	18
2R	25.03	600.32	Chr7	28.08	0.11	748
2R	600.32	1150.54	Chr4	18.17	34.83	780
3R	44.00	1094.84	Chr1	0.02	40.65	1852
4R	0.17	91.15	Chr3	23.54	29.29	401
4R	91.15	514.00	Chr11	0.58	9.20	239
4R	514.00	534.05	Chr3	11.98	12.50	38
4R	536.08	616.15	Chr8	24.40	26.55	114
4R	616.15	931.18	Chr6	0.25	11.88	483
4R	931.18	1043.78	Chr2	0.01	0.18	18
5R	8.43	390.29	Chr12	0.00	26.69	463
5R	391.56	743.67	Chr9	0.29	22.52	830
5R	745.39	998.91	Chr3	0.17	25.25	496
6R	3.45	781.95	Chr2	1.21	35.42	1310
6R	781.95	911.06	Chr1	41.25	43.13	99
6R	911.06	1034.47	Chr6	26.39	31.02	65
7R	7.00	346.31	Chr3	2.39	11.96	958
7R	346.31	644.30	Chr8	0.10	28.13	321
7R	644.30	976.50	Chr6	13.11	29.34	368
7R	976.50	1009.67	Chr4	0.12	5.73	39

Supplementary Table 16. Syntenic chromosomal segments between Weining rye and the three subgenomes of Chinese Spring wheat

Rye (Weining)			Chinese Spring A subgenome				Chinese Spring B subgenome				Chinese Spring D subgenome			
Chr.	Start (Mb)	End (Mb)	Chr.	Start (Mb)	End (Mb)	Syntenic gene pairs	Chr.	Start (Mb)	End (Mb)	Syntenic gene pairs	Chr.	Start (Mb)	End (Mb)	Syntenic gene pairs
1R	9.99	939.73	1A	4	589.63	3140	1B	4.97	686.85	3021	1D	3.12	491.63	3066
2R	10.65	1136.49	2A	24.28	780.78	3450	2B	36.85	801.22	3341	2D	22.45	650.78	3503
3R	6.35	1087.62	3A	0.02	683.36	3025	3B	3.31	750.86	3182	3D	0.7	560.36	3351
4R	0.17	534.05	4A	599.9	174.16	1695	4B	0.23	380.65	1478	4D	0.41	304.69	1431
4R	536.08	962.62	7A	0	258.37	1445	7B	2.1	337.64	1333	7D	1.02	241.1	1315
4R	967.31	1041.84	6A	0.63	35.26	299	6B	1.41	64.45	313	6D	1.59	31.85	385
5R	2.69	912.63	5A	7.51	650.83	3400	5B	9.29	646.48	3205	5D	9.28	528.73	3154
5R	918.92	985.14	5A	663.19	709.53	452	4B	617.28	673.53	413	4D	480.8	509.48	514
6R	6.64	786.21	6A	37.42	613.63	1924	6B	69.71	712.35	2039	6D	36.58	467.2	2107
6R	787.26	912.32	3A	698.35	749.85	432	3B	751.75	820.06	466	3D	564.14	613.6	458
6R	914.25	1022.81	7A	686.94	733.51	399	7B	672.76	735.31	327	7D	594.15	631.43	325
7R	5.85	64.22	4A	603.67	640	313	5B	670.93	710.24	255	5D	533.2	561.93	352
7R	68.19	346.31	4A	4	173.93	1013	4B	380.72	607.44	1006	4D	305.06	479.97	1134
7R	347.14	962.48	7A	254.88	676.07	1439	7B	216.82	654.92	1581	7D	241.18	585.56	1405
7R	970.35	1008.4	2A	2.37	20.86	222	2B	1.71	49.91	252	2D	2.22	18.75	330

Supplementary Table 17. Classification by MCScanX program of the HC genes in Weining rye and six other grass species[1]

**Category**	**Sc**	**Tu**	**Aet**	**Hv**	**TaA**	**TaB**	**TaD**	**Bd**	**Os**
Singleton	4,217	9,555	4,514	4,502	4,266	3,759	3,816	8,835	10,609
Dispersed duplicated	23,753	20,007	23,871	23,178	18,376	18,603	17,926	15,518	15,190
Proximal duplicated	6,659	3,402	3,223	4,172	2,752	3,508	2,886	2,064	2,934
Tandem duplicated	7,077	4,581	4,132	3,767	5,858	6,553	6,165	3,097	3,982
Segmental duplicated	1,866	505	3,035	1,945	4,023	3,138	3,368	4,795	6,137
Total	43,572	38,050	38,775	37,564	35,275	35,561	34,161	34,302	38,852

[1]Sc, *Secale cereale* (Weining); Tu, *T. urartu* (G1812); Aet, *Ae. tauschii* (AL8/78); Hv, *Hordeum vulgare* (Morex); TaA, TaB and TaD, the A, B and D subgenomes of common wheat (*T. aestivum*, Chinese Spring); Bd, *B. distachyon* (Bd21); Os, *O. sativa* ssp. *japonica* (Nipponbare).

Supplementary Table 18. Comparison of starch biosynthesis related genes in Weining rye and Chinese Spring wheat

**Gene**[1]	**Protein**	**Gene ID in Weining rye**	**Chromosomal location in Weining rye**	**Type of duplication**	**Chromosome location in common wheat**
ScAGP-L2-P	ADP glucose pyrophosphorylase-L2-plastidic	ScWN5R01G581600<br>ScWN5R01G582200	5RL, 5RL	Proximally duplicated	5AL 5BL 5DL
ScAGP-S2-P	ADP glucose pyrophosphorylase-S2-plastidic	ScWN5R01G184700	5RL		5BL 5DL[2]
ScAGP-L1-C	ADP glucose pyrophosphorylase-L1-cytoplasmic	ScWN1R01G556700	1RL		1AS 1BS 1DS
ScAGP-S1-C	ADP glucose pyrophosphorylase-S1-cytoplasmic	ScWN7R01G268800	7RS		7AS 7BS 7DS
ScGBSSI	Granule bound starch synthase I	ScWN4R01G463600	4RL		7AS 4AL 7DS
ScGBSSII	Granule bound starch synthase II	ScWN2R01G422600	2RL		2AL 2BL 2DL
ScSSI	Starch synthase I	ScWN4R01G394300	4RL		7AS 7BS 7DS
ScSSIIa	Starch synthase IIa	ScWN4R01G311700	4RL		7AS 7BS 7DS
ScSSIIb	Starch synthase IIb	ScWN6R01G257000	6RL		6AL 6BL 6DL
ScSSIIc	Starch synthase IIc	ScWN1R01G185100	1RS		1AL 1BL 1DL
ScSSIIIa	Starch synthase IIIa	ScWN1R01G330400<br>ScWN1R01G500200<br>ScWN4R01G199100	1RL, 1RL, 4RL	Dispersed duplicated	1AS 1BS 1DS
ScSSIIIb	Starch synthase IIIb	ScWN2R01G517800	2RL		2AL 2BL 2DL
ScSSIV	Starch synthase IV	ScWN1R01G467000<br>ScWN1R01G486700	1RL	Transposed duplicated	1AL 1BL 1DL
ScSBEI	Starch branching enzyme I	ScWN6R01G583300<br>ScWN6R01G584400	6RL	Proximally duplicated	7AL 7BL 7DL
ScSBEIIa	Starch branching enzyme IIa	ScWN2R01G323200	2RL		2AL 2BL 2DL
ScSBEIIb	Starch branching enzyme IIb	ScWN2R01G342700	2RL		2AL 2BL 2DL
ScSBEIII	Starch branching enzyme III	ScWN7R01G337200	7RL		7AL 7BL 7DL
ScISA1	Isoamylase I	ScWN4R01G237900	4RL		7AS 7BS 7DS
ScISA2	Isoamylase II	ScWN1R01G341700	1RL		1AL 1BL 1DL
ScISA3	Isoamylase III	ScWN5R01G272000	5RL		5AL 5BL 5DL
ScPUL	Pullulanase	ScWN4R01G378700	4RL		7AS 7BS 7DS
ScPHO1	Plastid alpha-1,4-glucan phosphorylase I	ScWN5R01G446600	5RL		5AL 5BL 5DL

ScPHO2	Plastid alpha-1,4-glucan phosphorylase II	ScWN3R01G454000<br>ScWN3R01G454100	3RL	Tandemly duplicated	3AL 3BL 3DL
ScDPE1	Disproportionating enzyme	ScWN2R01G161500<br>ScWN2R01G159200	2RS	Transposed duplicated	2AS 2BS 2DS
ScGPT1	Glucose-6-phosphate translocator	ScWN7R01G269800	7RS		7AS 7BS 7DS
ScSuSy1	Sucrose synthase 1	ScWN5R01G436200<br>ScWN4R01G349100	4RL, 5RL	Transposed duplicated	7AS 7BS 7DS
ScSuSy2	Sucrose synthase 2	ScWN2R01G169900<br>ScWN2R01G524700<br>ScWN4R01G484200	2RS, 2RL, 4RL	Transposed duplicated	2AS 2BS 2DS
ScUGPase1	UDP-glucose pyrophosphorylase	ScWN5R01G327700<br>ScWN5R01G396700	5RL, 5RL	Transposed duplicated	5AL 5BL 5DL
ScUGPase2	UDP-glucose pyrophosphorylase	ScWN4R01G563300	4RL		6AS[2]
ScSPSI	Sucrose-phosphate synthase	ScWN4R01G096500	4RS		4AL 4BS 4DS
ScSPSII	Sucrose-phosphate synthase	ScWN3R01G021900	3RS		3AS 3DS[2]
ScSPSIII	Sucrose-phosphate synthase	ScWN6R01G072300	6RS		6AS 6BS 6DS
ScSPSIV	Sucrose-phosphate synthase	ScWN6R01G544800	6RL		7AL 7BL 7DL
ScSPSV	Sucrose-phosphate synthase	ScWN3R01G512400	3RL		3AL 3BL 3DL
ScSUT1	Sucrose transporter	ScWN7R01G119300	7RS		4AS 4BL 4DL
ScSUT2	Sucrose transporter	ScWN5R01G020700	5RS		5BS 5DS[2]

[1]The genes highlighted in red was duplicated once or twice in Weining rye genome relative to the three subgenomes of Chinese Spring.

[2]Some of the starch biosynthesis related genes in Chinese Spring lacked one or two homoeologs. These missing homoeologs had been annotated as low confidence genes in the genome assembly of Chinese Spring (v1.0).

Supplementary Table 19. Summary of secalin genes annotated in Weining genome assembly

Locus	Gene name	Gene ID	Open reading frame	Predicted molecular mass (kDa)[1]	Chromosome location	Start	End	Strand	Supporting PacBio transcriptome reads
Sec-1	*40k gamma-secalin 1*	-	Disrupted	-	1R	10191653	10192549	+	-
	40k gamma-secalin 2	-	Disrupted	-	1R	10224852	10225763	+	-
	40k gamma-secalin 3	-	Disrupted	-	1R	10243802	10244703	+	-
	40k gamma-secalin 4	-	Disrupted	-	1R	10246636	10247541	+	-
	40k gamma-secalin 5	ScWN1R01G011410	Intact	34.15	1R	10268543	10269448	+	49
	40k gamma-secalin 6	ScWN1R01G011420	Intact	34.19	1R	10287264	10288169	+	51
	40k gamma-secalin 7	-	Disrupted	-	1R	10292504	10293400	+	-
	40k gamma-secalin 8	ScWN1R01G011810	Intact	34.16	1R	10297739	10298644	+	43
	40k gamma-secalin 9	ScWN1R01G011820	Intact	34.12	1R	10302986	10303891	+	31
	Omega secalin 1	-	Disrupted	-	1R	22150165	22151237	+	-
	Omega secalin 2	ScWN1R01G031890	Intact	41.19	1R	22173362	22174435	+	9
	Omega secalin 3	ScWN1R01G031900	Intact	41.55	1R	22164116	22165188	+	42
Sec-2	*75k gamma-secalin 1*	ScWN2R01G004010	Intact	52.70	2R	2510282	2511677	-	84
	75k gamma-secalin 2	ScWN2R01G004020	Intact	54.29	2R	2519784	2521218	-	98
	75k gamma-secalin 3	ScWN2R01G004030	Intact	53.64	2R	2542224	2543640	-	81
Sec-3	*Glu-1Ry*	ScWN1R01G430800	Intact	80.77	1R	793164916	793167542	+	68
	Glu-1Rx	ScWN1R01G431000	Intact	81.48	1R	793201484	793203161	+	147
Sec-4	*40k gamma-secalin 10*	ScWN1R01G083810	Intact	34.22	1R	83209223	83210128	+	176
	Omega secalin 4	ScWN1R01G085210	Intact	55.39	1R	83801733	83803163	-	5

[1]The deduced amino acid sequences of the 13 active secalin genes are listed below.

>40k gamma-secalin 5

MKTFLILTILAMATTIATANMQVGPSGQVEWPEQQPLPQPQQPVYQQPQQIFPQPQQTFPLEPQQTFPQPQQTFPHQPPQQFPQAQQPEQPFPEPQQTFPQQPQLPFPQQPQQPLPQPQQPQQPFPQPQQPQQPFPQPQQQFPQP
QQPQQSIPQQQQPLIQSSLQQQMNPCKNFLLQQCNPVSLVSSLVSLILPRSDCQVMQQQCCQQLAQIPQHLQCAAIHSVAHSIIMQQQQQQGIQILRPLFQIVQGQSIIQQQPAQLEVIRSLVLKTLPTMCNVYVRPDCSNIRTPF
ASTVAGIGGQ

>40k gamma-secalin 6

MKTFLILTILAMATTIATANMQVGPSGQVEWPQQQPLPQPQQPVYQQPQQIFPQPQQTFPLEPQQTFPQPQQTFPHQPPQQFPQAQQPQQPFPQPQQTFPQQPQLPFPQQPQQPLPQPQQPQQPFPQPQQPQQPFPQPQQQFPQP

QQPQQSIPQQQQPLIQSSLQQQMNPCKNFLLQQCNPVSLVSSLVSLILPRSDCQVMQQQCCQQLAQIPQHLQCDAIHSVAHSIIMQQQQQQGIQILRPLFQIVQGQSIIQQQPAQLEVIRSLVLKTLPTMCNVYVRPDCSNIRTPF
ASTVAGIGGQ

>40k gamma-secalin 8

MKTFLILTILAMATTIATANMQVGPSGQVEWPQQQPLPQPQQPVYQQPQQIFPQPQQTFPLEPQQTFPQPQQTFPHQPPQQFPQAQQPQQPFPQPQQTFPQQPQLPFPQQPQQPLPQPQQPQQPFPQPQQPQQTFPQPQQQFPQP
QQPQQSIPQQQQPLIQSSLQQQVNPCKNFLLQQCNPVSLVSSLVSLILPRSDCQVMQQQCCQQLAQITQHLQCAAIHSVAHSIIMQQQQQQGIQILRPLFQIVQGQSIIQQQPAQLEVIRSFVLKTLPTMCNVYVRPDCSNIRTPF
ASTVAGIGGQ

>40k gamma-secalin 9

MKTFLILTILAMATTIATANMQVGPSGQVEWPQQQPLPQPQQPVYQQPQQIFPQPQQTFPLEPQQTFPQPQQTFPHQPPQQFPQAQQPQQPFPQPQQTFPQQPQLPFPQQPQQPLPQPQQPQQPFPQPQQPQQTFPQPQQQFPQP
QQPQQSIPQQQQPLIQSSLQQQVNPCKNFLLQQCNPVSLVSSLVSLILPRSDCQVMQQQCCQQLAQIPQHLQCAAIHSVAHSIIMQQQQQQGIQILRPLFQIVQGQSIIQQQPAQLEVIRSLVLKTLPTMCNVYVRPDCSNIRTPFA
STVAGIGGQ

>40k gamma-secalin 10

MKTFLILTILAMATTIATANMQVGPSGQVEWPQQQPLPQPQQPVYQQPQQIFPQPQQTFPLEPQQTFPQPQQTFPHQPPQQFPQAQQPQQQFPQPQQTFPQQPQLPFPQQPQQPLPQPQQPQQTFPQSQQPQQPFPQTQQQFPQ
PQQPQQSIPQQQQPLIQSSLQQQMNPCKNFLLQQCNAVSLVSSIVSLILPRSDCQVMQQQCCQQLAQIPQHLQCAAIHSVVHSIIMQQQQQQGLQILRPLFQIVQGQSIIQQQLAQLEVIRSLVLKTLPTMCNVYVRPDCSTIRTP
FSSTIAGIGGQ

>Omega-secalin 2

MKTFLIFVLAMTMSIVTTARQLNPSEQKLQSPQQPVPKEQSYLQQPYPSHQPFPTPQQYSPYQPHQPFPQPQQPTPIQPQQPFPQQPQQPFPQPQQQLPLQPQQPFPQPQQLIPQQPHQSFPPQPQRPQQQFPQQPQQIIPQQTQQ
SFPLQPQQPFPQQPQRPFAQQPEQIISQQPFPLQPQQPFSQPQRPFPQQPGKIILQQPQQPSPLQPQQPFSQQPQRPQQTFPQQPQQIIPQQPQQPFPLQPQQPVPQQPQRPFGQQPEQIISQRPQQPFPLQPQQSFSQPQEPFPQQPG
QIIPQPPQQPFPLQPQQPFLQQTEQIISQQPQQPSPLQPQQPSPQQPQLPFPSPSNHLY

>Omega-secalin 3

MKTFLIFVLAMTMSIITTARQLNPSEQELQSPQQPVPKEQSYPQQPYPSHQPFPTPQQYSPYQPQQPFPQPQQPTPIQPQQPFPQQPQQPFPQPQQQLPLQPQQPFPQPQQPIPQQPQQSFPQQPQRPEQQFPQQPQQIIPQQTQQP
FPLQPQQPFPQQPQRPFAQQPEQIISQQPFPLQPQQPFSQPQQPFPQQPGQIIPQQPQQPSPLQPQQPFSQQPQRPQQPFPQQPQQIIPQQPQQPFPLQPQQPVPQQPQRPFGQQPEQIISQRPQQPFPLQPQQPFSQPQQPFPQQPGQ
IIPQQPQQPFPLQPQQPFPQQPEQIISQQPQQPFPLQPQQPSPQQPQLPFPQPQQPFVVVV

>Omega-secalin 4

MKTFLIFVLFAMAMSIATSARQLNPNNPQQLFSHHEQPFPQNPYPSQQPFPTPQQQLPQQSQQPFPQPQTQTPLQPQQPFPQQPQQPVPQPQQSFLQQPEQPFPQQPEQPSPQQPEQIIPQQPQQPSPLQPQQPFPQQPQQPFLLLP
KHIIPQQPQQRFLPLPEHIIPQQPQQQFSLQPQQPFLQQPQQPFPQQPQQPVPRPQQSSPQQPQQPFPLQPQQPFPQQPQQPFPQQPQQPSPQQPEQIIPQQPQQPSPLQPQQSFPQQPQQPFPQPQQPFLLLPKHIIPQQPQQRFLPL
PEHIIPQQPQQQFSLQPQQPFPQQPQQPFPQQPQQPTPQPQQPFPQHSQQQFPEPQQPQQPFPLQPQQQFPQQPQQPFPQQPQQPFPQQPQQPFPQKAQQSQQSIPQPQPQQLSPFQPQQLLPQQPQQPFLQPQQPLPQKPEQIIAQ
QPQQPLPQQPHQPQQPYPQQQPSRSSVTSIGGQ

>75k gamma-secalin 1

MKTLLMLAILAMATTIATANMQVNPSGQVQCPQQQPFPQPQKSSPQQPQQPFPQQSQQPFPQQPQQSSPQPLQPYPQQPFPQQPQQPYPQQPQQPFPQPPQQPYPQQPQQPFPQQPQQPVPQQPQQPVPQQPLQQFPQQPQQS
FPQQPQQPVPQQPLQQFPQQPQQPFPQQPQQPVPQQSQQSFPQTQQPQQPFPQPQQPQQLFPQPQLSSSQQPQQVTSQPQQPFPQAQPPQQSCPQSQQPYPQEPQQLFPQSQQPQQPFPQPQQPQQPFPQPQPQTQQSIPQPQQP
FPQYQEPFPQVHQPQQPSPQQQQPSIQLSLQQQLNPCKNVLLQQCSPVALVSSLRSKIFPQSECQVMQQQCCQQLAQIPQQLQCAAIHSVVHAIIMQQEQREGVQILLPQSHKQHVGQGALAQVQGIIQPQQLSQLEVVRSLVL
QNLPTMCNVYVPRQCSTIQAPFASIVTGIVGH

>75k gamma-secalin 2

MKTLLMLAILAMATTIATANMQVNPSGQVQCPQQQPFPQPQQSSPQQPQQPFPQQSQQPFPQQPQQSSPQPQQPYPQQPFPQQPQQPYPQQPQQPFPQQPQQPYPQQPQQPFPQQPQQPVPQQPQQQFPQQPQQPVPQQPLQ
QFPQQPQQSFPQQPQQPVPQQPLQQFPQQPQQPFPQQPQQPVPQQSQQPFPQTQQPQQPFPQPQQPQQLFPQTQQSSPQQPQQVTSQPQQPFPQAQPPQQSSPQSQQPYPQEPQQLFPQSQQPQQPFPQPQQPQQPFPQPQTQQ
SIPQPQQPFPQPQQPFPQSQEPFPQVHQPQQPSPQQQQPSIQLSLQQQLNPCKNVLLQQCSPVALVSSVRSKIFPQSECQVMQQQCCQQLAQIPQQLQCAAIHSVVHAIIMQQEQREGVQILLPQSHQQHVGQGALAQVQGIIQP
QQLSQLEVVRSLVLQNLPTMCNVYVPRQCSTIQAPFASIETGIVGH

>75k gamma-secalin 3

MKTLLMLAILAMATTIATANMQVNPSGQVQCPQQQPFPQPQQSSPQQPQQPFPQQSQQPFPQQPQQSSPQPQQPYPQQPFPQQPQQPYPQQPQQPFPQQPQQPYPQQPQQPFPQQPQQPVPQQPQQQFPQQPQQPVPQQPLQ
QFPQQPQQPFPQQPLQQFPQQPQQPFPQQPQQPVPQQSQQPFPQTQQPQQPFPQPQQPQQLFPQTQQSSPQQPQQVTSQPQQPFPQAQPPQQSSPQSQQPYPQEPQQLFPQSQQPQQPFPQPQQPQQPFPQPQPQTQQSIPQPQQ
PFPQPQQPFPQSQEQFPQVHQPQQPSPQQQQPSIQLSLQQQLNPCKNVLLQQCSPVALVSSLRSKIFPQSECQVMQQQCCQQLAQIPQQLQCAAIHSVVHAIIMQQEQREGVQILLPQSHQQHVGQGALAQVQGIIQPQQLSQL
EVVRSLVLQNLPTMCNVYVPRQCSTIQAPFASIVTGIVGH

>Glu-1Ry

MAKRLVLFATVVIALVALTAAEGEASGQLQCERELQESSLEACRQVVDQQLAGRLPWSTGLQMRCCQQLRDVSAKCRHVAVSQVARQYEQTTVPPKGGSFYPSETTPLQQLQQGIFWGTSSQTVQGYYPSVTSPQQGSYYP
GQASPQQPGQGQQPGKWQEPGQWQQGYYPTSLQQPGQGQQEHYPASQQHPRQGQQGHYPASLQQPGQGQQTRQPGQIQQPGQGQQIEQAQQPEQEQQPGQGQQGYYSTPPQQPGQGQQPGQGQQGYYPTSLQQPGQG
QPGQRQQPGQGQQIGQGQQPEQEQQPGQGQQGYYPASLQEPGQGQQEHYPASQQQPGQGQQGHYPASPQQPGQGRQGHYPASLQHPGQGQQTEQPGQGQQPEEGQQPEQEQQLGQGQQGYYPTSPQQPGQGQQPGQG
QQGYYSTSLQQPGQGQQGHYPTSLQQPGQGQPGQRQQLGQGQQPGQGQQPEQEQQPGQGQQGYYPTSPQQPGQGQQPGQGQQGYYSTSLQQPGQGQQGHYPTSLQQPGQGQPGQRQQPGQGQQIGQGQQPEQEQQPG
QGQQGHYPASLQQPGQGQQTEQPGQGQQLGQGQQPGQGKHPEQEQQPGQWQKGYYPTSQQQPGQGQQPGQWQQPGQGQQGHCPTSRQQPGQAQQPGQGQQIGQAQKPGQGQQGHYPTSLQHPGQLQQSGQGQQSG
QGHQPGQGQQSGQDQQGHDSPCHVSAEQQATSPKVAKAQQPVAQLPAMCRMEAGDALSASQ

>Glu-1Rx

MAKRLVLFAAVVVALVALTVAEGEASGQLQCERELQERELEACRQIVDQQLRDTSPGCRPVAVSPGTGQQEQQTVVPLKGGSFYPDETSPPQQLEQRILWGIPTLLKRYYPSVTSPHQGSYYPGQTSLQQPGQAQQPGQGQQP
GQAQQPGHGQQSGQGQQPEKGQQGYYPTTPQQPGQGQQPGQGQPGYYLTSSQQPGQGQQPGQGQPGYYPTSSQQLGQGQQLGQGQQGQQPGQGQPGYYPTSPQQPGQGQQPGQGQQPGQGQQGQQPGQGQQGQQPG
QGQQPGEGQQGYYPTFPQQPGQVQQPGQGQQPGQGQPGYYPTSPQQPGQGQQPGQEQQPGQRQQPGQGKPGYYPTSPQQSGQGQSGYYPTSPQQPGQEQQPGQGQQVQQPGQGQQPGQGQQGYYPTSPQQSGQAQQP
GQWQQPGQGQSGYYPTSQQQPGQGQQPGQGQQPGQGQQPGQGQQGQQPGQGQQAGQGQQGYYPTSPQQLGQGQQPGQGQQPGQGQPGYYPTSPQQPGQGQQTGQGQQPGQGQQGQQPGQGQQPGQGQQGYYPTSP
QQPGQGQQPGQGQLEYYPTSPQQPGQGQPGYYPTFPQLPGQLQQPAQGQQGYYPTSPQQPGQGQQKYYPTSPQQPGQWQQPGQGQQGYYITSPQQSGQGQQPGQGQQPGQGQEGYYPTLGQQPGQWLQIGQGQQGYYP
TSPQQLGQGQQGYYLTSPQQPGQGKQPGQGQQSYDSPYHVSAEHQAASLKVAKAQQLAAQLPAMCRLEGGDALLASQ

Supplementary Table 20. Summary of GenBank accession numbers for the SSPs compared in Extended Data Fig. 6

Type	SSP	NCBI accession number or gene id	Species
HMW glutenins/HMW-secalins/ D-hordein	Ta HMW-GS Ax1	X61009.1	*Triticum aestivum*
	Ta HMW-GS Ax2	M22208.2	*Triticum aestivum*
	Ta HMW-GS Bx7	BK006773.1	*Triticum aestivum*
	Ta HMW-GS Bx14	AY367771.1	*Triticum aestivum*
	Ta HMW-GS By8	JF736014.1	*Triticum aestivum*
	Ta HMW-GS By9	X61026.1	*Triticum aestivum*
	Ta HMW-GS Dx2	X03346.1	*Triticum aestivum*
	Ta HMW-GS Dy12	BK006459.1	*Triticum aestivum*
	Aet HMW-GS-y	AET1Gv20756900	*Aegilops tauschii*
	Aet HMW-GS-x	AET1Gv20757200	*Aegilops tauschii*
	Sc HMW-SS 1Ry	ScWN1R01G430800	*Secale cereale*
	Sc HMW-SS 1Rx	ScWN1R01G431000	*Secale cereale*
	Hv D-hordein	HORVU1Hr1G066650	*Hordeum vulgare*
LMW glutenins/B-hordeins	Ta LMW-GS A2	MH347495.1	*Triticum aestivum*
	Ta LMW-GS B2	MH347496.1	*Triticum aestivum*
	Ta LMW-GS B3	MH347497.1	*Triticum aestivum*
	Ta LMW-GS B4	MH347498.1	*Triticum aestivum*
	Ta LMW-GS D1	MH347499.1	*Triticum aestivum*
	Ta LMW-GS D2	MH347500.1	*Triticum aestivum*
	Ta LMW-GS D3	MH347501.1	*Triticum aestivum*
	Ta LMW-GS D6	MH347502.1	*Triticum aestivum*
	Ta LMW-GS D7	MH347503.1	*Triticum aestivum*
	Ta LMW-GS D8	MH347504.1	*Triticum aestivum*
	Aet LMW-GS 1	AET1Gv20018600	*Aegilops tauschii*
	Aet LMW-GS 2	AET1Gv20026700	*Aegilops tauschii*
	Aet LMW-GS 3	AET1Gv20027600	*Aegilops tauschii*
	Aet LMW-GS 4	AET1Gv20028600	*Aegilops tauschii*
	Hv B-hordein-5	HORVU1Hr1G001120	*Hordeum vulgare*
	Hv B-hordein-6	HORVU1Hr1G001140	*Hordeum vulgare*
	Hv B-hordein-7	HORVU1Hr1G001350	*Hordeum vulgare*
	Hv B-hordein-8	HORVU1Hr1G001420	*Hordeum vulgare*
γ-gliadins/γ-secalins/γ-hordeins	Ta γ-gliadin A1	MH347507.1	*Triticum aestivum*
	Ta γ-gliadin A3	MH347517.1	*Triticum aestivum*
	Ta γ-gliadin A4	MH347508.1	*Triticum aestivum*
	Ta γ-gliadin B1	MH347509.1	*Triticum aestivum*
	Ta γ-gliadin B2	MH347510.1	*Triticum aestivum*
	Ta γ-gliadin B4	MH347511.1	*Triticum aestivum*
	Ta γ-gliadin B6	MH347512.1	*Triticum aestivum*
	Ta γ-gliadin D1	MH347513.1	*Triticum aestivum*
	Ta γ-gliadin D2	MH347514.1	*Triticum aestivum*
	Ta γ-gliadin D3	MH347515.1	*Triticum aestivum*
	Ta γ-gliadin D4	MH347516.1	*Triticum aestivum*
	Aet γ-gliadin 1	AET1Gv20016500	*Aegilops tauschii*
	Aet γ-gliadin 2	AET1Gv20016600	*Aegilops tauschii*
	Sc 40k γ-secalin 5	ScWN1R01G011410	*Secale cereale*
	Sc 40k γ-secalin 6	ScWN1R01G011420	*Secale cereale*
	Sc 40k γ-secalin 8	ScWN1R01G011810	*Secale cereale*
	Sc 40k γ-secalin 9	ScWN1R01G011820	*Secale cereale*
	Sc 40k γ-secalin 10	ScWN1R01G083810	*Secale cereale*
	Sc 75k γ-secalin 1	ScWN2R01G004010	*Secale cereale*

	Sc 75k γ-secalin 2	ScWN2R01G004020	*Secale cereale*
	Sc 75k γ-secalin 3	ScWN2R01G004030	*Secale cereale*
	Hv γ-hordein 2	HORVU1Hr1G000640	*Hordeum vulgare*
	Hv γ-hordein 3	HORVU1Hr1G000680	*Hordeum vulgare*
	Ta δ-gliadin B1	MH347505.1	*Triticum aestivum*
	Ta δ-gliadin D1	MH347506.1	*Triticum aestivum*
	Aet δ-gliadin 1	AET1Gv20016100	*Aegilops tauschii*
	Ta ω-gliadin B3	MH347522.1	*Triticum aestivum*
	Ta ω-gliadin B6	MH347518.1	*Triticum aestivum*
	Ta ω-gliadin D1	MH347519.1	*Triticum aestivum*
	Ta ω-gliadin D2	MH347520.1	*Triticum aestivum*
ω-gliadins/ω-secalins	Ta ω-gliadin D3	MH347521.1	*Triticum aestivum*
	Aet ω-gliadin 1	AET1Gv20013100	*Aegilops tauschii*
	Sc ω-secalin 2	ScWN1R01G031890	*Secale cereale*
	Sc ω-secalin 3	ScWN1R01G031900	*Secale cereale*
	Sc ω-secalin 4	ScWN1R01G085210	*Secale cereale*
	Ta α-gliadin A1	MH338193.1	*Triticum aestivum*
	Ta α-gliadin A2	MH338194.1	*Triticum aestivum*
	Ta α-gliadin A4	MH338195.1	*Triticum aestivum*
	Ta α-gliadin A5	MH338196.1	*Triticum aestivum*
	Ta α-gliadin A6	MH338197.1	*Triticum aestivum*
	Ta α-gliadin A8	MH338198.1	*Triticum aestivum*
	Ta α-gliadin A9	MH338199.1	*Triticum aestivum*
	Ta α-gliadin A10	MH338200.1	*Triticum aestivum*
	Ta α-gliadin B3	MH338182.1	*Triticum aestivum*
	Ta α-gliadin B7	MH338183.1	*Triticum aestivum*
	Ta α-gliadin B8	MH338184.1	*Triticum aestivum*
	Ta α-gliadin B9	MH338185.1	*Triticum aestivum*
	Ta α-gliadin B11	MH338186.1	*Triticum aestivum*
α-gliadins	Ta α-gliadin B14	MH338187.1	*Triticum aestivum*
	Ta α-gliadin B15	MH338188.1	*Triticum aestivum*
	Ta α-gliadin B16	MH338189.1	*Triticum aestivum*
	Ta α-gliadin B17	MH338190.1	*Triticum aestivum*
	Ta α-gliadin B18	MH338191.1	*Triticum aestivum*
	Ta α-gliadin B25	MH338192.1	*Triticum aestivum*
	Ta α-gliadin D1	MH338176.1	*Triticum aestivum*
	Ta α-gliadin D4	MH338177.1	*Triticum aestivum*
	Ta α-gliadin D5	MH338178.1	*Triticum aestivum*
	Ta α-gliadin D6	MH338179.1	*Triticum aestivum*
	Ta α-gliadin D8	MH338180.1	*Triticum aestivum*
	Ta α-gliadin D12	MH338181.1	*Triticum aestivum*
	Aet α-gliadin 1	AET6Gv20127000	*Aegilops tauschii*
	Aet α-gliadin 2	AET6Gv20125400	*Aegilops tauschii*

357

Supplementary Table 21. Comparison of transcription factor genes annotated in Weining rye and other grass genomes[1]

TF family	Sc	TaA	TaB	TaD	DUWA	DUWB	WEWA	WEWB	AA	DD	Bd	Hv	Os	Fold of increase in rye[2]
HB-other	32	20	22	21	32	16	20	15	32	20	12	20	14	
MADS-M-type	44	48	58	48	35	42	26	27	19	33	46	34	37	
C2H2	191	148 (1.29)	148 (1.29)	151 (1.26)	121 (1.58)	134 (1.43)	62 (3.08)	70 (2.73)	113 (1.69)	146 (1.31)	115 (1.66)	125 (1.53)	121 (1.58)	1.26 - 3.08
MYB	173	144 (1.20)	138 (1.25)	145 (1.19)	129 (1.34)	117 (1.48)	120 (1.44)	119 (1.45)	125 (1.38)	131 (1.32)	122 (1.42)	117 (1.48)	117 (1.48)	1.19-1.48
GRAS	67	62 (1.08)	55 (1.22)	63 (1.06)	58 (1.16)	52 (1.29)	55 (1.22)	57 (1.12)	43 (1.56)	55 (1.22)	63 (1.06)	62 (1.08)	60 (1.12)	1.06-1.56
B3	120	141	147	141	102	100	73	74	117	92	51	85	54	
C2C2-Dof	44	36 (1.22)	33 (1.33)	31 (1.42)	29 (1.52)	28 (1.57)	23 (1.91)	25 (1.76)	29 (1.52)	29 (1.52)	29 (1.52)	25 (1.76)	30 (1.47)	1.22-1.91
MADS-MIKC	47	40 (1.18)	41 (1.15)	44 (1.07)	41 (1.15)	38 (1.24)	31 (1.52)	28 (1.68)	39 (1.20)	36 (1.31)	33 (1.42)	33 (1.42)	35 (1.34)	1.07-1.68
HB-WOX	15	15	13	14	14	13	12	9	9	14	13	13	14	
AP2/ERF-AP2	27	20 (1.35)	20 (1.35)	21 (1.29)	17 (1.59)	18 (1.5)	19 (1.42)	19 (1.42)	16 (1.69)	23 (1.17)	23 (1.17)	14 (1.93)	14 (1.93)	1.17-1.93
LOB	42	31 (1.35)	29 (1.45)	29 (1.45)	26 (1.62)	28 (1.5)	26 (1.62)	26 (1.62)	27 (1.56)	29 (1.45)	28 (1.5)	32 (1.31)	36 (1.17)	1.17-1.62
PLATZ	13	16	14	16	15	13	10	12	14	13	14	9	15	
MYB-related	102	76 (1.34)	75 (1.36)	78 (1.31)	66 (1.55)	64 (1.59)	70 (1.46)	73 (1.40)	79 (1.29)	77 (1.32)	57 (1.79)	90 (1.13)	64 (1.59)	1.13-1.79
C3H	73	52 (1.40)	50 (1.46)	49 (1.49)	45 (1.62)	43 (1.70)	41 (1.78)	46 (1.59)	51 (1.43)	49 (1.49)	58 (1.26)	40 (1.83)	57 (1.28)	1.26-1.83
WRKY	115	100 (1.15)	86 (1.34)	109 (1.06)	90 (1.28)	83 (1.39)	79 (1.46)	73 (1.58)	92 (1.25)	96 (1.20)	89 (1.29)	102 (1.13)	94 (1.22)	1.06-1.58
bZIP	104	90 (1.16)	84 (1.24)	85 (1.22)	78 (1.33)	78 (1.33)	76 (1.37)	72 (1.44)	71 (1.46)	80 (1.30)	83 (1.25)	90 (1.16)	90 (1.16)	1.16-1.46
HB-KNOX	9	11	11	10	9	5	8	8	9	9	10	9	9	
DBP	8	4 (2.00)	4 (2.00)	3 (2.67)	4 (2.00)	4 (2.00)	4 (2.00)	4 (2.00)	6 (1.33)	4 (2.00)	5 (1.60)	4 (2.00)	4 (2.00)	1.33-2.67
bHLH	187	153 (1.22)	166 (1.13)	146 (1.28)	133 (1.41)	144 (1.30)	119 (1.57)	123 (1.52)	128 (1.46)	133 (1.41)	130 (1.44)	136 (1.38)	134 (1.40)	1.13-1.57
zf-HD	22	14	12	10	10	10	12	11	7	11	21	12	14	1.05-3.14

		(1.57)	(1.83)	(2.20)	(2.20)	(2.20)	(1.83)	(2.00)	(3.14)	(2.00)	(1.05)	(1.83)	(1.57)	
C2C2-GATA	28	29	25	25	23	26	24	25	28	26	29	21	25	
Alfin-like	9	5	6	6	6	6	6	6	6	6	9	8	9	
E2F-DP	11	9	9	9	9	9	8	9	8	9	11	9	7	
AP2/ERF-ERF	198	153 (1.29)	159 (1.25)	166 (1.19)	116 (1.71)	112 (1.77)	130 (1.52)	119 (1.66)	90 (2.20)	155 (1.28)	135 (1.47)	127 (1.56)	139 (1.42)	1.19-2.2
B3-ARF	26	23	21	23	20	22	22	23	20	24	26	22	27	
HB-HD-ZIP	46	38 (1.21)	35 (1.31)	37 (1.24)	38 (1.21)	31 (1.48)	36 (1.28)	29 (1.59)	32 (1.44)	34 (1.35)	37 (1.24)	34 (1.35)	40 (1.15)	1.15-1.59
LIM	6	5	6	6	5	6	4	4	5	4	6	2	6	
C2C2-YABBY	6	7	7	7	6	6	7	7	7	7	8	9	8	
Trihelix	33	28 (1.18)	30 (1.10)	27 (1.22)	23 (1.43)	27 (1.22)	24 (1.38)	25 (1.32)	20 (1.65)	26 (1.27)	26 (1.27)	25 (1.32)	26 (1.27)	1.1-1.65
HB-BELL	14	13	11	12	11	11	12	11	10	11	14	10	13	
OFP	44	33 (1.33)	32 (1.38)	33 (1.33)	31 (1.42)	30 (1.47)	28 (1.57)	29 (1.52)	19 (2.32)	34 (1.29)	32 (1.38)	28 (1.57)	31 (1.42)	1.29-2.32
NAC	165	158 (1.04)	147 (1.12)	143 (1.15)	130 (1.27)	112 (1.47)	115 (1.43)	112 (1.47)	111 (1.49)	139 (1.19)	128 (1.29)	137 (1.20)	135 (1.22)	1.04-1.49
GARP-G2-like	58	48 (1.21)	48 (1.21)	48 (1.21)	42 (1.38)	50 (1.16)	51 (1.14)	47 (1.23)	52 (1.12)	44 (1.32)	50 (1.16)	48 (1.21)	46 (1.26)	1.12-1.38
STAT	1	1	1	1	1	1	1	1	1	1	1	1	1	
SRS	5	5	5	5	5	4	5	5	3	5	6	5	5	
SBP	24	19 (1.26)	18 (1.33)	19 (1.26)	19 (1.26)	18 (1.33)	17 (1.41)	16 (1.50)	16 (1.50)	18 (1.33)	17 (1.41)	17 (1.41)	19 (1.26)	1.26-1.5
CSD	4	5	5	5	3	4	5	7	1	3	4	6	2	
TUB	15	12	12	12	12	12	12	12	15	12	12	24	15	
C2C2-LSD	5	4	4	5	4	5	5	5	4	5	5	6	4	
NF-YB	17	17	14	13	15	14	16	15	15	14	17	19	13	
NF-YC	16	13	15	14	12	14	11	10	11	16	15	11	16	
CPP	14	11	14	13	9	11	8	14	10	12	9	8	11	
TCP	27	20 (1.35)	21 (1.29)	20 (1.35)	15 (1.80)	15 (1.80)	22 (1.23)	18 (1.50)	16 (1.69)	23 (1.17)	21 (1.29)	20 (1.35)	20 (1.35)	1.17-1.80
VOZ	3	2	2	2	2	2	2	3	2	2	2	2	2	
HB-PHD	2	3	3	3	2	3	3	3	3	3	3	3	1	
HSF	31	27	27	22	24	30	22	22	25	27	24	24	25	1.03-1.41

		(1.15)	(1.15)	(1.41)	(1.29)	(1.03)	(1.41)	(1.41)	(1.24)	(1.15)	(1.29)	(1.29)	(1.24)	
AP2/ERF-RAV	14	7 (2.00)	8 (1.75)	9 (1.56)	6 (2.33)	7 (2.00)	9 (1.56)	9 (1.56)	7 (2.00)	9 (1.56)	4 (3.50)	8 (1.75)	4 (3.50)	1.56-3.5
EIL	7	6	7	7	6	7	7	7	5	7	6	7	9	
GeBP	13	10	13	13	7	9	10	10	10	13	15	15	17	
CAMTA	6	6	5	5	6	4	5	5	5	5	7	5	6	
Tify	24	18 (1.33)	19 (1.26)	16 (1.50)	11 (2.18)	16 (1.50)	14 (1.71)	11 (2.18)	12 (2.00)	18 (1.33)	15 (1.60)	10 (2.40)	17 (1.41)	1.26-2.4
NF-YA	12	6 (2.00)	6 (2.00)	7 (1.71)	7 (1.71)	5 (2.40)	7 (1.71)	7 (1.71)	7 (1.71)	7 (1.71)	7 (1.71)	7 (1.71)	11 (1.09)	1.09-2.4
BES1	8	6	7	6	6	6	9	8	8	5	8	9	6	
HRT	1	1	0	1	1	1	1	1	1	1	1	1	1	
GRF	14	12 (1.17)	8 (1.75)	10 (1.40)	11 (1.27)	9 (1.56)	9 (1.56)	8 (1.75)	6 (2.33)	10 (1.40)	12 (1.17)	13 (1.08)	12 (1.17)	1.08-2.33
S1Fa-like	2	1	1	1	1	1	1	1	1	0	1	0	2	
DBB	5	5	5	5	6	5	4	2	4	2	4	3	8	
RWP-RK	14	14	11	11	12	10	9	9	11	10	16	10	12	
C2C2-CO-like	12	11	10	11	9	10	9	9	9	10	10	5	11	
LFY	1	1	1	1	1	1	1	1	2	1	1	1	1	
NF-X1	5	2 (2.50)	3 (1.67)	2 (2.50)	3 (1.67)	3 (1.67)	3 (1.67)	3 (1.67)	2 (2.50)	3 (1.67)	2 (2.50)	2 (2.50)	2 (2.50)	1.67-2.5
ULT	1	1	1	1	1	1	1	1	0	1	1	2	2	
GARP-ARR-B	8	11	10	9	7	14	7	6	8	8	7	9	6	
BBR-BPC	2	3	1	2	3	1	3	1	2	2	3	2	4	
Whirly	2	3	1	2	3	1	3	1	2	2	2	2	2	

[1]Sc, *Secale cereale* (Weining); Tu, *T. urartu* (G1812); Aet, *Ae. tauschii* (AL8/78); Hv, *Hordeum vulgare* (Morex); DUWA and DUWB, the A and B subgenomes of durum wheat (*T. turgidum* ssp. *durum*, Svevo); WEWA and WEWB, the A and B subgenomes of wild emmer wheat (*T. turgidum* ssp. *dicoccoides*, Zavitan); TaA, TaB and TaD, the A, B and D subgenomes of common wheat (*T. aestivum*, Chinese Spring); Bd, *B. distachyon* (Bd21); Os, *O. sativa* ssp. *japonica* (Nipponbare).

[2]The families marked in brown had increased members in Weining rye. For example, 1.56-3.50 fold increase for AP2/ERF-RAV, 1.17-1.93 fold increase for AP2/ERF-AP2, and 1.19-2.20 fold increase for AP2/ERF-ERF.

Supplementary Table 22. Summary of disease resistance associated genes annotated in Weining rye and other grass genomes (subgenomes)[1]

Genome/ subgenome	NBS						TM-CC	RLP	RLK[2]	Total
	**CN**	**CNL**	**NBS**	**NL**	**TN**	**TX**				
Sc	45	245	45	238	4	3	142	129	1138	1,989[3]
TaA	88	218	96	263	3	2	117	133	916	1,836
TaB	175	247	53	267	3	2	97	124	760	1,728
TaD	78	226	83	244	3	2	115	154	983	1,888
Tu	74	160	156	235	3	2	101	116	774	1,621
Aet	45	196	72	242	3	1	130	145	924	1,758
Bd	30	149	33	128	1	4	106	57	670	1,178
Hv	37	136	59	176	2	2	154	113	829	1,508
Os	45	199	57	169	2	3	134	112	854	1,575

[1]Sc, *Secale cereale* (Weining); Tu, *T. urartu* (G1812); Aet, *Ae. tauschii* (AL8/78); Hv, *Hordeum vulgare* (Morex); DUWA and DUWB, the A and B subgenomes of durum wheat (*T. turgidum* ssp. *durum*, Svevo); WEWA and WEWB, the A and B subgenomes of wild emmer wheat (*T. turgidum* ssp. *dicoccoides*, Zavitan); TaA, TaB and TaD, the A, B and D subgenomes of common wheat (*T. aestivum*, Chinese Spring); Bd, *B. distachyon* (Bd21); Os, *O. sativa* ssp. *japonica* (Nipponbare).

[2]Receptor-like kinase genes were substantially increased in Weining rye compared to other species.

[3]Detailed information on the 1,989 disease resistance associated genes of Weining rye is provided in Supplementary Data 3.

Supplementary Table 23. List of gene specific primers used in this study

**Primer set**	**Target gene**	**Sequence (5′ -3′)**	**Use**
PS-ScFT1	*ScFT1*<br>(ScWN4R01G446100)	CAGGAGCTGATGTGCTACGA<br>GGGCGGGCCGAGGTTGTAGA	Analyzing *ScFT1* expression by qRT-PCR
PS-ScFT2	*ScFT2*<br>(ScWN3R01G192500)	ACAGGAGTATCTTTCGGGAC<br>CAGGCCGAGGTTGTAGAGCT	Analyzing *ScFT2* expression by qRT-PCR
PS-ScPpd1	*ScPpd1*<br>(ScWN2R01G043000)	GGAGGATCATGAACCACGA<br>CTCCAGCTGTGAGAGCGTCT	Analyzing *ScPpd1* expression by qRT-PCR
PS-ScID1	*ScID1.1*<br>(ScWN6R01G057200)<br>*ScID1.2*<br>(ScWN6R01G057300)	GGCATCCTCTTCTCCAGGAA<br>TTGTTGATGTCGTTGCTGCT	Analyzing *ScID1* expression by qRT-PCR
PS-ScFT2-PVX	*ScFT2*<br>(ScWN3R01G192500)	AGGTCAGCACCAGCTAGC<br>ATCGATATGGCCGGGAGGGACAGGGAC<br>CTTAACCGTTCATCGGCG<br>GTCGACTCATTCATTCAAGTCCTCTTC	Cloning ScFT2 and its derived mutants into the PVX viral vector
PS-ScActin	*ScActin*<br>(ScWN1R01G374800)	CAACGAGCTCCGTGTCGCA<br>GAGGAAGCGTGTATCCCTGTAG	As an internal control of qRT-PCR

Supplementary Table 24. Amino acid sequences of the 17 FT proteins from grasses and *Arabidopsis thaliana* used in the alignment shown in Supplementary Fig. 4c

Gene Name	NCBI accession number or gene id	Species	Amino acid sequence
ScFT1	ScWN4R01G446100	*Secale cereale*	MVGSGMQRGDPLVVGRVIGDVVDPF<br>ARRVALRVGYASRDVANGCELRPSAIA<br>DPPRVEVGGPDMRTFYTLVMVDPDAP<br>SPSDPSLREYLHWLVTDIPGTTGVSFG<br>TEVVCYEGPRPVLGIHRLVFLLFQQLG<br>RQTVYAPGWRQNFSTRDFAELYNLGL<br>PVAAVYFNCQRETGTGGRRM
ScFT2	ScWN3R01G192500	*Secale cereale*	MAGRDRDPLVVGRVVGDVLDPFVRT<br>TNLRVTFGNRAVSNGCELKPSMVAQQ<br>PRVEVGGNEMRTFYTLVMVDPDAPSP<br>SDPNLREYLHWLVIDIPGTTGASFGQE<br>LMCYESPRPTMGIHRFVLVLFQQLGR<br>QTVYAPGWRQHFNTREFAELYNLGPP<br>VAAVYFNCQREAGSGGRRMYN
HvFT1	AAZ38709.1	*Hordeum vulgare*	MAGRDRDPLVVGRVVGDVLDPFVRT<br>TNLRVTFGNRAVSNGCELKPSMVAQQ<br>PRVEVGGNEMRTFYTLVMVDPDAPSP<br>SDPNLREYLHWLVTDIPGTTGASFGQ<br>EVMCYESPRPTMGIHRFVLVLFQQLG<br>RQTVYAPGWRQNFNTRDFAELYNLG<br>QPVAAVYFNCQREAGSGGRRMYN
HvFT2	ABB99414.1	*Hordeum vulgare*	MVGSSMQRGDPLVVGRVIGDVVDPF<br>VRRVALRVGYASRDVANGCELRPSAIA<br>DQPRVEVGGPDMRTFYTLVMVDPDA<br>PSPSDPSLREYLHWLVTDIPATTGVSF<br>GTEVVCYEGPRPVLGIHRLVFLLFQQL<br>GRQTVYAPGWRQNFSTRDFAELYNLG<br>LPVAAVYFNCQRETGTGGRRM
AetFT1	XP_020200589.1	*Aegilops tauschii*	MVGSGMHAQRGDPLVVGRVIGDVVD<br>PFVRRVALRVGYASRDVANGCELRPS<br>AIADPPRVEVGGPDMRTFYTLVMVDP<br>DAPSPSDPSLREYLHWLVTDIPATTGV<br>SFGTEVVCYEGPRPVLGIHRLVFLLFQ<br>QLGRQTVYAPGWRQNFSTRDFAELYN<br>LGLPVAAVYFNCQRETGTGGRRM
AetFT2	XP_020200153.1	*Aegilops tauschii*	MAGRDRDPLVVGRVVGDVLDPFIRTT<br>NLRVTFGNRTVSNGCELKPSMVAQQP<br>RVEVGGNEMRTFYTLVMVDPDAPSPS<br>DPNLREYLHWLVTDIPGTTGASFGQE<br>VMCYESPRPTMGIHRFVLVLFQQLGR<br>QTVYAPGWRQNFNTRDFAELYNLGPP<br>VAAVYFNCQREAGSGGRRMYN
TaFT1-A	TraesCS7A02G115400.1	*Triticum aestivum*	MAGRDRDPLVVGRVVGDVLDPFVRT<br>TNLRVTFGNRTVSNGCELKPSMVAQQ<br>PRVEVGGNEMRTFYTLVMVDPDAPSP<br>SDPNLREYLHLVTDIPGTTGASFGQEV<br>MCYESPRPTMGIHRFVLVLFQQLGRQ<br>TVYAPGWRQNFNTRDFAELYNLGPPV<br>AAVYFNCQREAGSGGRRMYN
TaFT2-A	TraesCS3A02G143100.1	*Triticum aestivum*	MVGSGMQRGAPLVVGRVIGDVVDPF<br>VRRVALRVGYASRDVANGCELRPSAIA<br>DPPRVEVGGPDMRTFYTLVMVDPDAP<br>SPSDPSLREYLHWLVTDIPGTTGVSFD<br>ACPGTEVVCYEGPRPVLGIHRLVFLLF<br>QQLGRQTVYAPGWRQNFSTRDFAELY<br>NLGLPVAAVYFNCQRETGTGGRRM
TaFT1-B	TraesCS7B02G013100.1	*Triticum aestivum*	MAGRDRDPLVVGRVVGDVLDPFVRT<br>TNLRVTFGNRTVSNGCELKPSMVAQQ<br>PRVEVGGNEMRTFYTLVMVDPDAPSP

			SDPNLREYLHWLVTDIPGTTGASFGQEVMCYESPRPTMGIHRFVLVLFQQLGRQTVYAPGWRQNFNTRDFAELYNLGPPVAAVYFNCQREAGSGGRRMYN
TaFT2-B	TraesCS3B02G162000.1	*Triticum aestivum*	MVGSGMHAQRGDPLVVGRVIGDVVDPFVRRVALRVGYASRDVANGCELRPSAIADPPRVEVGGPDMRTFYTLVSSASAVRTSVRAMLARCLITPPRLLTPVSACAQVMVDPDAPSPSDPSLREYLHWLVTDIPATTGVSFGTEVVCYEGPRPVLGIHRLVFLLFQQLGRQTVYAPGWRQNFSTRDFAELYNLGLPVAAVYFNCQRETGTGGRRM
TaFT1-D	TraesCS7D02G111600.1	*Triticum aestivum*	MAGRDRDPLVVGRVVGDVLDPFIRTTNLRVTFGNRTVSNGCELKPSMVAQQPRVEVGGNEMRTFYTLVMVDPDAPSPSDPNLREYLHWLVTDIPGTTGASFGQEVMCYESPRPTMGIHRFVLVLFQQLGRQTVYAPGWRQNFNTRDFAELYNLGPPVAAVYFNCQREAGSGGRRMYN
TaFT2-D	TraesCS3D02G144500.1	*Triticum aestivum*	MVGSGMHAQRGDPLVVGRVIGDVVDPFVRRVALRVGYASRDVANGCELRPSAIADPPRVEVGGPDMRTFYTLVMVDPDAPSPSDPSLREYLHWLVTDIPATTGVSFATEVVCYEGPRPVLGIHRLVFLLFQQLGRQTVYAPGWRQNFSTRDFAELYNLGLPVAAVYFNCQRETGTGGRRM
OsFT1	XP_015641951.1	*Oryza sativa*	MAGSGRDRDPLVVGRVVGDVLDAFVRSTNLKVTYGSKTVSNGCELKPSMVTHQPRVEVGGNDMRTFYTLVMVDPDAPSPSDPNLREYLHWLVTDIPGTTAASFGQEVMCYESPRPTMGIHRLVFVLFQQLGRQTVYAPGWRQNFNTKDFAELYNLGSPVAAVYFNCQREAGSGGRRVYP
OsFT2	AFK31087.1	*Oryza sativa*	MAGSGRDRDPLVVGRVVGDVLDAFVRSTNLKVTYGSKTVSNGCELKPSMVTHQPRVEVGGNDMRTFYTLVMVDPDAPSPSDPNLREYLHWLVTDIPGTTAASFGQEVMCYESPRPTMGIHRLVFVLFQQLGRQTVYAPGWRQNFNTKDFAELYNLGSPVAAVYFNCQREAGSGGRRVYN
BdFT1	XP_003565602.1	*Brachypodium distachyon*	MVGGGMPRGDPLVVGRVIGDVVDPFVRRVSLRVGYASRDVANGCELRPSAIADPPRVEVGGPDMRTFYTLVMVDPDAPSPSDPSLREYLHWLVTDIPATTGVSFGTEVVCYESPRPVLGIHRLVFLLFQQLGRQTVYAPGWRQNFSTRDFAELYNLGLPVAAVYFNCQRESGTGGRRM
BdFT2	XP_003564300.1	*Brachypodium distachyon*	MAGRDRDPLVVGRVVGDVLDPFVRTTNLRVSFGNRNVSNGCELKPSMVTHQPRVEVGGNEMRTFYTLVMVDPDAPSPSDPNLREYLHWLVTDIPGTTGASFGQEVMCYESPRPSMGIHRFVFVLFQQLGRQTVYAPGWRQNFNTRDFAELYNLGPPVAAVYFNCQREAGSGGRRMYP
AtFT1	AT1G65480	*Arabidopsis thaliana*	MSINIRDPLIVSRVVGDVLDPFNRSITLKVTYGQREVTNGLDLRPSQVQNKPRVEIGGEDLRNFYTLVMVDPDVPSPSNPHLREYLHWLVTDIPATTGTTFGNEIVCYENPSPTAGIHRVVFILFRQLGRQTVYAPGWRQNFNTREFAEIYNLGLPVAAVFYNCQRESGCGGRRL
