# Supplementary material for: A high-quality genome assembly highlights rye genomic characteristics and agronomically important genes
Source: Nat Genet. 2021 Mar 18;53(4):574–84. doi: 10.1038/s41588-021-00808-z (PMC8035075; doi:10.1038/s41588-021-00808-z)
Supplement: Supplementary file 2 — Reporting Summary [file 41588_2021_808_MOESM2_ESM.pdf]

nature research

Corresponding author(s): Qinghua Yang, Kunpu Zhang, Jianping Yang, Daowen Wang

Last updated by author(s): Jan 12, 2021

Reporting Summary

Nature Research wishes to improve the reproducibility of the work that we publish. This form provides structure for consistency and transparency in reporting. For further information on Nature Research policies, see Authors & Referees and the Editorial Policy Checklist.

Statistics

For all statistical analyses, confirm that the following items are present in the figure legend, table legend, main text, or Methods section.

n/a	Confirmed	
☐	☒	The exact sample size (*n*) for each experimental group/condition, given as a discrete number and unit of measurement
☐	☒	A statement on whether measurements were taken from distinct samples or whether the same sample was measured repeatedly
☐	☒	The statistical test(s) used AND whether they are one- or two-sided<br>*Only common tests should be described solely by name; describe more complex techniques in the Methods section.*
☒	☐	A description of all covariates tested
☐	☒	A description of any assumptions or corrections, such as tests of normality and adjustment for multiple comparisons
☐	☒	A full description of the statistical parameters including central tendency (e.g. means) or other basic estimates (e.g. regression coefficient) AND variation (e.g. standard deviation) or associated estimates of uncertainty (e.g. confidence intervals)
☐	☒	For null hypothesis testing, the test statistic (e.g. *F*, *t*, *r*) with confidence intervals, effect sizes, degrees of freedom and *P* value noted<br>*Give P values as exact values whenever suitable.*
☒	☐	For Bayesian analysis, information on the choice of priors and Markov chain Monte Carlo settings
☒	☐	For hierarchical and complex designs, identification of the appropriate level for tests and full reporting of outcomes
☐	☒	Estimates of effect sizes (e.g. Cohen's *d*, Pearson's *r*), indicating how they were calculated

Our web collection on statistics for biologists contains articles on many of the points above.

Software and code

Policy information about availability of computer code

Data collection	No software was used in data collection.
Data analysis	Canu (version 1.5), WTDBG (https://github.com/ruanjue/wtdbg), FALCON (version 0.2.2), MECAT (version 1.3), Quickmerge (version 0.2), Pilon (version 1.22), Cutadapt (version 1.0), HiC-Pro (version 2.8.1), LACHESIS (https://github.com/shendurelab/LACHESIS), BWA (version 0.7.10-r789), GATK (version 3.3.0), SAMtools (version 1.9), VCFtools (version 0.1.13), HighMap (http://highmap.biomarker.com.cn/), RefAligner (6700.6902rel), BLASTN (version 2.7.1), BLASTP (version 2.7.1), R (version 3.5.1), BUSCO (version 3.0.2), LTR_retriever (version 2.8), RepeatScout (version 1.0.5), LTR-FINDER (version 1.0.5), MITE-hunter (http://target.iplantcollaborative.org/mite_hunter.html), PILER-DF (version 2.4), Repbase (version 19.06), REPET (version 2.5), CLARITE (https://github.com/jdaron/CLARI-TE), RepeatMasker (version 1.332), LTRharvest (version 1.5.10), IsoSeq3 (version 3.4.0), GeneWise (version 2.4.1), GeMoMa (version 1.3.1), Genscan (http://hollywood.mit.edu/GENSCANinfo.html), Augustus (version 2.4), GlimmerHMM (version 3.0.4), Hisat (version 2.0.4), Stringtie (version 1.2.3), BLAT (version 350), PASA (version 2.0.4). TopHat (version 2.0.13), Cufflinks (version 2.2.1), Transdecoder (version 2.0), EVidenceModeler (version 1.1.1), GO (Release 20180910), KEGG (Release 87.0), Swiss-Prot (Release 2015_01), TrEMBL (Release 2015_01), tRNAscan-SE (version 2.0), miRBase (Release 22), miRDeep2 (https://github.com/rajewsky-lab/mirdeep2), Infernal (version 1.1.2), OrthoMCL (version 1.1.4), MUSCLE (version 3.8.31), BEAST (version 2.5.1), DensiTree (version 2.2.5), MCScanX (http://chibba.pgml.uga.edu/mcscan2/), MCscan (Python version,https://github.com/tanghaibao/jcvi/wiki/MCscan-(Python-version)), iTAK(version 1.7a), RGAugury (https://bitbucket.org/yaanlpc/rgaugury/wiki/Home), IsoCon (version 1.0), MEGAX (https://www.megasoftware.net/), iTOL (https://itol.embl.de/), R/qtl (version 3.5.3),XP-CLR (version 1.0),funRiceGenes (https://funricegenes.github.io/),DupGen_finder (version 1.0),LTR_FINDER_parallel (https://github.com/oushujun/LTR_FINDER_parallel), pheatmap (version 1.0.12),R (version 3.5.1).<br><br>All codes used to generate the results reported in the study are available upon request.

For manuscripts utilizing custom algorithms or software that are central to the research but not yet described in published literature, software must be made available to editors/reviewers. We strongly encourage code deposition in a community repository (e.g. GitHub). See the Nature Research guidelines for submitting code & software for further information.

Data

Policy information about availability of data

All manuscripts must include a data availability statement. This statement should provide the following information, where applicable:

- Accession codes, unique identifiers, or web links for publicly available datasets
- A list of figures that have associated raw data
- A description of any restrictions on data availability

The Weining rye genome assembly has been deposited in NCBI GenBank under the accession number JADQCU000000000. The raw sequencing data have been deposited in the NCBI Sequence Read Archive (https://www.ncbi.nlm.nih.gov/sra/) under the BioProject accession numbers PRJNA680931, PRJNA680499 and PRJNA679094. The assembly and annotation data have also been submitted to the Chinese National Genomics Data Center (https://bigd.big.ac.cn/) under the accession number GWHASIY00000000. The Weining rye genome assembly and annotation are additionally available at the Triticeae Multi-omics Center (http://wheatomics.sdau.edu.cn/). Source data are provided with this paper.

Field-specific reporting

Please select the one below that is the best fit for your research. If you are not sure, read the appropriate sections before making your selection.

☒ Life sciences ☐ Behavioural & social sciences ☐ Ecological, evolutionary & environmental sciences

For a reference copy of the document with all sections, see nature.com/documents/nr-reporting-summary-flat.pdf

Life sciences study design

All studies must disclose on these points even when the disclosure is negative.

Sample size	No statistical methods were used to determine sample size. We incorporated a population of 295 F2 individuals derived from Weining and Jingzhou rye varieties to assist genome assembly and validation and to detect major QTLs for heading date. A genome wide genotyping-by-sequencing data set of 101 accessions of domesticated rye and wild Secale forms was employed for SNP calling and selection sweep analysis.
Data exclusions	No data were excluded from analysis. Raw sequencing data was quality filtered as described in manuscript.
Replication	Three replicates were executed for the qRT-PCR and immunoblotting experiments presented in Figure 6, Figure 7, Extended Data Figure 5, Extended Data Figure 9, and Supplementary Figure 5. All attempts at replication were successful. Other experiments not mentioned here were also reproducible over at least three separate trials.
Randomization	Plants were randomly allocated in the greenhouse and in the field.
Blinding	Blinding was not relevant for this study and phenotypic data were collected without knowledge of passport records or genetic data.

Reporting for specific materials, systems and methods

We require information from authors about some types of materials, experimental systems and methods used in many studies. Here, indicate whether each material, system or method listed is relevant to your study. If you are not sure if a list item applies to your research, read the appropriate section before selecting a response.

Materials & experimental systems

n/a	Involved in the study	
☐	☒	Antibodies
☒	☐	Eukaryotic cell lines
☒	☐	Palaeontology
☒	☐	Animals and other organisms
☒	☐	Human research participants
☒	☐	Clinical data

Methods

n/a	Involved in the study	
☒	☐	ChIP-seq
☒	☐	Flow cytometry
☒	☐	MRI-based neuroimaging

Antibodies

Antibodies used	A polyclonal antibody specific for rye FT protein was raised in rabbit using the peptide QLGRQTVYAPGWRQ conserved in ScFT1 and ScFT2 amino acid sequences by HuaBio (HuaAn Biotechnology Co., Ltd, Hangzhou, China, https://www.huabio.com). This antibody was used (in 1:2000) to detect the FT protein in rye leaf tissues by immunoblotting. As this antibody was self-made, it had no catalog number. The secondary antibody goat anti-Rabbit IgG H&L (IRDye® 800CW) was purchased from Abcam (https://www.abcam.com, Shanghai, China) and used in 1:5000 dilution in immunoblotting.
Validation	The peptide antibody was validated by its specific recognition of an in vitro expressed rye FT protein. Furthermore, a highly

similar peptide (QLGRQTVYAPGWRQN) has been used successfully to raise an antibody specific for Arabidopsis FT protein (Kim et al., 2016. Post-translational regulation of FLOWERING LOCUS T protein in Arabidopsis. Molecular Plant 9, 308-311).
