# Supplementary figures and images for: A high-quality genome assembly highlights rye genomic characteristics and agronomically important genes
Source: Nat Genet. 2021 Mar 18;53(4):574–84. doi: 10.1038/s41588-021-00808-z (PMC8035075; doi:10.1038/s41588-021-00808-z)

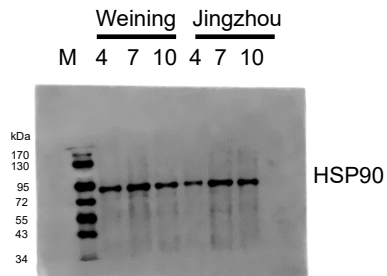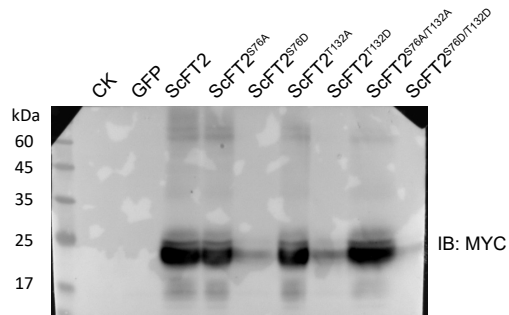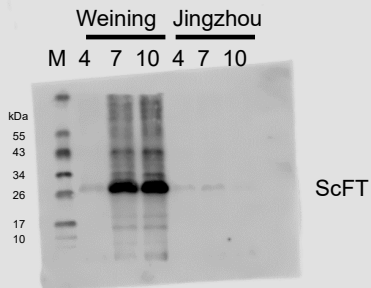

Loading control

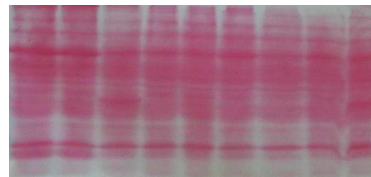

Supplement: Source Data Fig. 6 — Unprocessed western blots for Fig. 6. [file 41588_2021_808_MOESM8_ESM.pdf]

kDa

M

WN

180

135

98

75

62

48

34

25

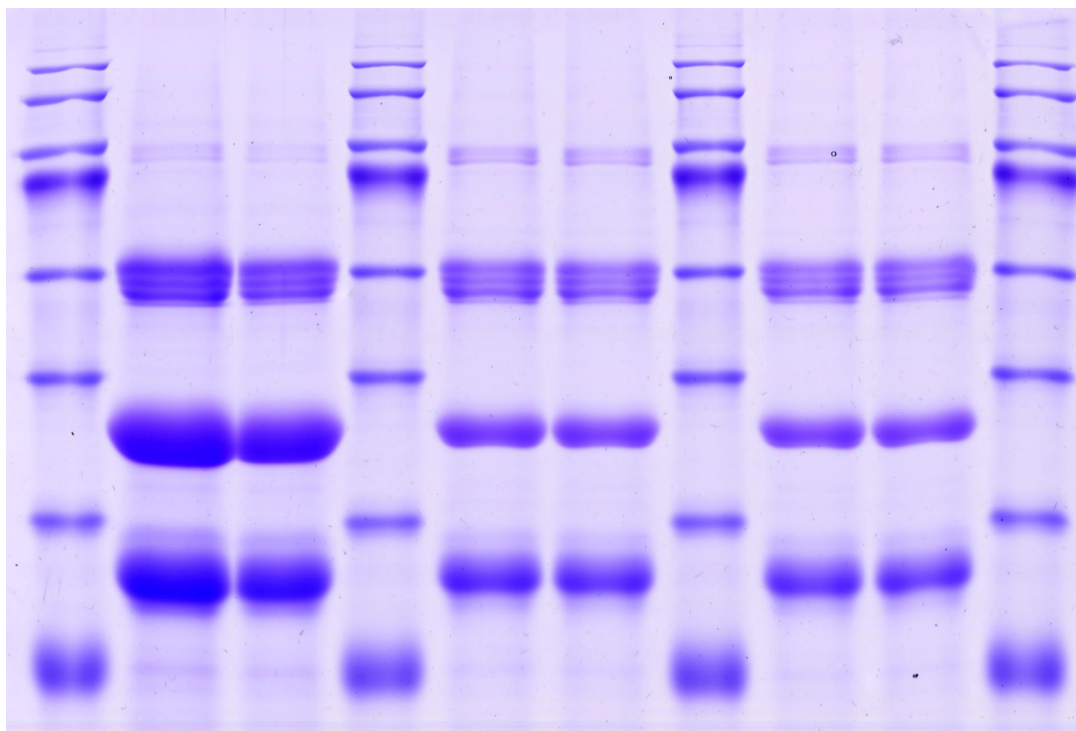

Supplement: Source Data Extended Data Fig. 5 — Unprocessed gels for Extended Data Fig. 5. [file 41588_2021_808_MOESM9_ESM.pdf]

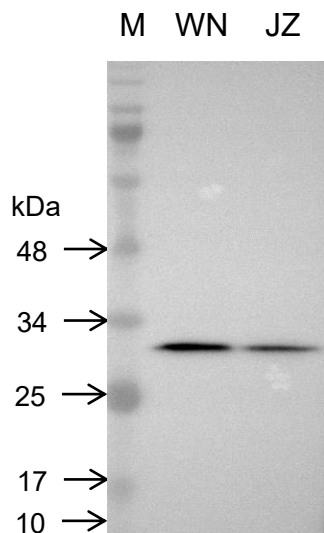

Anti: FT

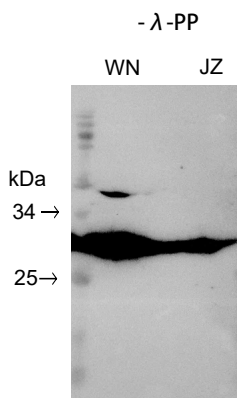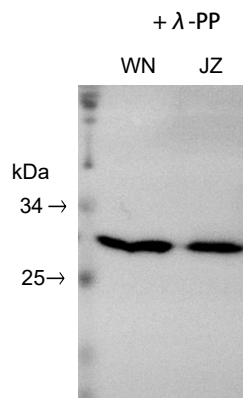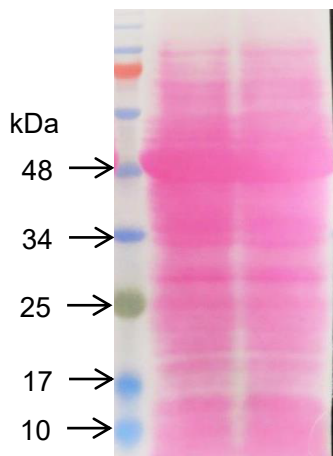

Loading  
control

Supplement: Source Data Extended Data Fig. 9 — Unprocessed western blots for Extended Data Fig. 9. [file 41588_2021_808_MOESM10_ESM.pdf]
